# Supplementary material for: Design and Synthesis of N-Substituted 3,4-Pyrroledicarboximides as Potential Anti-Inflammatory Agents
Source: Int J Mol Sci. 2021 Jan 30;22(3):1410. doi: 10.3390/ijms22031410 (PMC7866801; doi:10.3390/ijms22031410)
Supplement: Supplementary file 1 [file ijms-22-01410-s001.zip › qsar_molecular_docking.pdf]

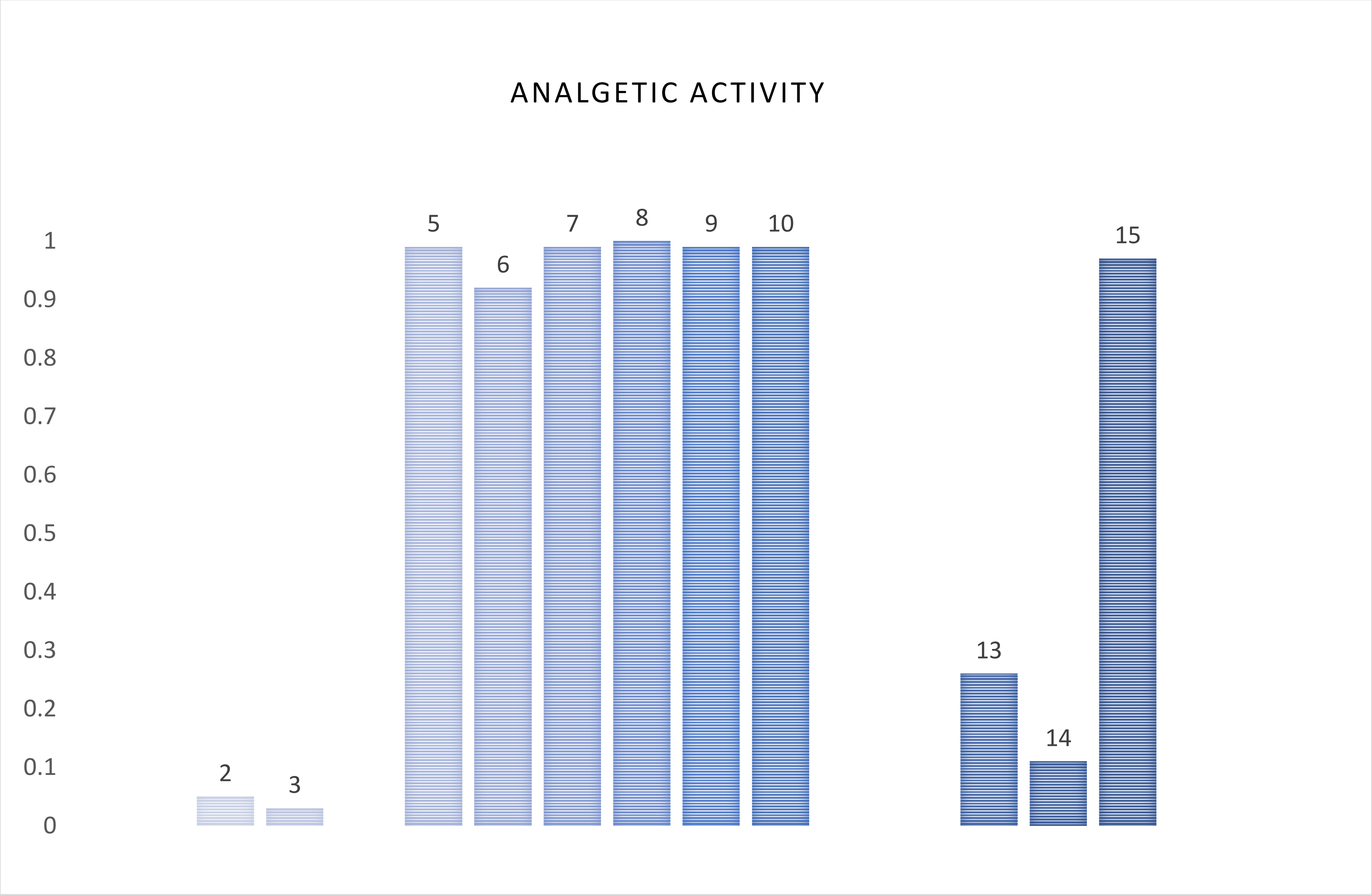
ANALGETIC ACTIVITY
1
0.9
0.8
0.7
0.6
0.5
0.4
0.3
0.2
0.1
0
2
3
5
6
7
8
9
10
13
14
15

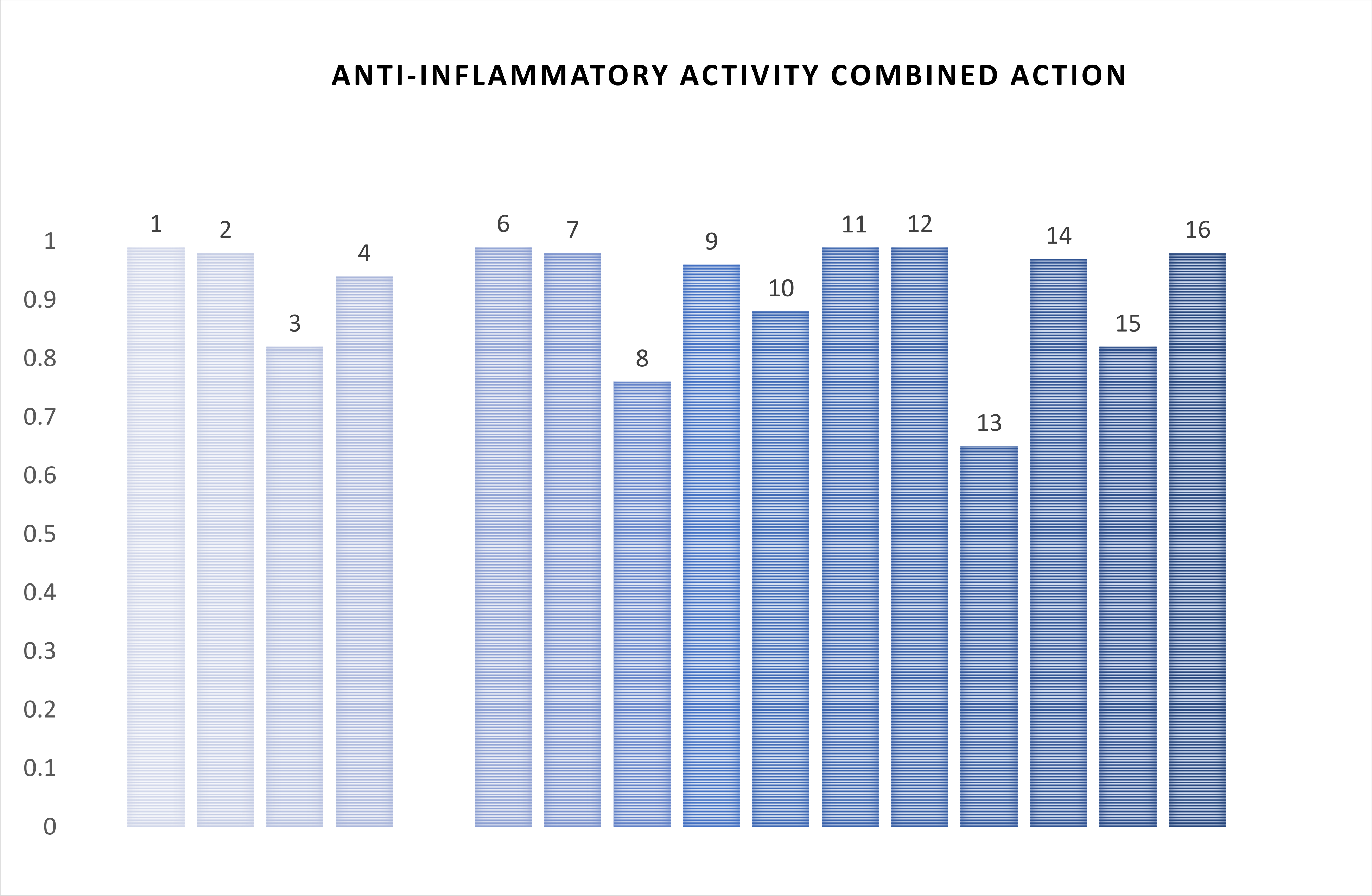
ANTI-INFLAMMATORY ACTIVITY COMBINED ACTION
1
0.9
0.8
0.7
0.6
0.5
0.4
0.3
0.2
0.1
0
1
2
3
4
6
7
8
9
10
11
12
13
14
15
16

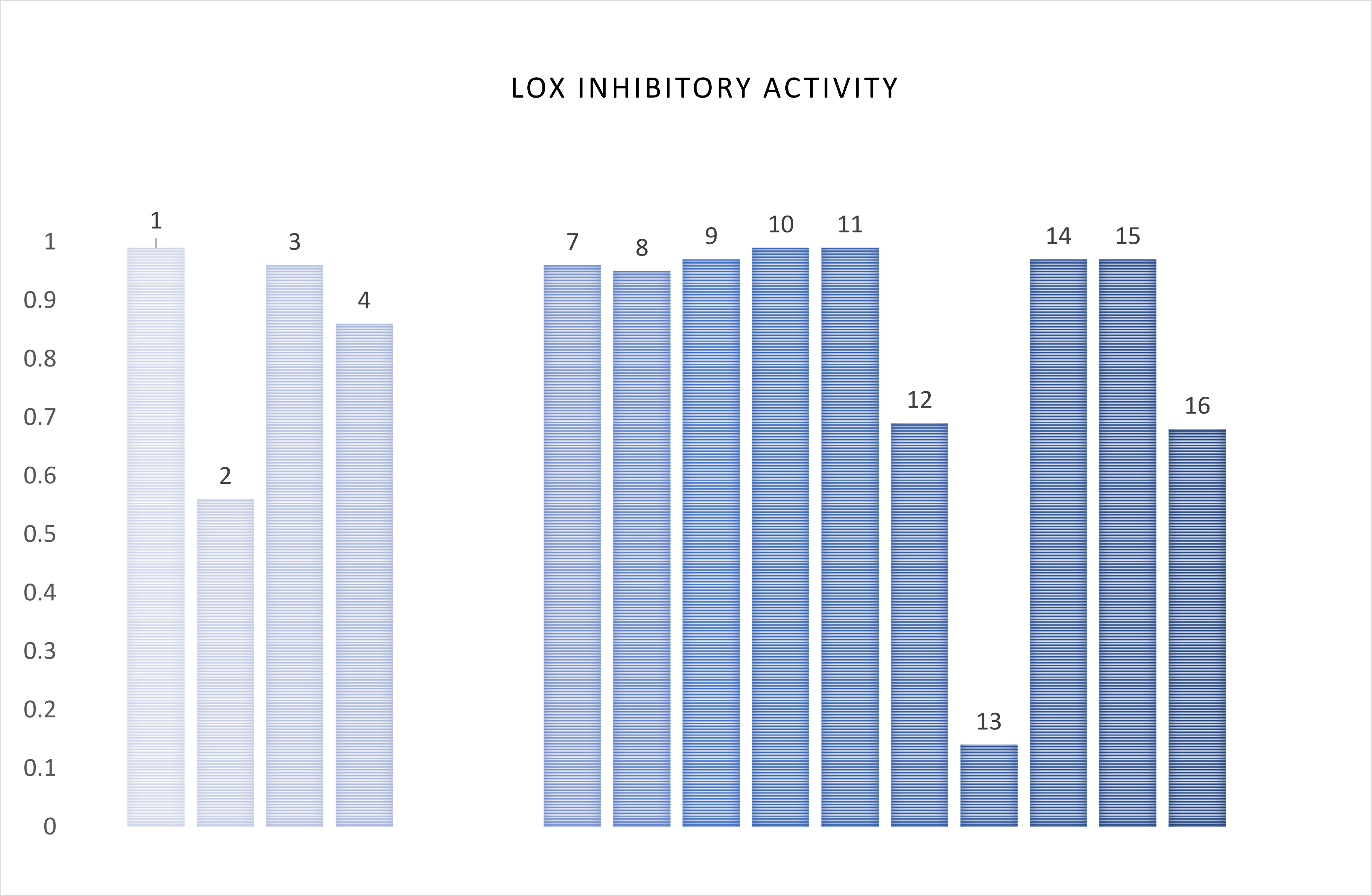
LOX INHIBITORY ACTIVITY
1
0.9
0.8
0.7
0.6
0.5
0.4
0.3
0.2
0.1
0
1
2
3
4
7
8
9
10
11
12
13
14
15
16

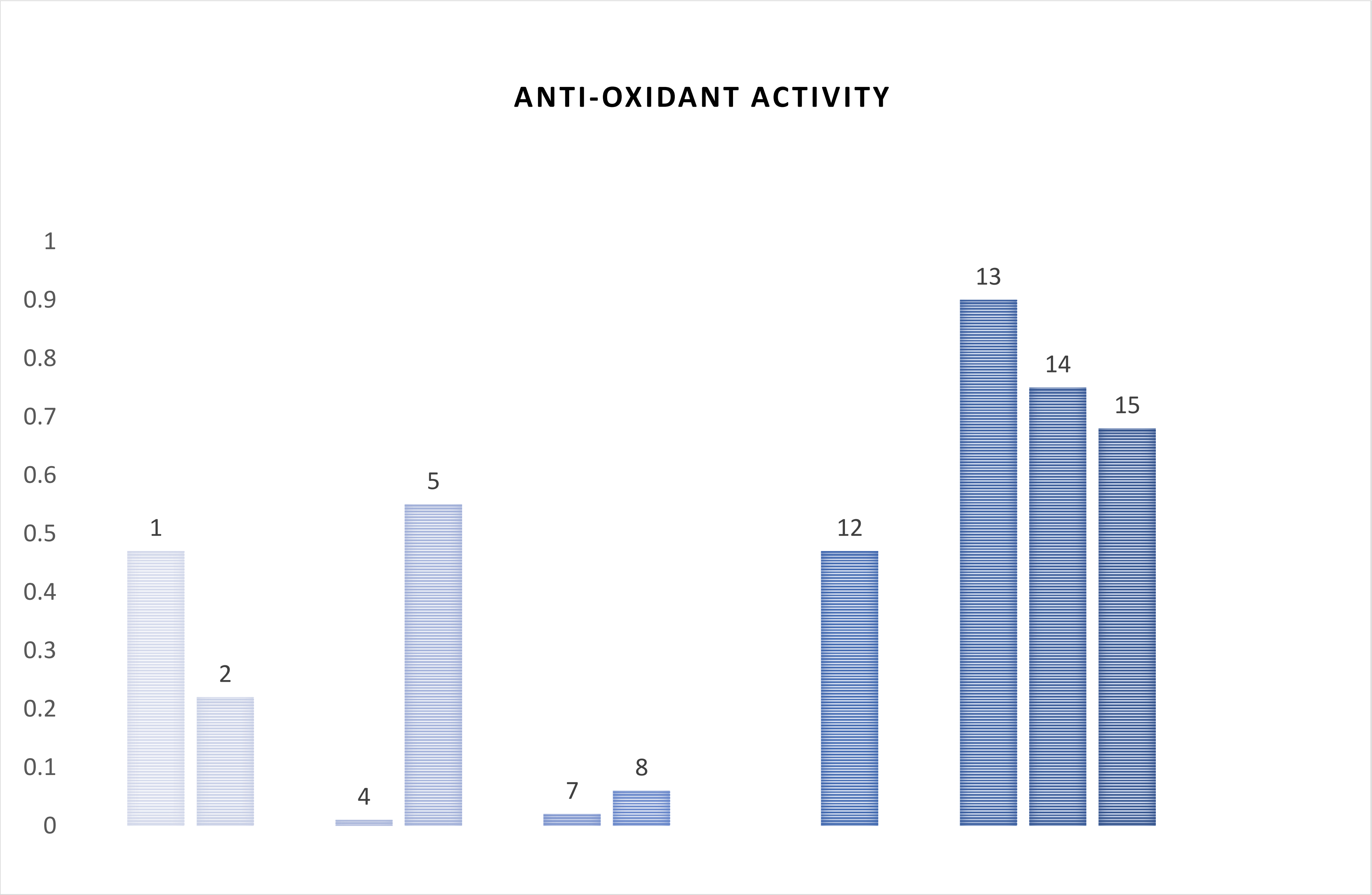
ANTI-OXIDANT ACTIVITY
1
0.9
0.8
0.7
0.6
0.5
0.4
0.3
0.2
0.1
0
1
2
4
5
7
8
12
13
14
15

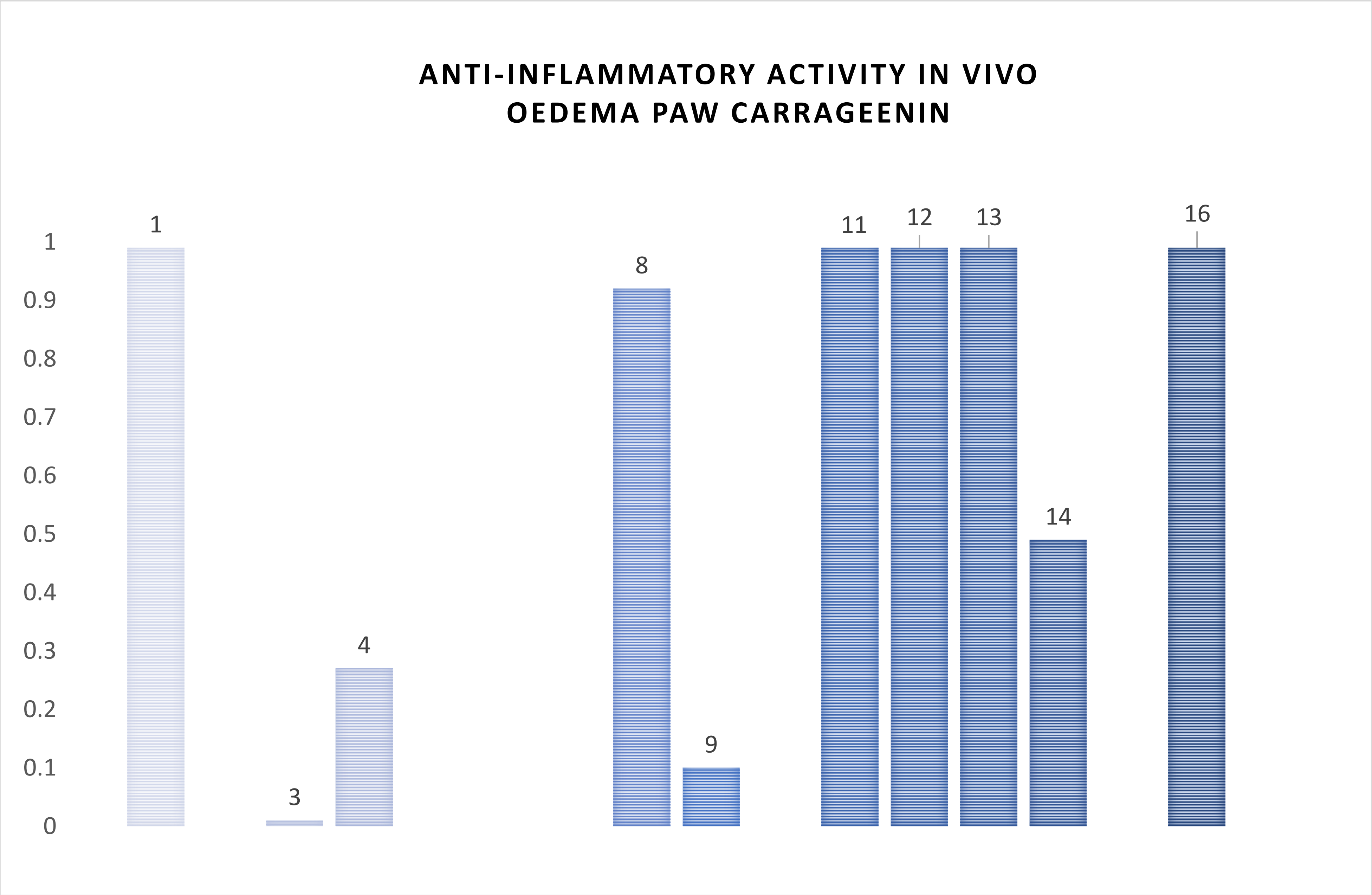
ANTI-INFLAMMATORY ACTIVITY IN VIVO
OEDEMA PAW CARRAGEENIN
1
0.9
0.8
0.7
0.6
0.5
0.4
0.3
0.2
0.1
0
1
3
4
8
9
11
12
13
14
16

2a

COX-1

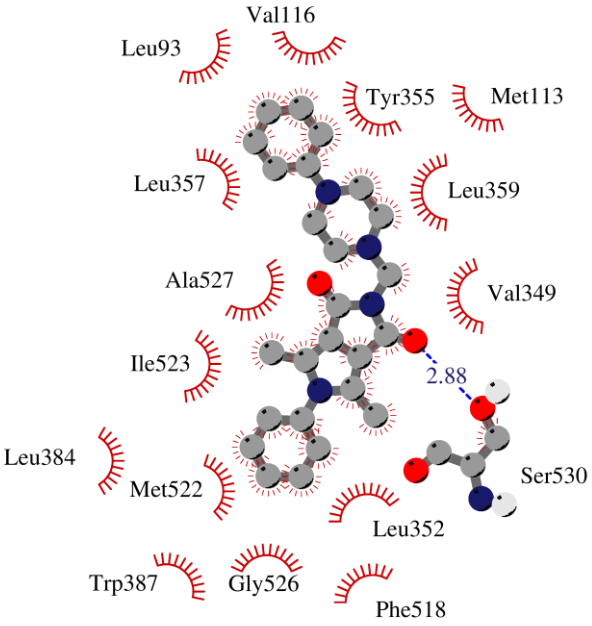

COX-2

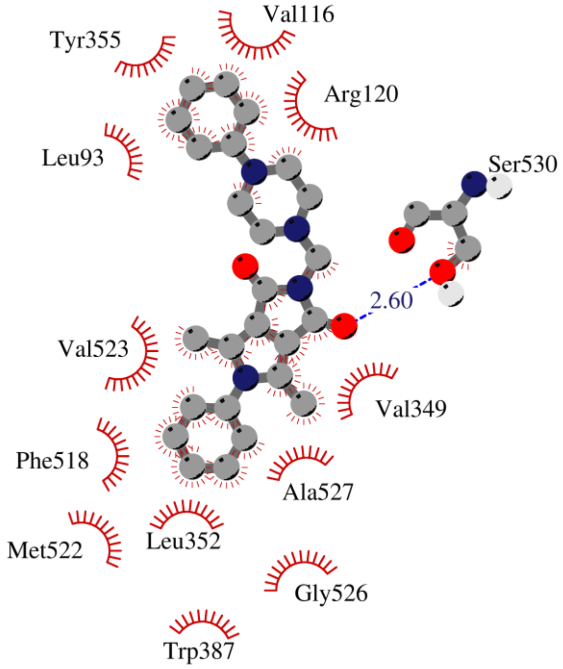

	COX-1	COX-2
Free enthalpy of binding [kcal/mol]	-9.33	-10.39
Inhibition constant [nM]	3900	24.24

COX-1

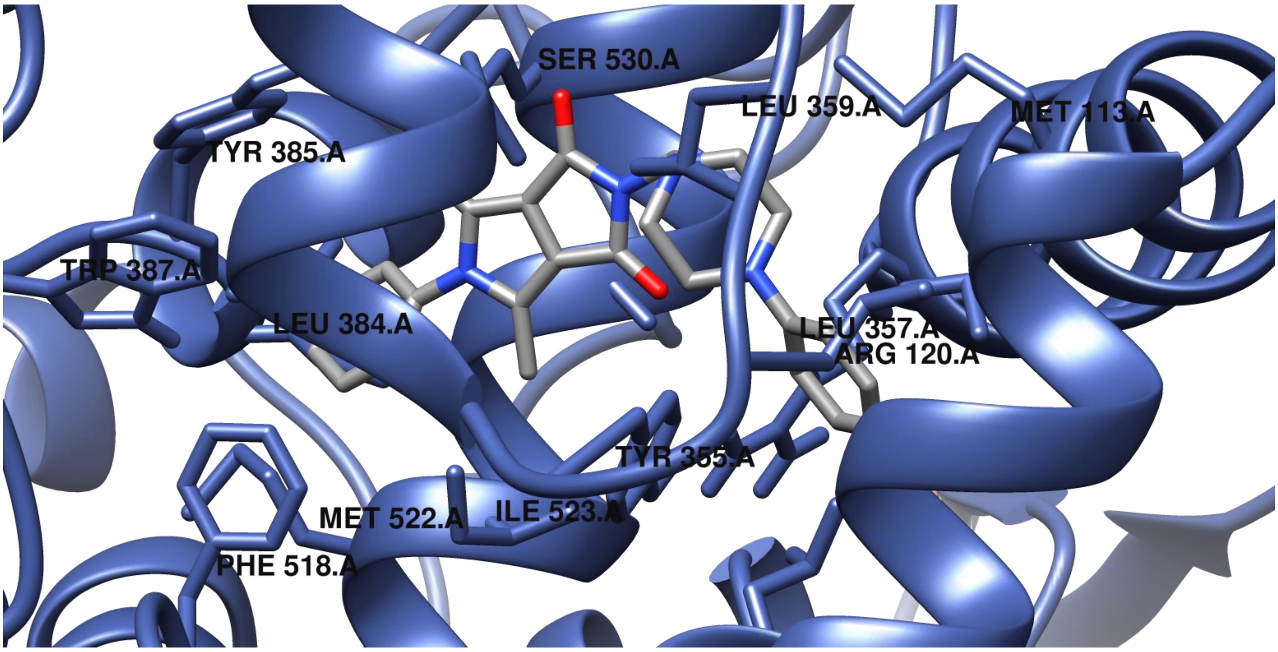
SER 530.A
LEU 359.A
MET 113.A
TYR 385.A
TRP 387.A
LEU 384.A
LEU 357.A
ARG 120.A
TYR 355.A
MET 522.A
ILE 523.A
PHE 518.A

COX-2

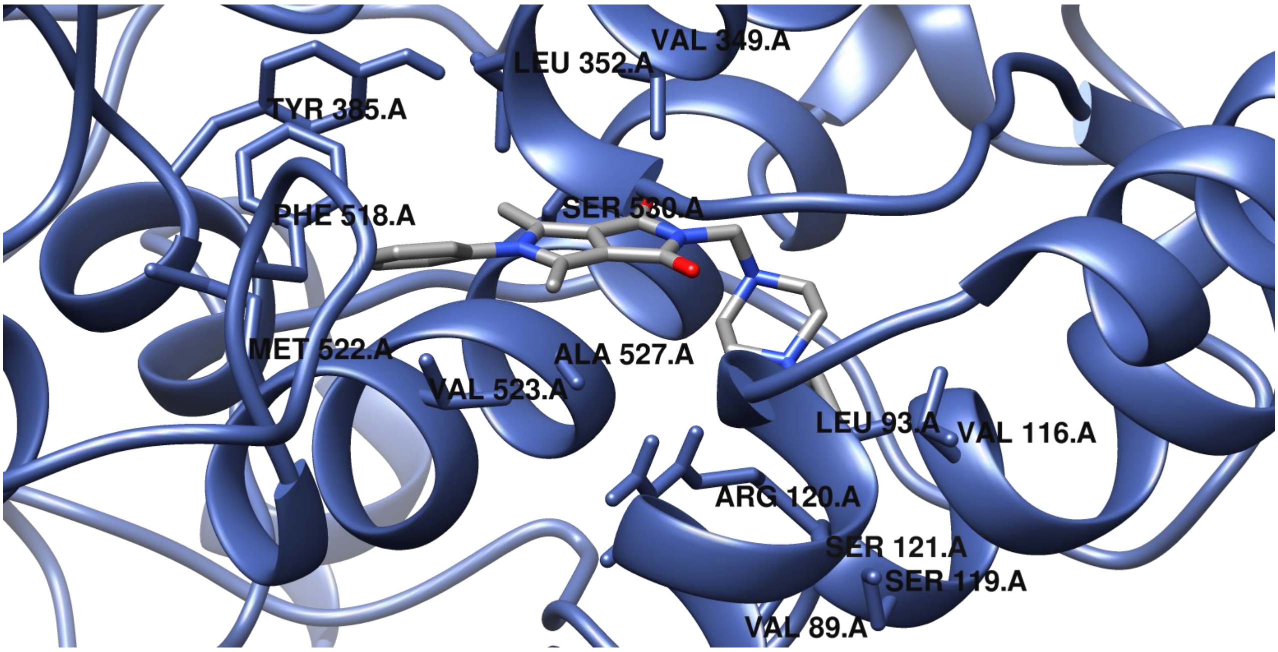
VAL 349.A
LEU 352.A
TYR 385.A
PHE 518.A
SER 530.A
MET 522.A
ALA 527.A
VAL 523.A
LEU 93.A
VAL 116.A
ARG 120.A
SER 121.A
SER 119.A
VAL 89.A

2b

COX-1	COX-2
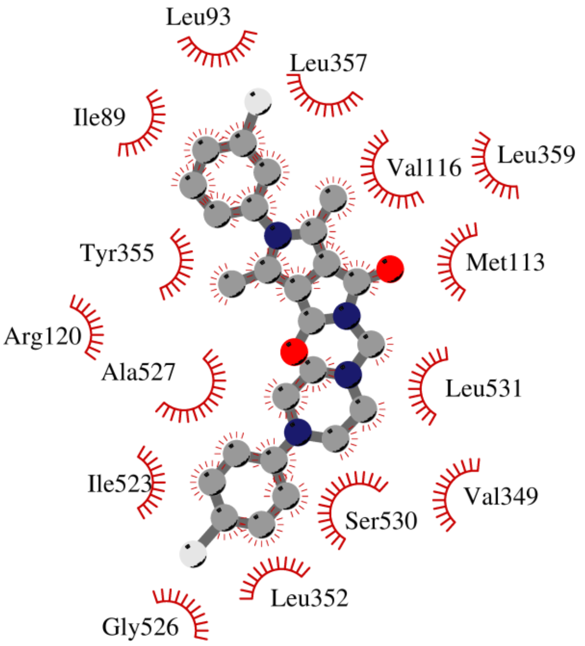	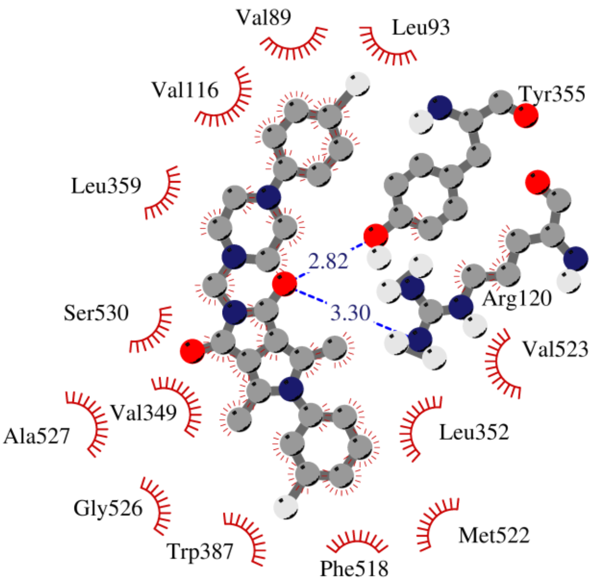

	COX-1	COX-2
Free enthalpy of binding [kcal/mol]	-7.43	-10.95
Inhibition constant [nM]	3590	9.84

COX-1

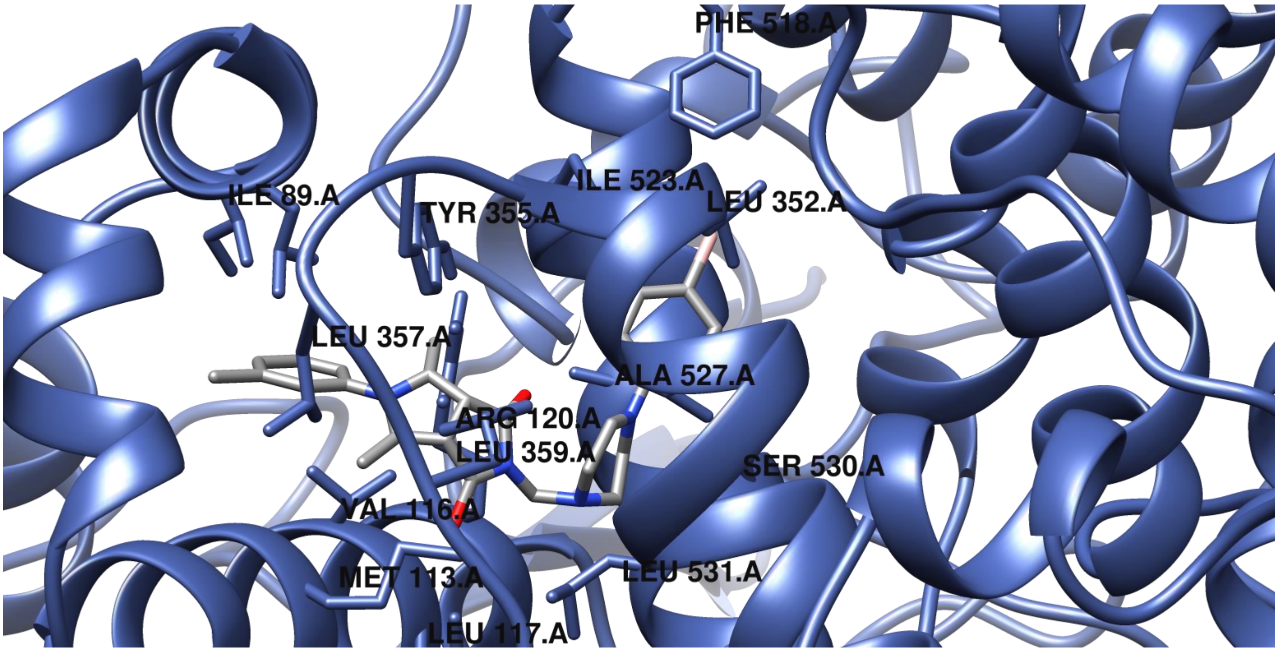
PHE 518.A
ILE 89.A
TYR 355.A
ILE 523.A
LEU 352.A
LEU 357.A
ALA 527.A
ARG 120.A
LEU 359.A
SER 530.A
VAL 116.A
MET 113.A
LEU 531.A
LEU 117.A

COX-2

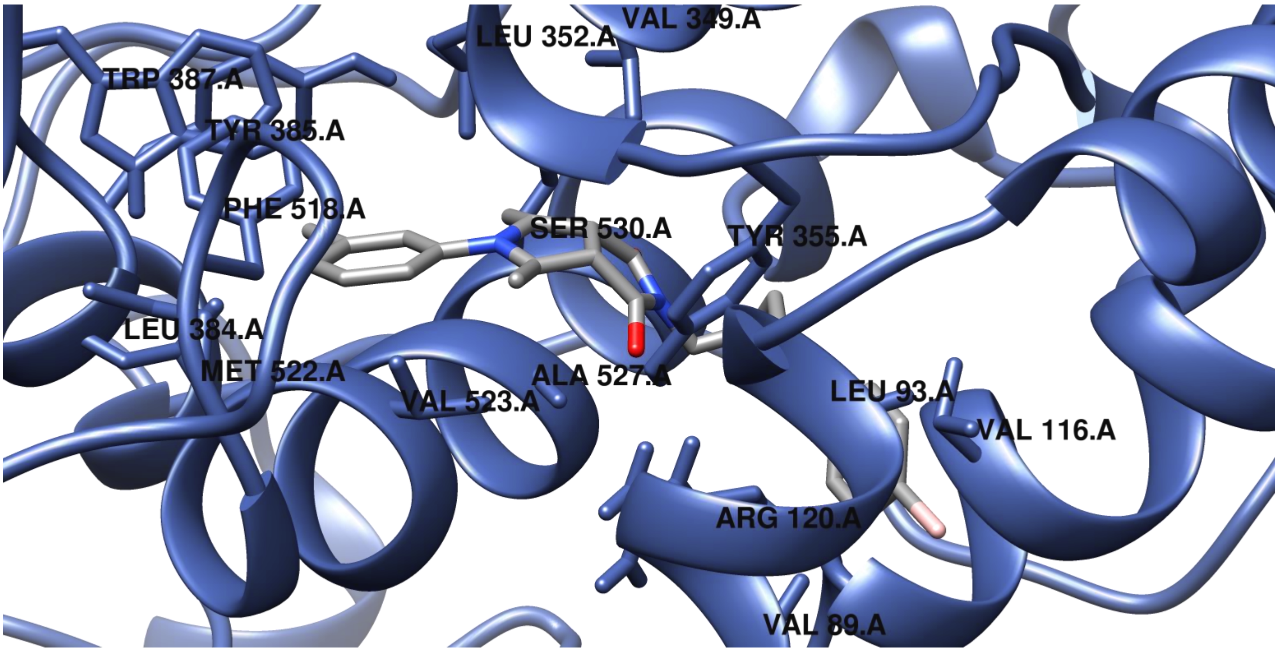
LEU 352.A
VAL 349.A
TRP 387.A
TYR 385.A
PHE 518.A
SER 530.A
TYR 355.A
LEU 384.A
MET 522.A
ALA 527.A
VAL 523.A
LEU 93.A
VAL 116.A
ARG 120.A
VAL 89.A

2c	
**COX-1**	**COX-2**
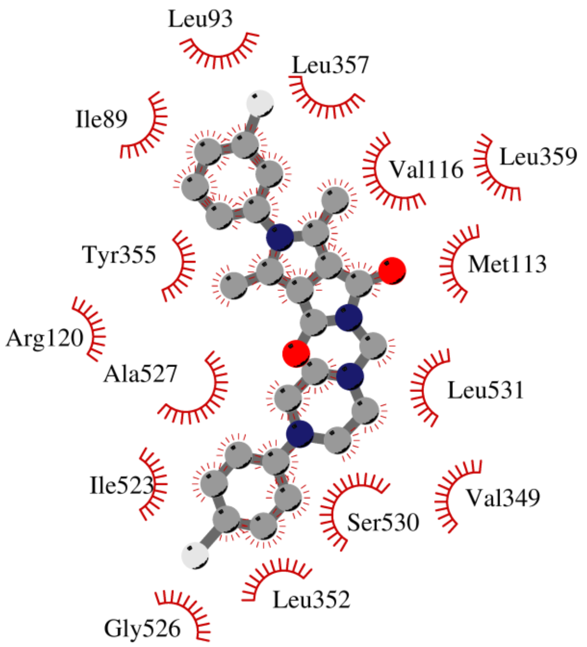	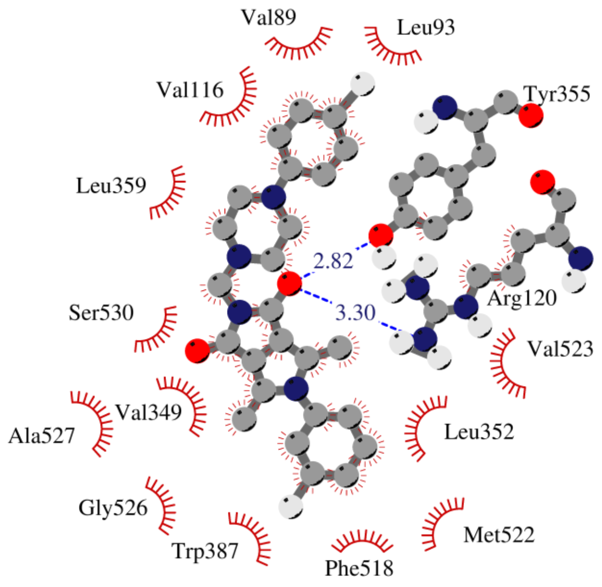

	COX-1	COX-2
Free enthalpy of binding [kcal/mol]	-9.01	-10.84
Inhibition constant [nM]	250	11.3

COX-1

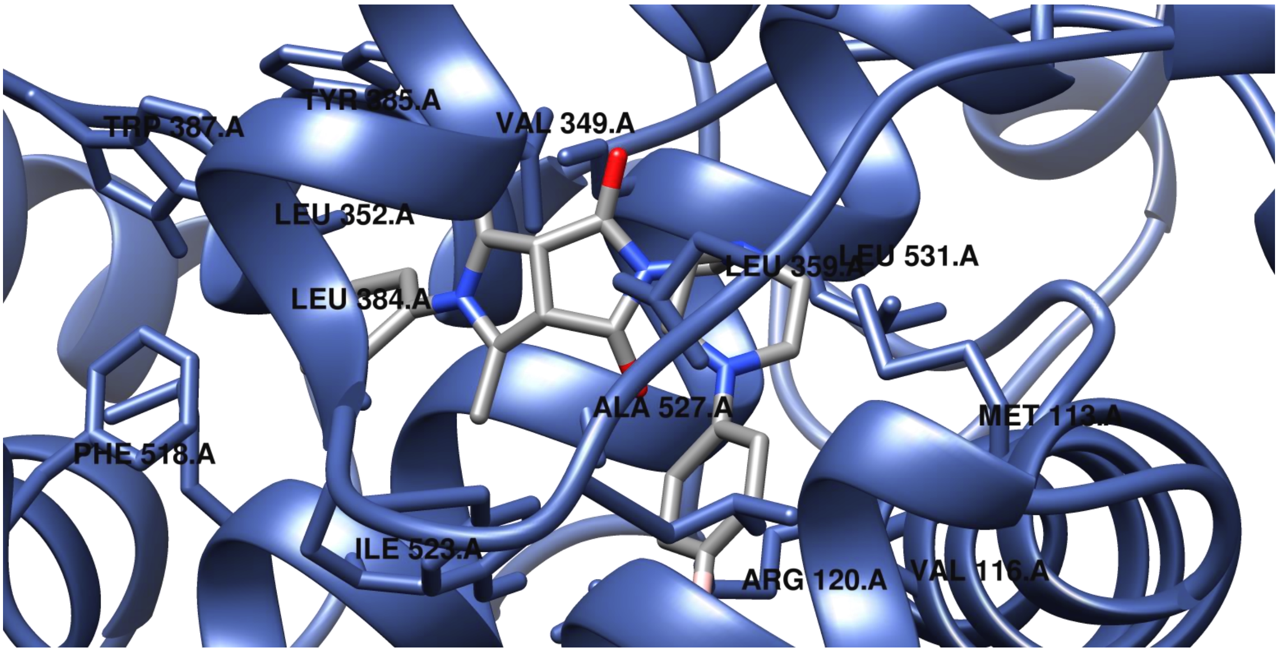
TRP 387.A
TYR 385.A
VAL 349.A
LEU 352.A
LEU 359.A
LEU 531.A
LEU 384.A
ALA 527.A
MET 113.A
PHE 518.A
ILE 523.A
ARG 120.A
VAL 116.A

COX-2

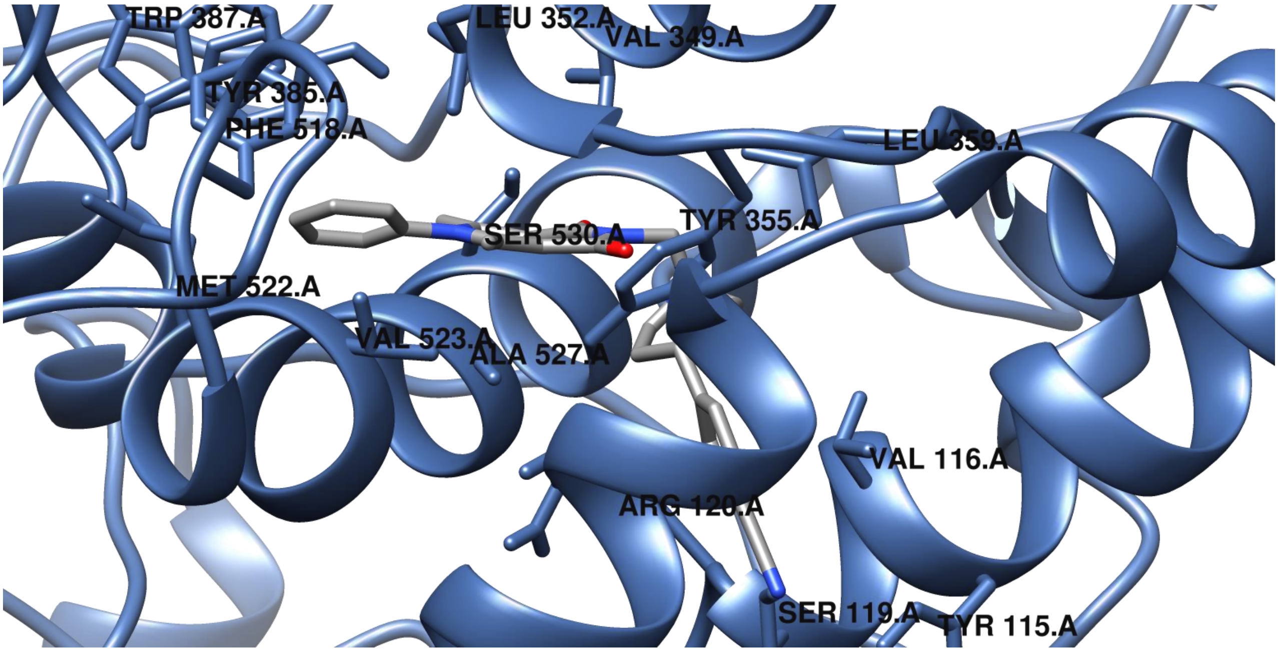
TRP 387.A
LEU 352.A
VAL 349.A
TYR 385.A
PHE 518.A
LEU 359.A
SER 530.A
TYR 355.A
MET 522.A
VAL 523.A
ALA 527.A
VAL 116.A
ARG 120.A
SER 119.A
TYR 115.A

2d

COX-1 **COX-2**

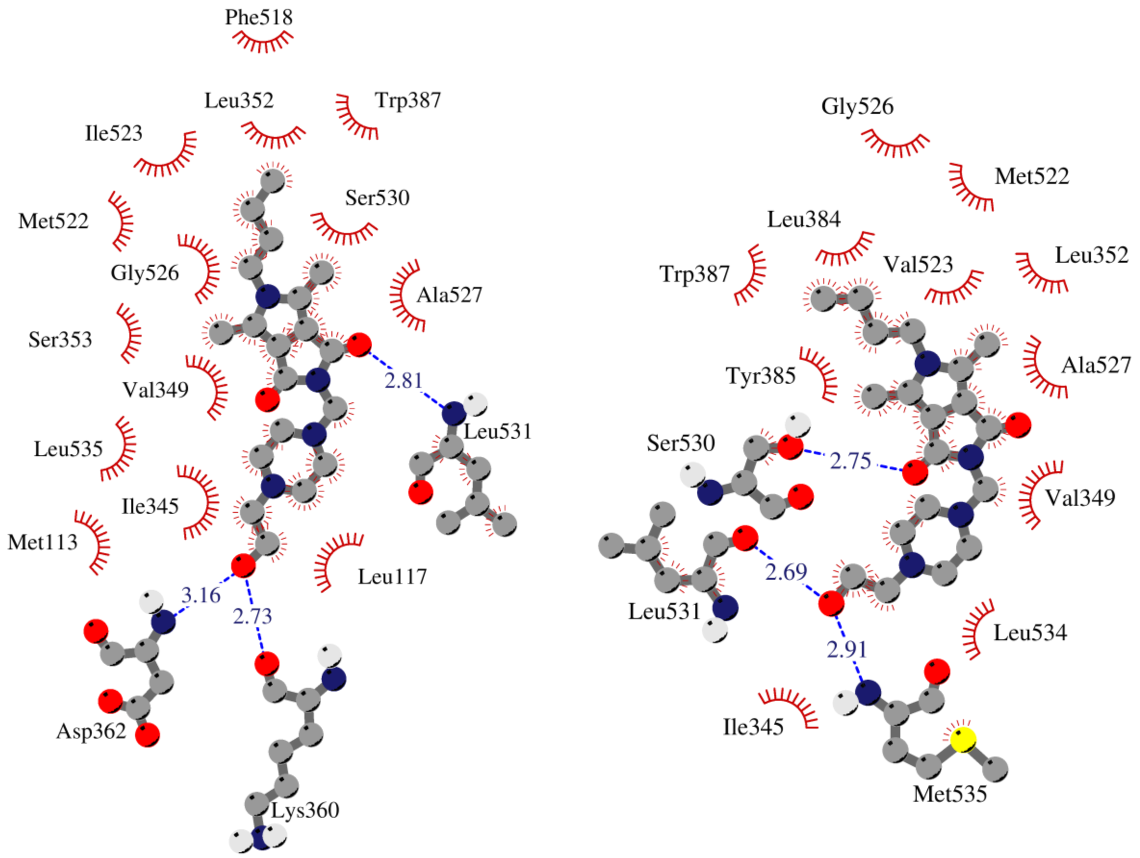

	COX-1	COX-2
Free enthalpy of binding [kcal/mol]	-8.29	-8.78
Inhibition constant [nM]	835.53	369.44

COX-1
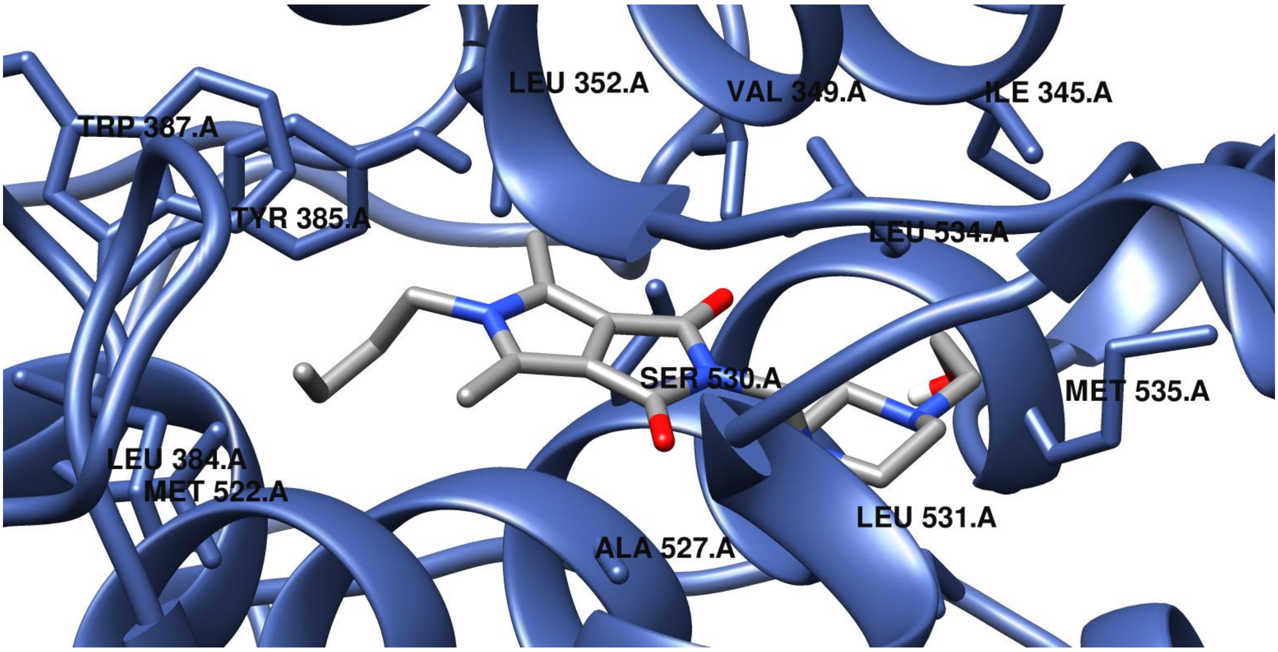LEU 352.A<br>VAL 349.A<br>ILE 345.A<br>TRP 387.A<br>TYR 385.A<br>LEU 534.A<br>SER 530.A<br>MET 535.A<br>LEU 384.A<br>MET 522.A<br>LEU 531.A<br>ALA 527.A
**COX-2**
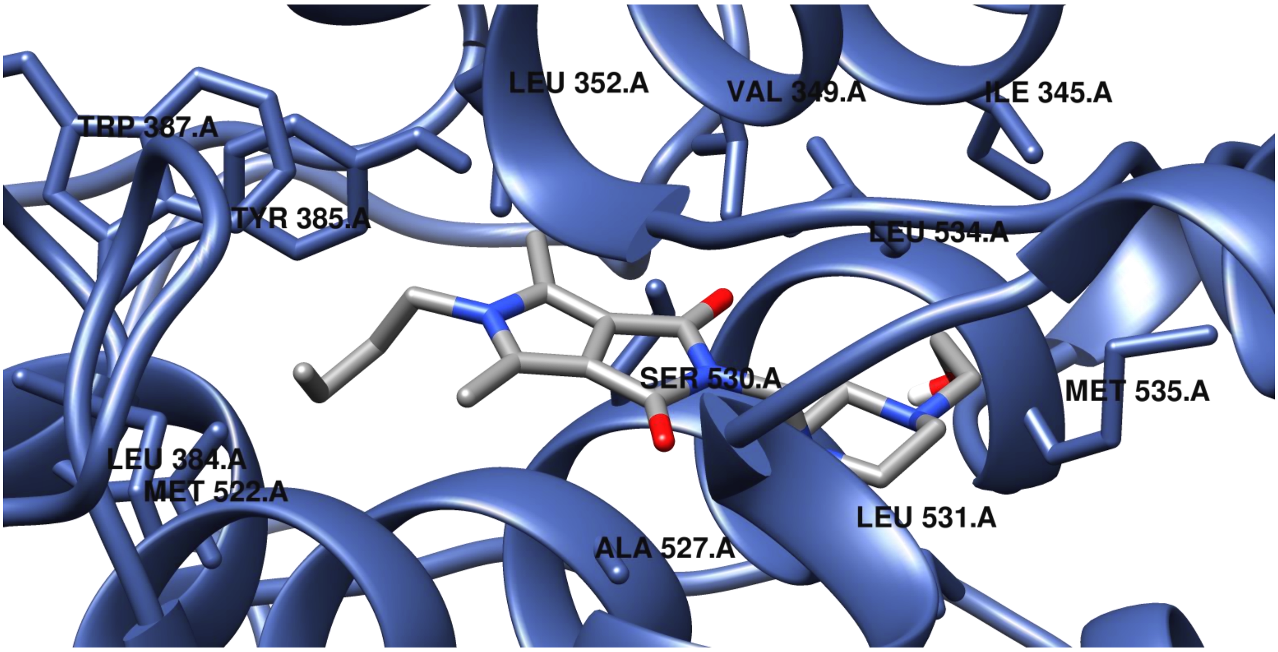LEU 352.A<br>VAL 349.A<br>ILE 345.A<br>TRP 387.A<br>TYR 385.A<br>LEU 534.A<br>SER 530.A<br>MET 535.A<br>LEU 384.A<br>MET 522.A<br>LEU 531.A<br>ALA 527.A

2e

COX-1	COX-2
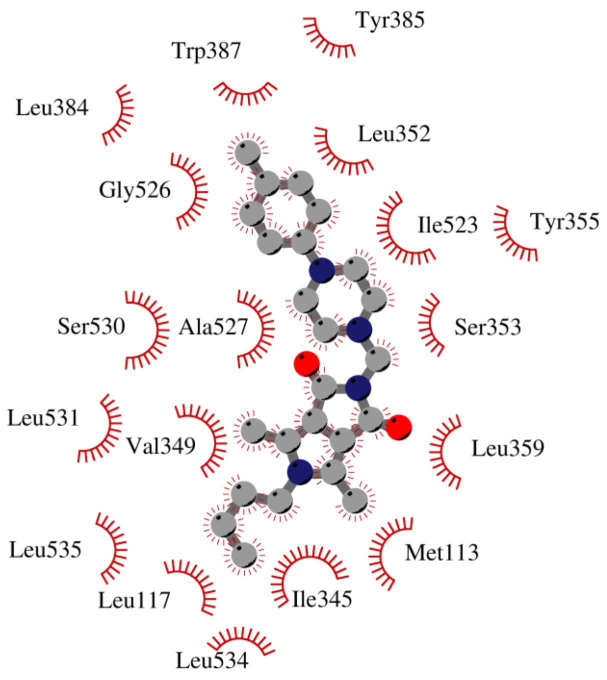	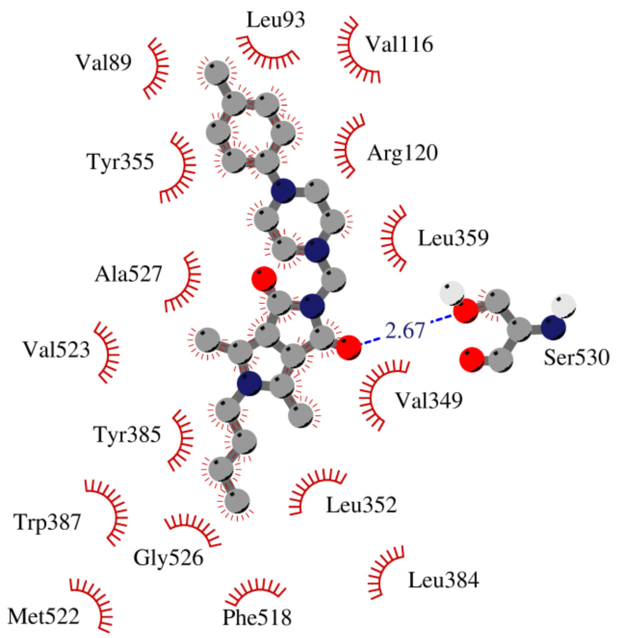

	COX-1	COX-2
Free enthalpy of binding [kcal/mol]	-9.32	-9.74
Inhibition constant [nM]	147	72

COX-1

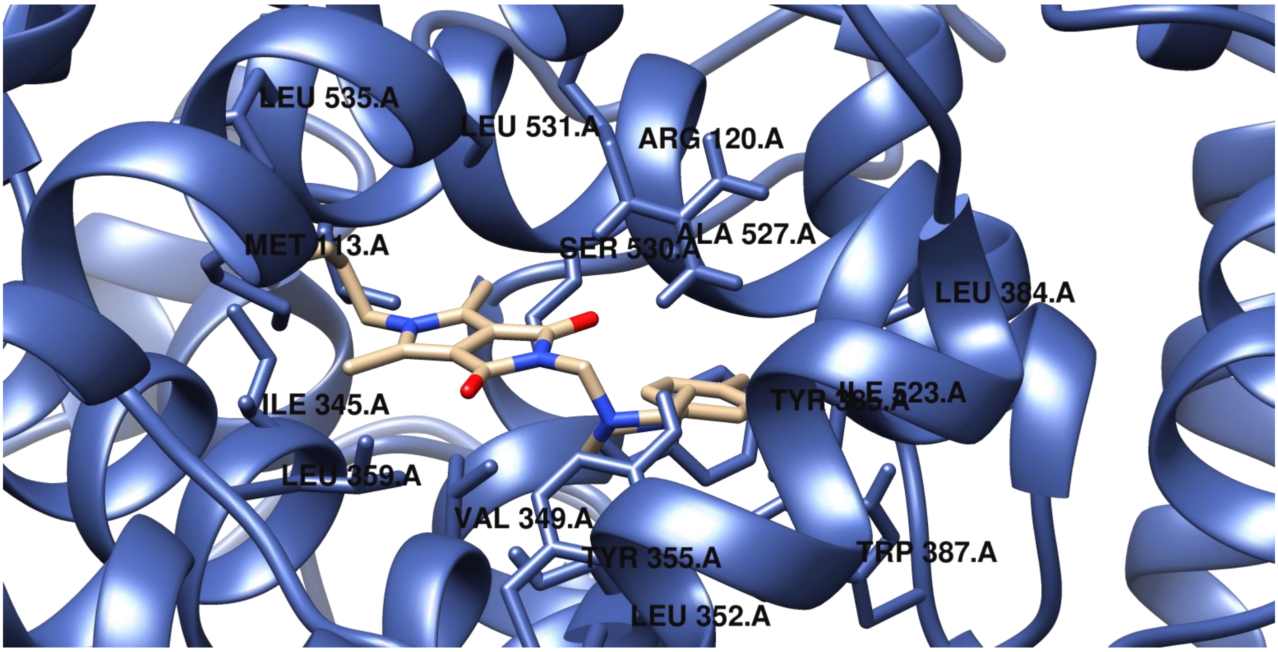
LEU 535.A
LEU 531.A
ARG 120.A
MET 113.A
SER 530.A
ALA 527.A
LEU 384.A
ILE 345.A
TYR 385.A
ILE 523.A
LEU 359.A
VAL 349.A
TYR 355.A
TRP 387.A
LEU 352.A

COX-2

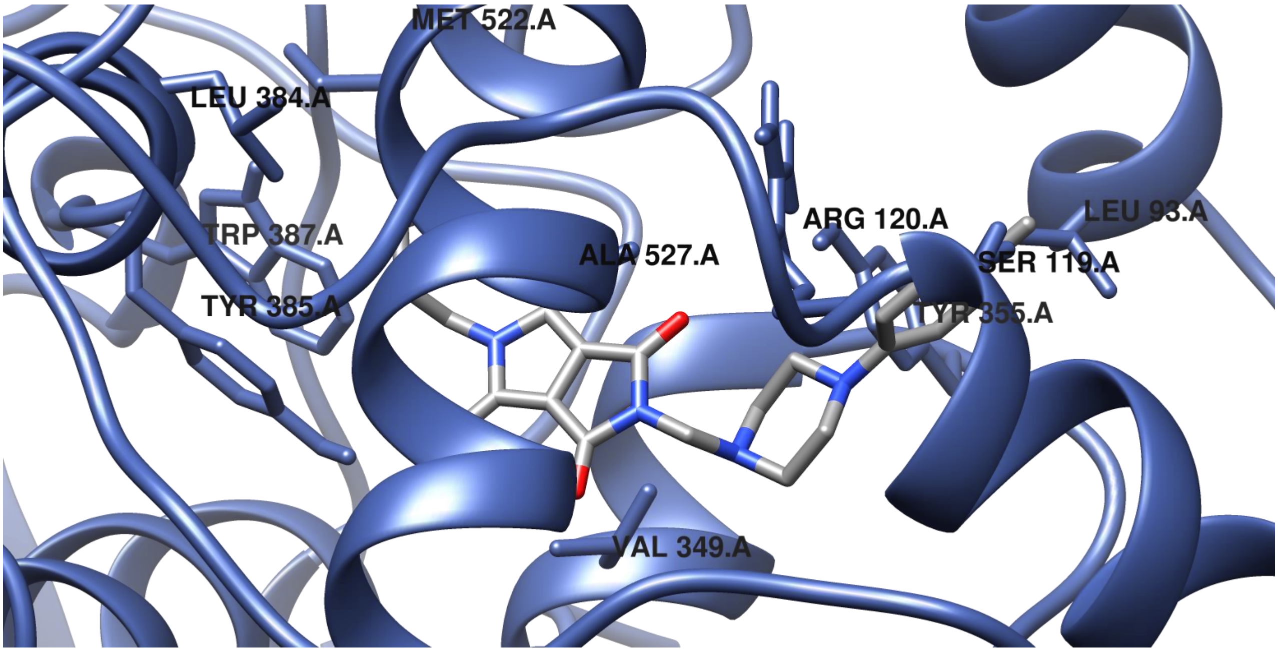
MET 522.A
LEU 384.A
TRP 387.A
TYR 385.A
ALA 527.A
ARG 120.A
LEU 93.A
SER 119.A
TYR 355.A
VAL 349.A

2f

COX-1

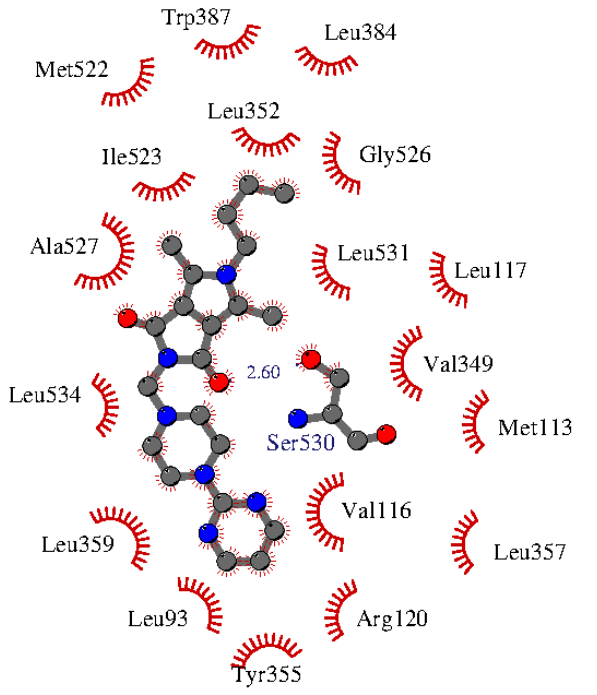

COX-2

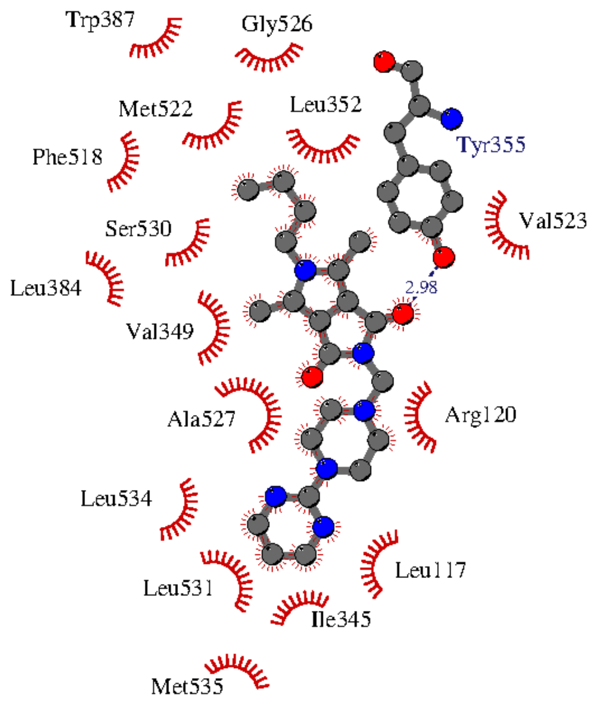

	COX-1	COX-2
Free enthalpy of binding [kcal/mol]	-9.21	-11.26
Inhibition constant [nM]	177.49	5.57

COX-1

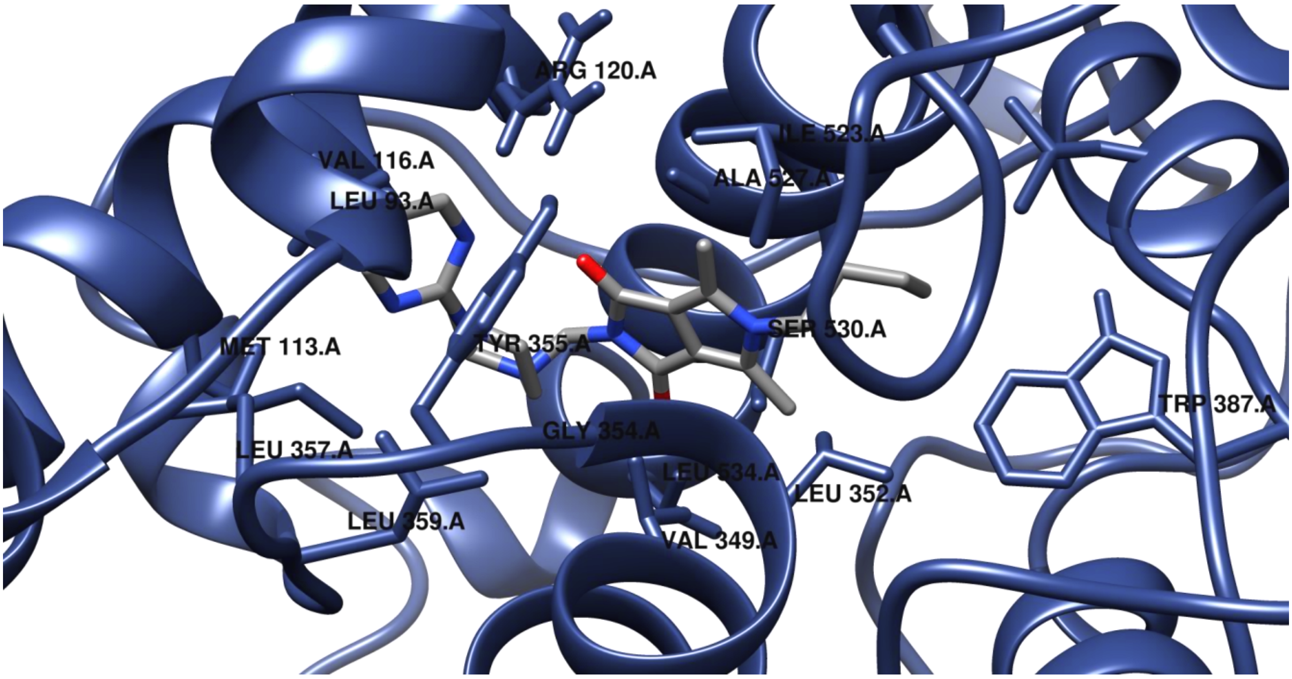
ARG 120.A
ILE 523.A
VAL 116.A
ALA 527.A
LEU 93.A
SER 530.A
MET 113.A
TYR 355.A
TRP 387.A
GLY 354.A
LEU 357.A
LEU 534.A
LEU 352.A
LEU 359.A
VAL 349.A

COX-2

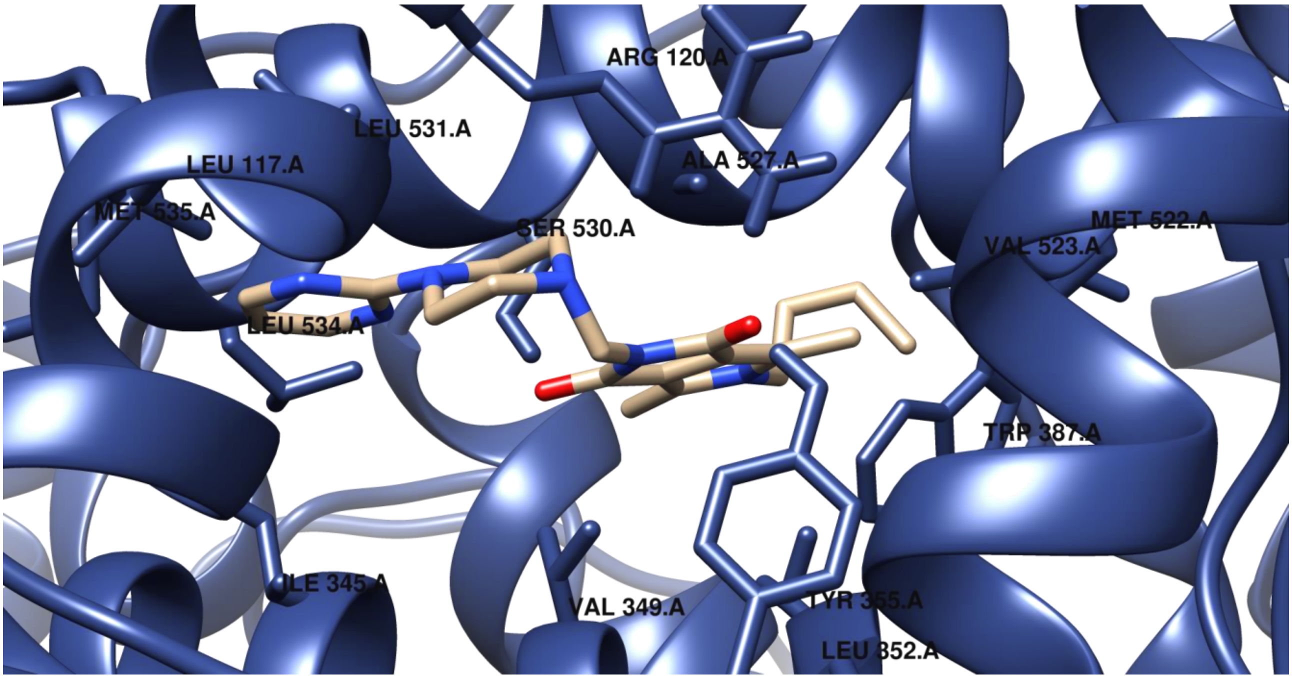
ARG 120.A
LEU 531.A
LEU 117.A
ALA 527.A
MET 535.A
SER 530.A
MET 522.A
VAL 523.A
LEU 534.A
TRP 387.A
ILE 345.A
VAL 349.A
TYR 355.A
LEU 352.A

2g

**COX-1**	**COX-2**

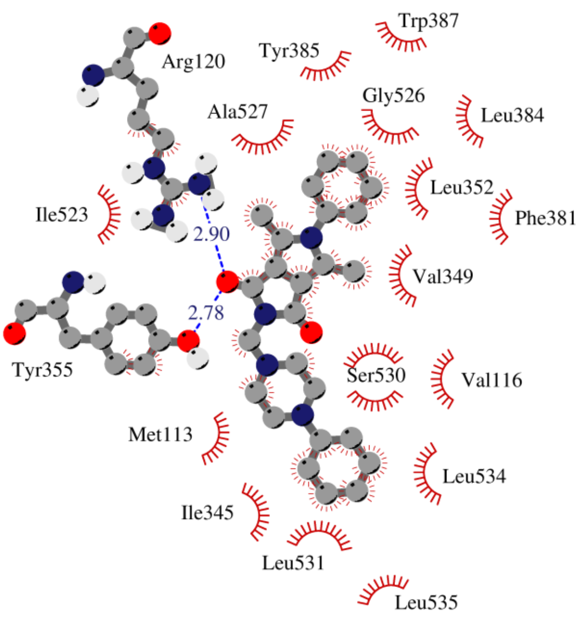

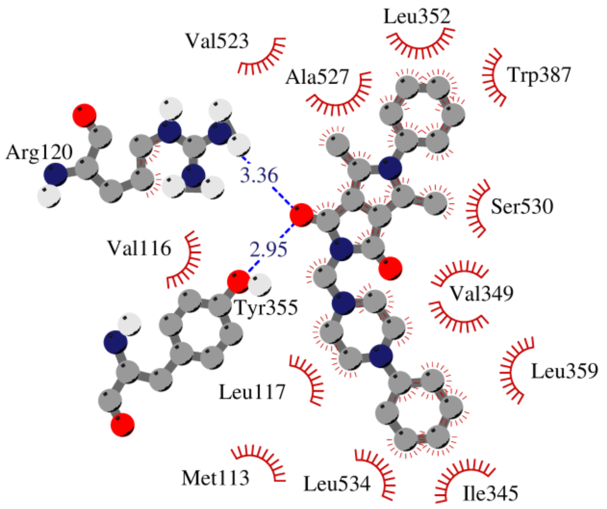

	COX-1	COX-2
Free enthalpy of binding [kcal/mol]	-9.75	-10.98
Inhibition constant [nM]	70.95	8.94

COX-1

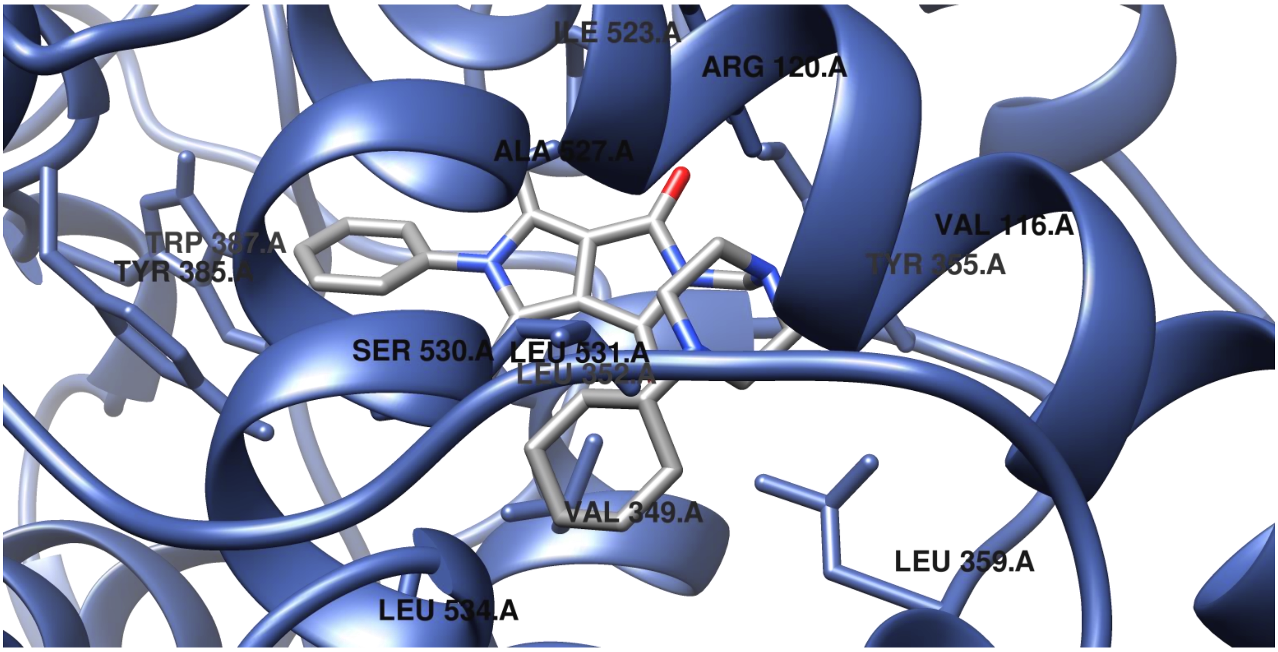
ILE 523.A
ARG 120.A
ALA 527.A
TRP 387.A
TYR 385.A
VAL 116.A
TYR 355.A
SER 530.A
LEU 531.A
LEU 352.A
VAL 349.A
LEU 359.A
LEU 534.A

COX-2

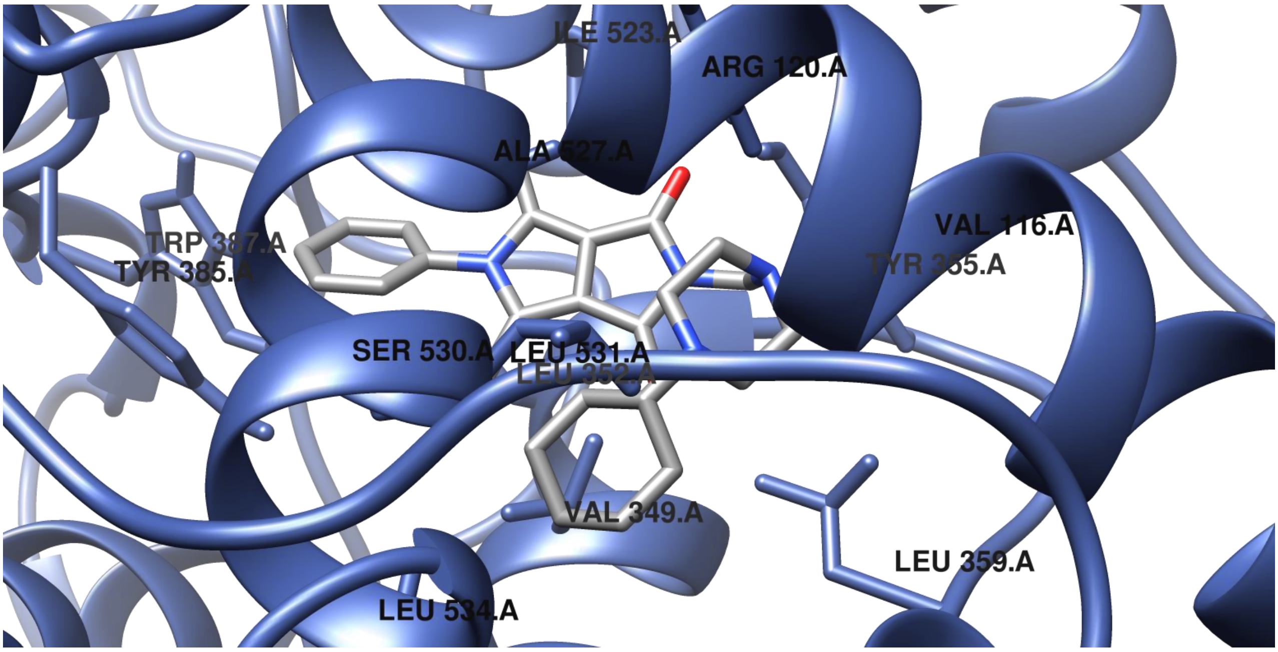
ILE 523.A
ARG 120.A
ALA 527.A
TRP 387.A
TYR 385.A
VAL 116.A
TYR 355.A
SER 530.A
LEU 531.A
LEU 352.A
VAL 349.A
LEU 359.A
LEU 534.A

2h

COX-1

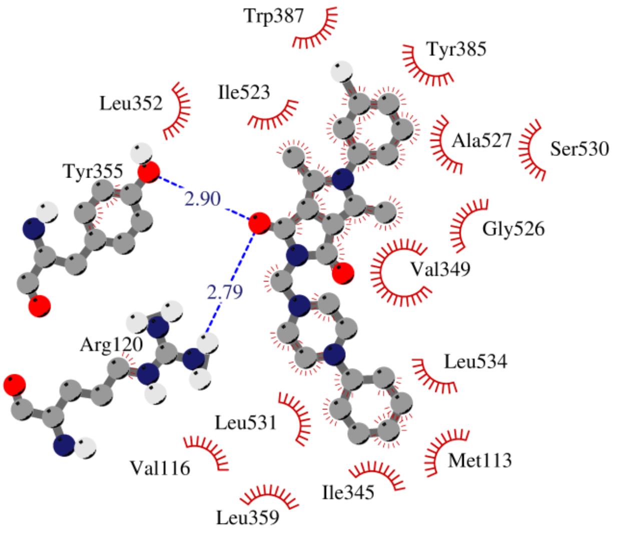

COX-2

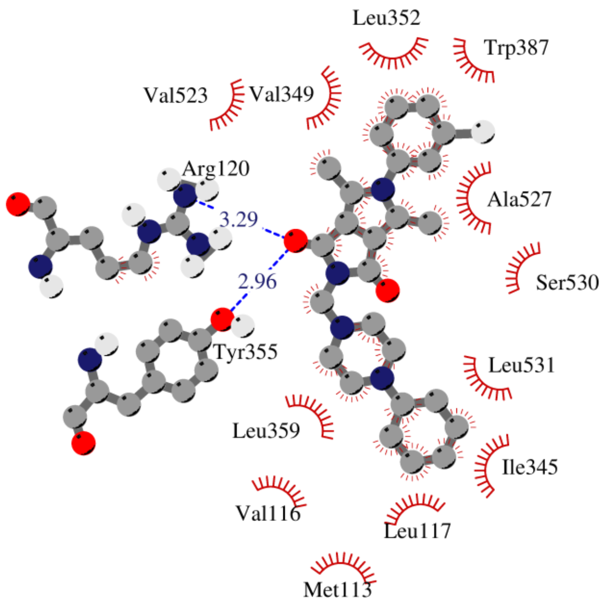

	COX-1	COX-2
Free enthalpy of binding [kcal/mol]	-10.53	-11.92
Inhibition constant [nM]	19.11	1.84

COX-1
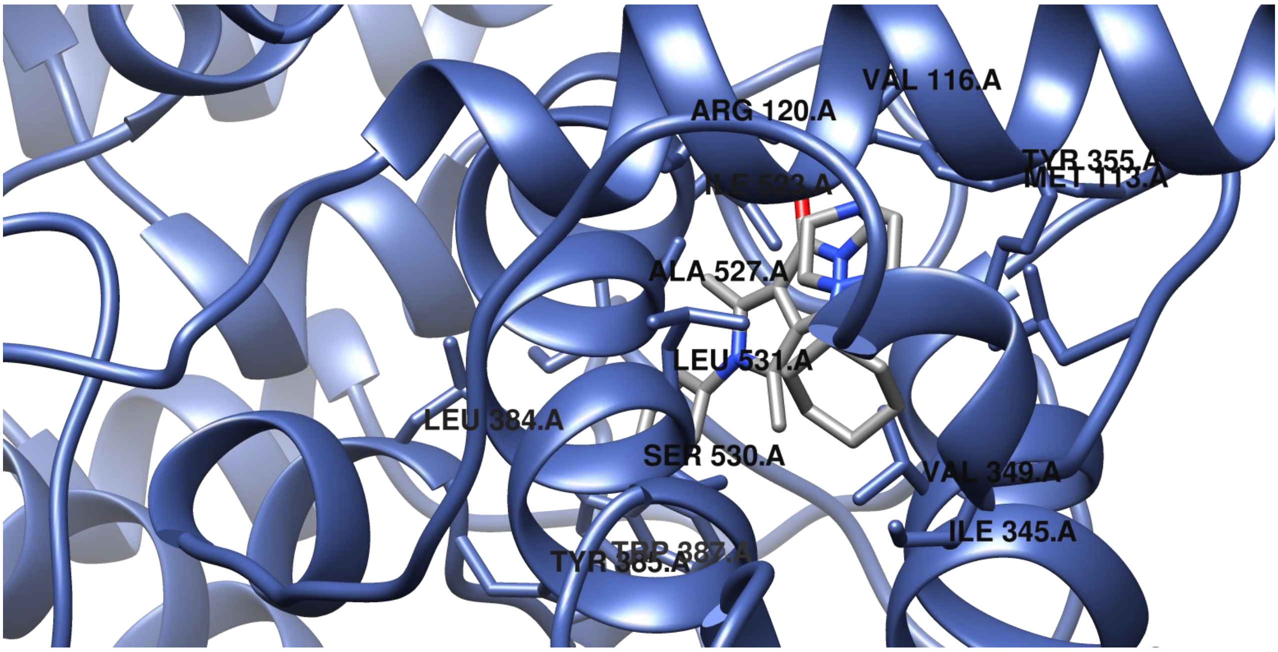

VAL 116.A
ARG 120.A
TYR 355.A
MET 113.A
ILE 523.A
ALA 527.A
LEU 531.A
LEU 384.A
SER 530.A
VAL 349.A
ILE 345.A
TRP 387.A
TYR 385.A

COX-2
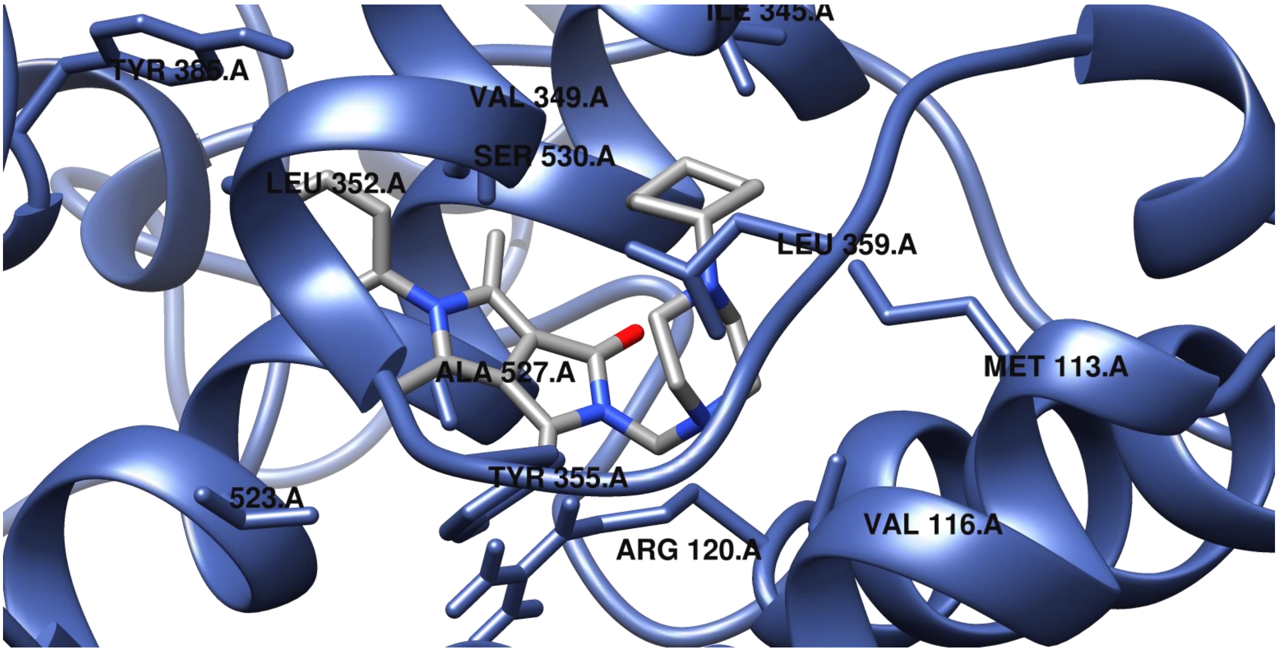

ILE 345.A
TYR 385.A
VAL 349.A
SER 530.A
LEU 352.A
LEU 359.A
MET 113.A
ALA 527.A
523.A
TYR 355.A
VAL 116.A
ARG 120.A

2i

COX-1

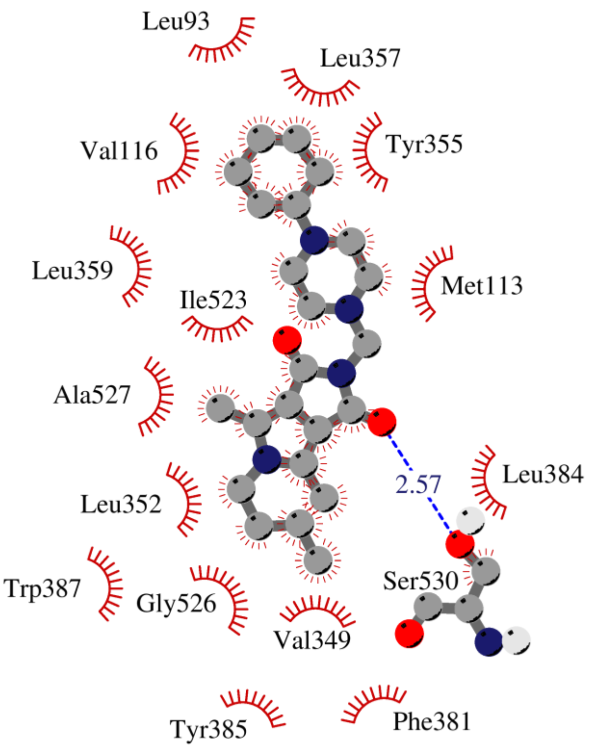

COX-2

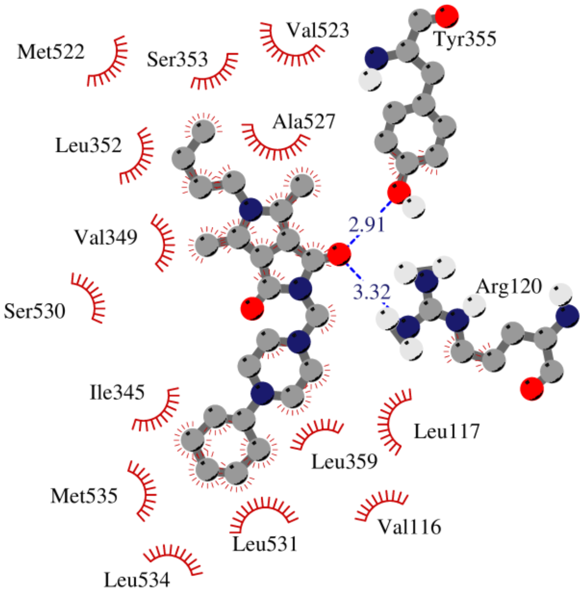

	COX-1	COX-2
Free enthalpy of binding [kcal/mol]	-9.05	-10.46
Inhibition constant [nM]	233.24	21.67

COX-1

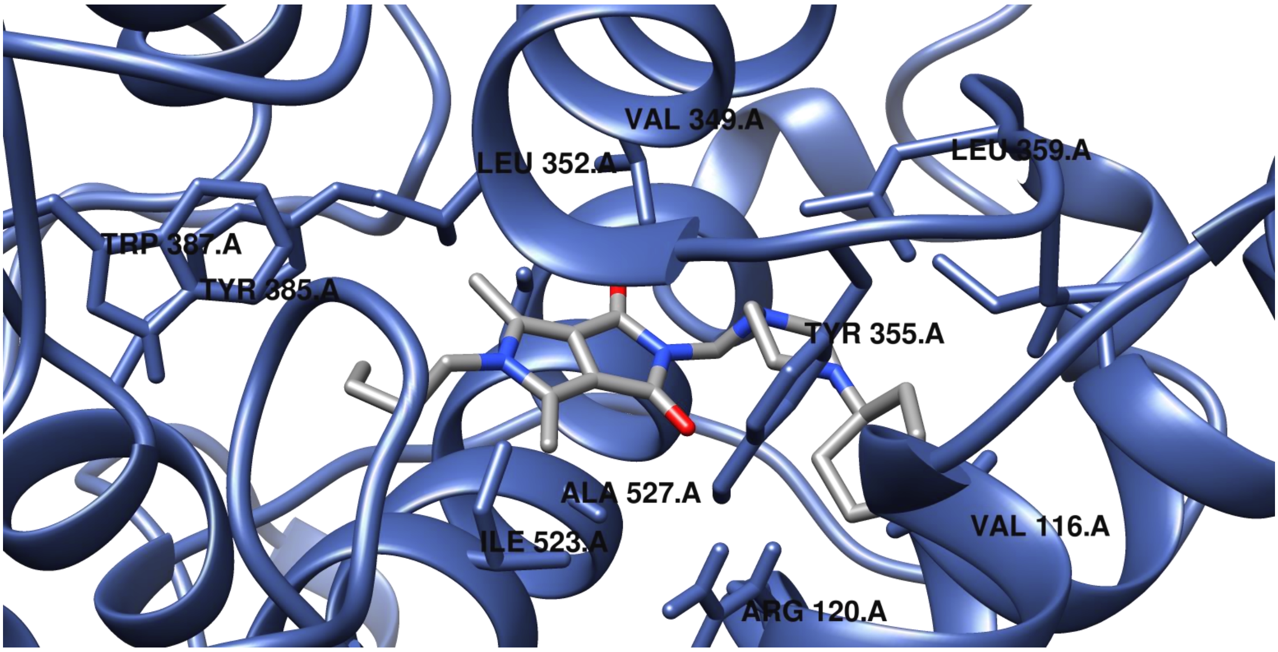
VAL 349.A
LEU 352.A
LEU 359.A
TRP 387.A
TYR 385.A
TYR 355.A
ALA 527.A
ILE 523.A
VAL 116.A
ARG 120.A

COX-2

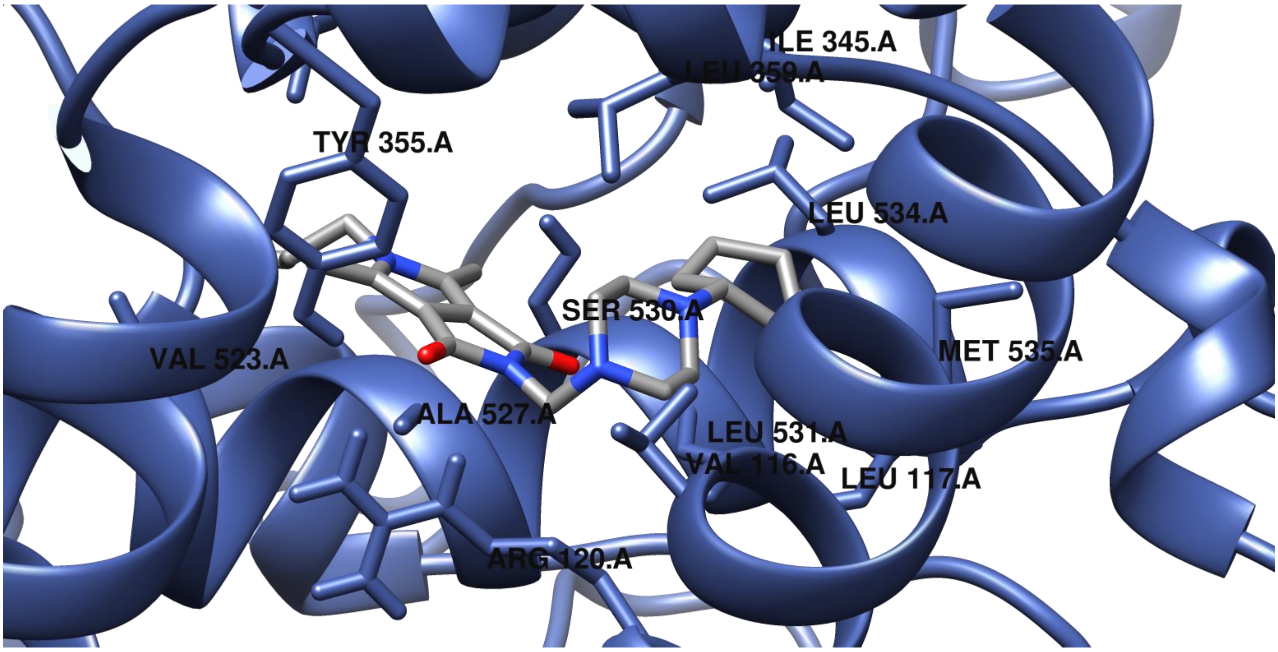
ILE 345.A
LEU 359.A
TYR 355.A
LEU 534.A
SER 530.A
VAL 523.A
MET 535.A
ALA 527.A
LEU 531.A
VAL 116.A
LEU 117.A
ARG 120.A

2j

COX-1	COX-2
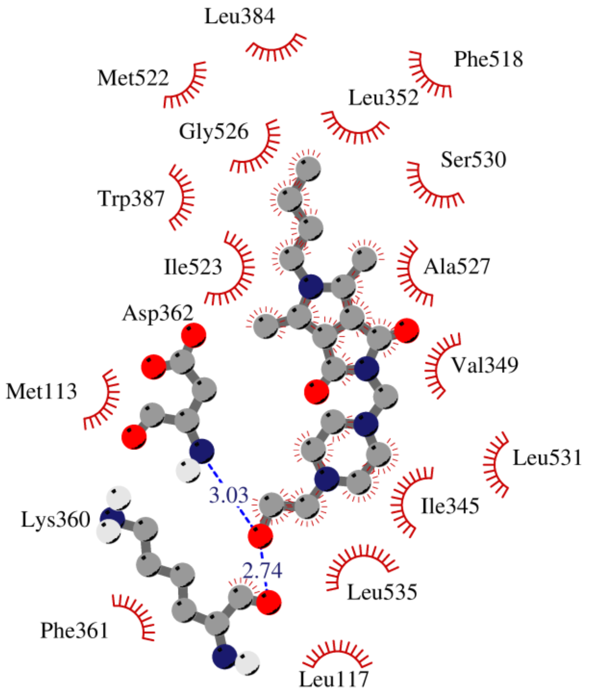	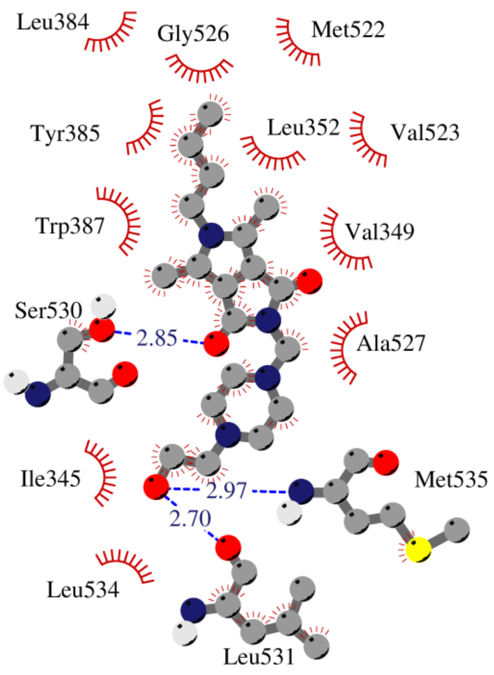

	COX-1	COX-2
Free enthalpy of binding [kcal/mol]	-8.89	-8.88
Inhibition constant [nM]	30	309

COX-1
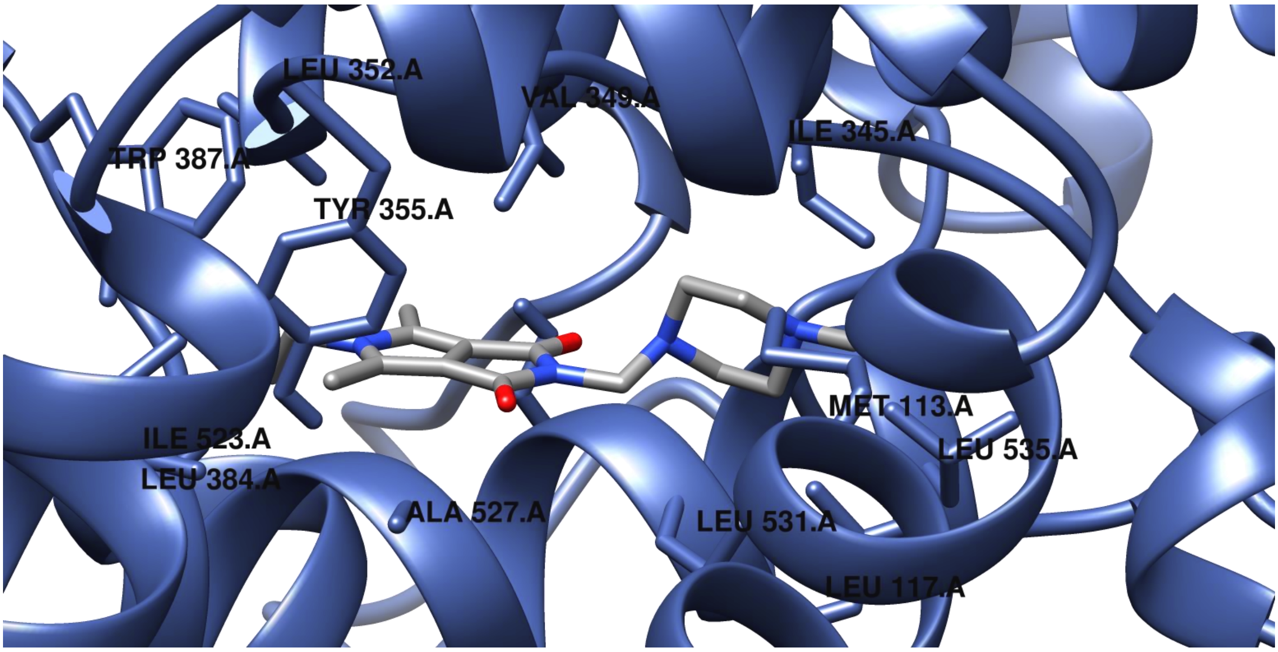 LEU 352.A<br>VAL 349.A<br>ILE 345.A<br>TRP 387.A<br>TYR 355.A<br>MET 113.A<br>LEU 535.A<br>ILE 523.A<br>LEU 384.A<br>ALA 527.A<br>LEU 531.A<br>LEU 117.A
**COX-2**
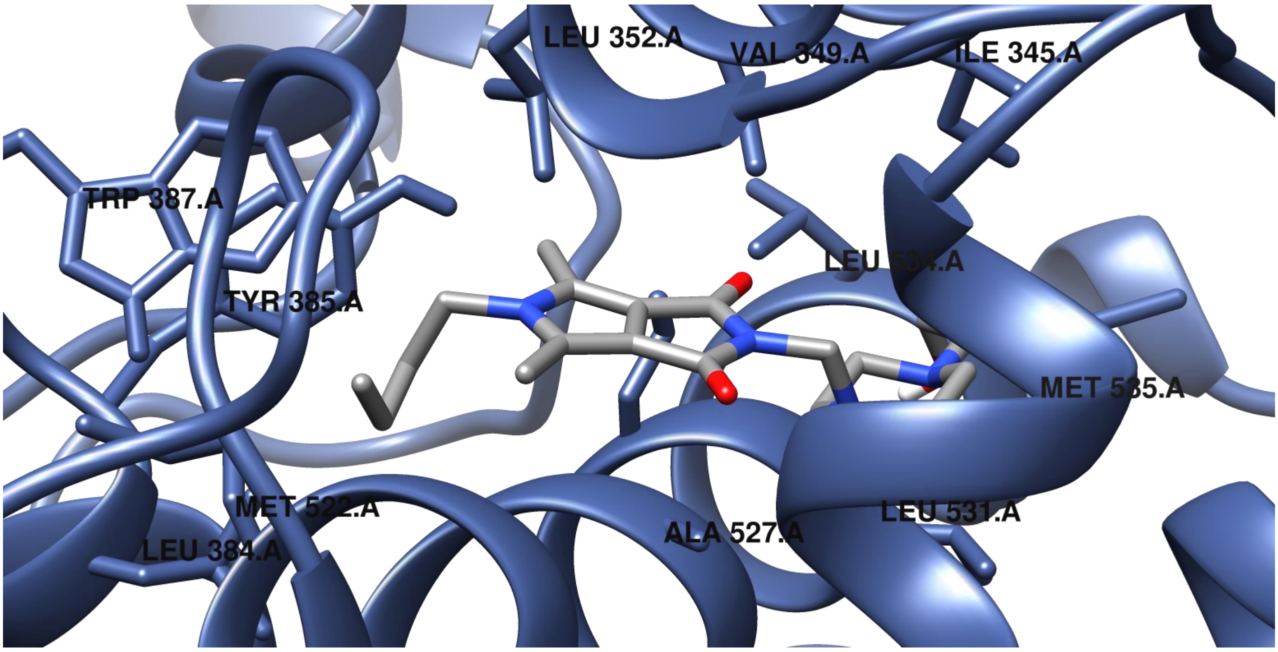 LEU 352.A<br>VAL 349.A<br>ILE 345.A<br>TRP 387.A<br>TYR 385.A<br>MET 535.A<br>MET 522.A<br>LEU 384.A<br>ALA 527.A<br>LEU 531.A

2k

COX-1	COX-2
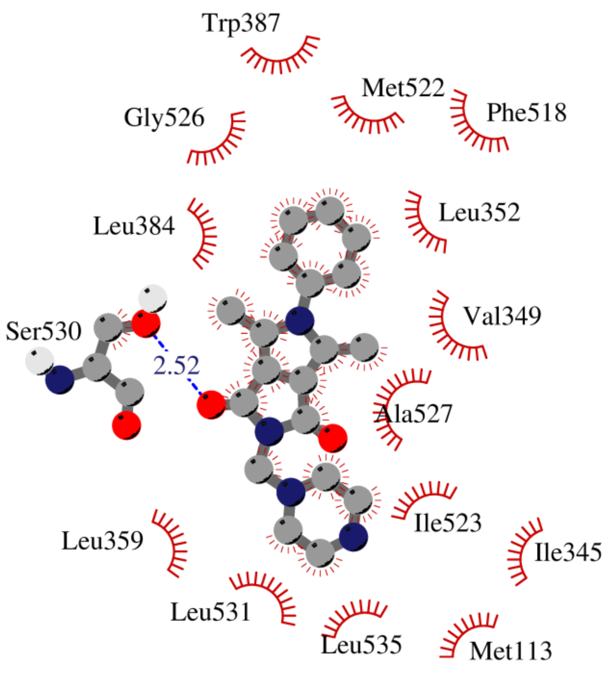	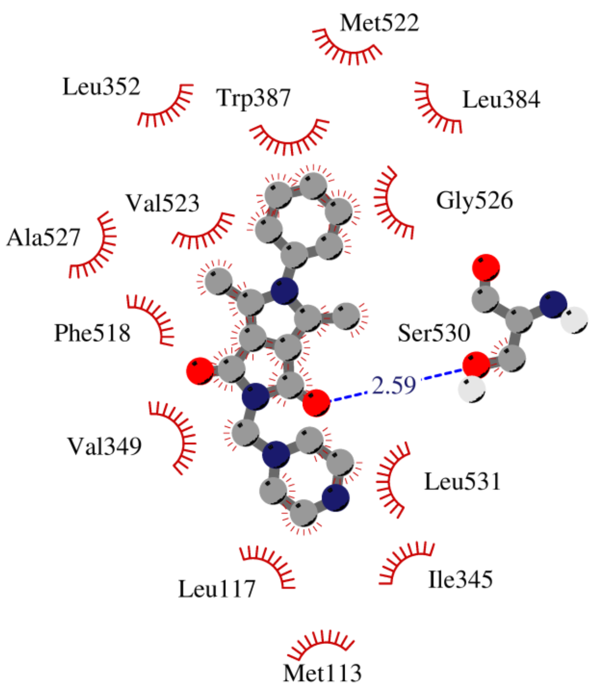

	COX-1	COX-2
Free enthalpy of binding [kcal/mol]	-8.22	-8.79
Inhibition constant [nM]	948	362

COX-1

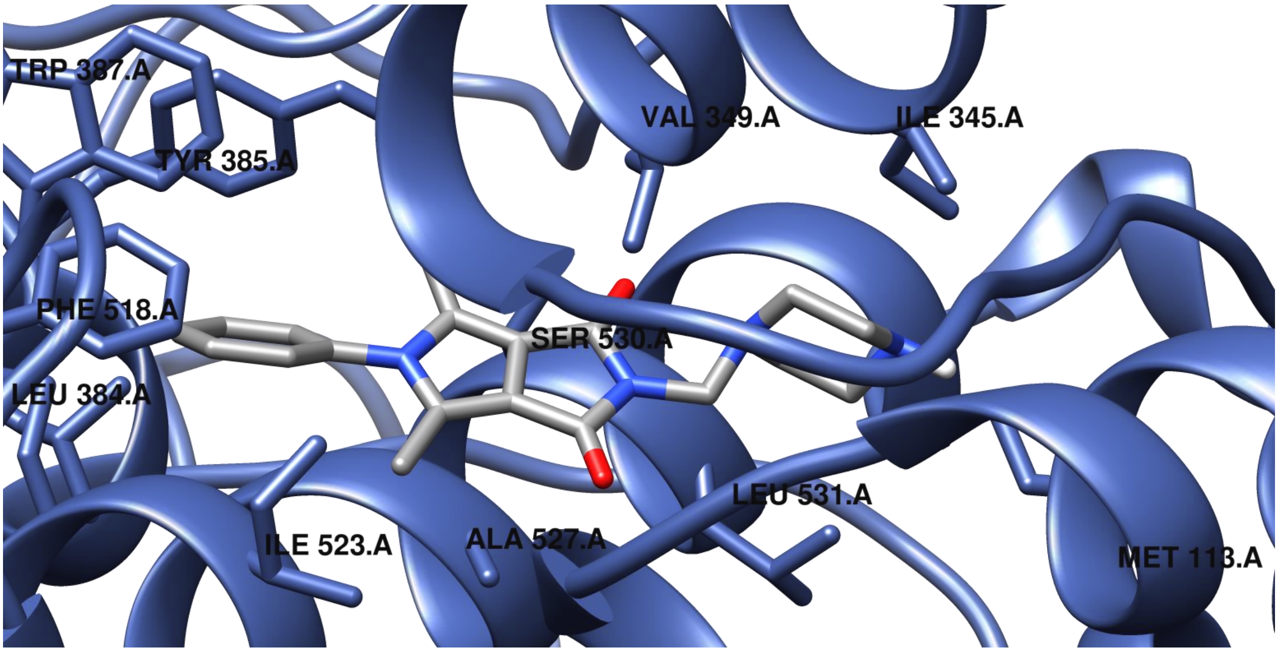
TRP 387.A
VAL 349.A
ILE 345.A
TYR 385.A
PHE 518.A
SER 530.A
LEU 384.A
LEU 531.A
ILE 523.A
ALA 527.A
MET 113.A

COX-2

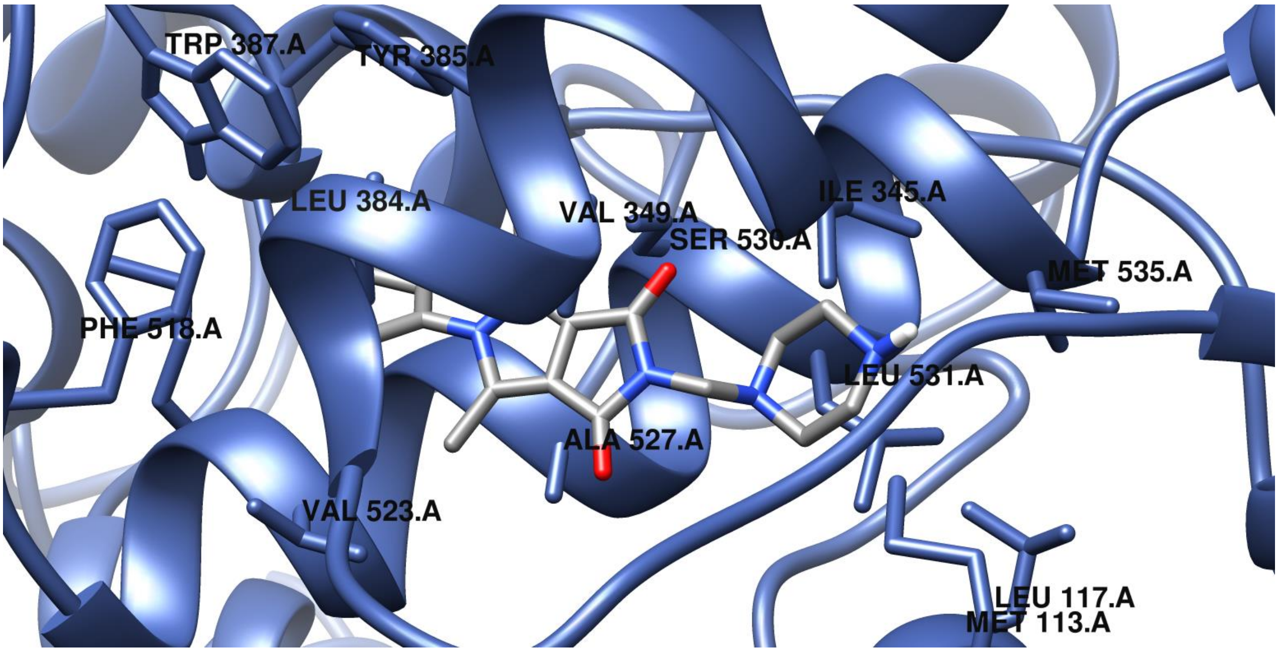
TRP 387.A
TYR 385.A
LEU 384.A
VAL 349.A
ILE 345.A
SER 530.A
MET 535.A
PHE 518.A
LEU 531.A
ALA 527.A
VAL 523.A
LEU 117.A
MET 113.A

2l

COX-1	COX-2

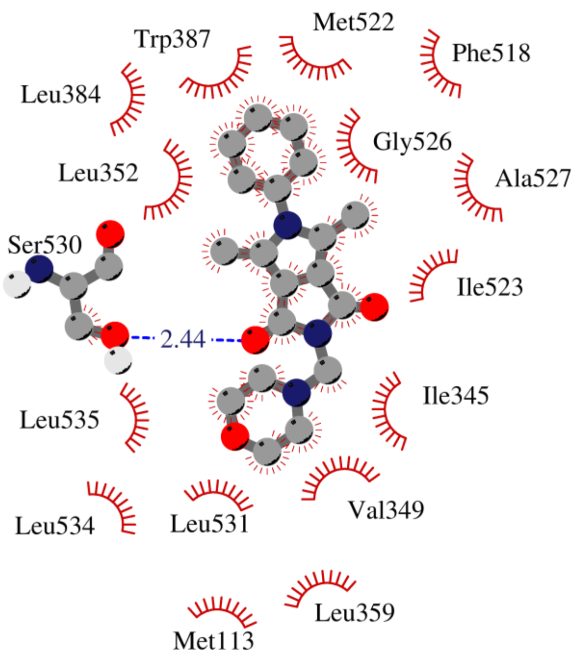

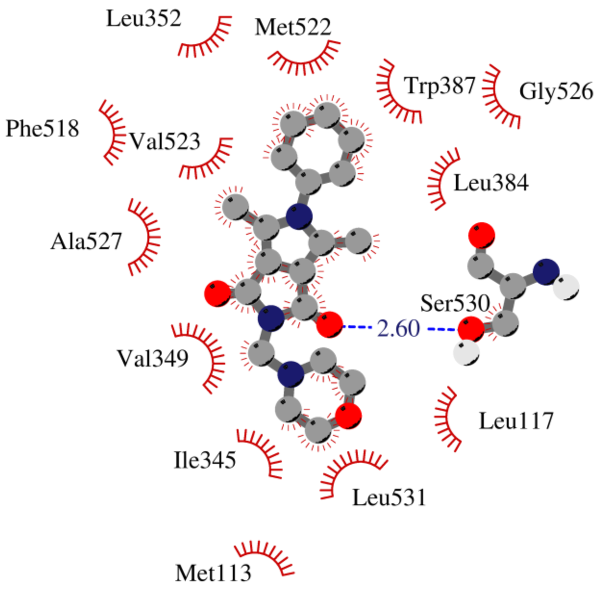

	COX-1	COX-2
Free enthalpy of binding [kcal/mol]	-8.28	-8.64
Inhibition constant [nM]	853	460

COX-1
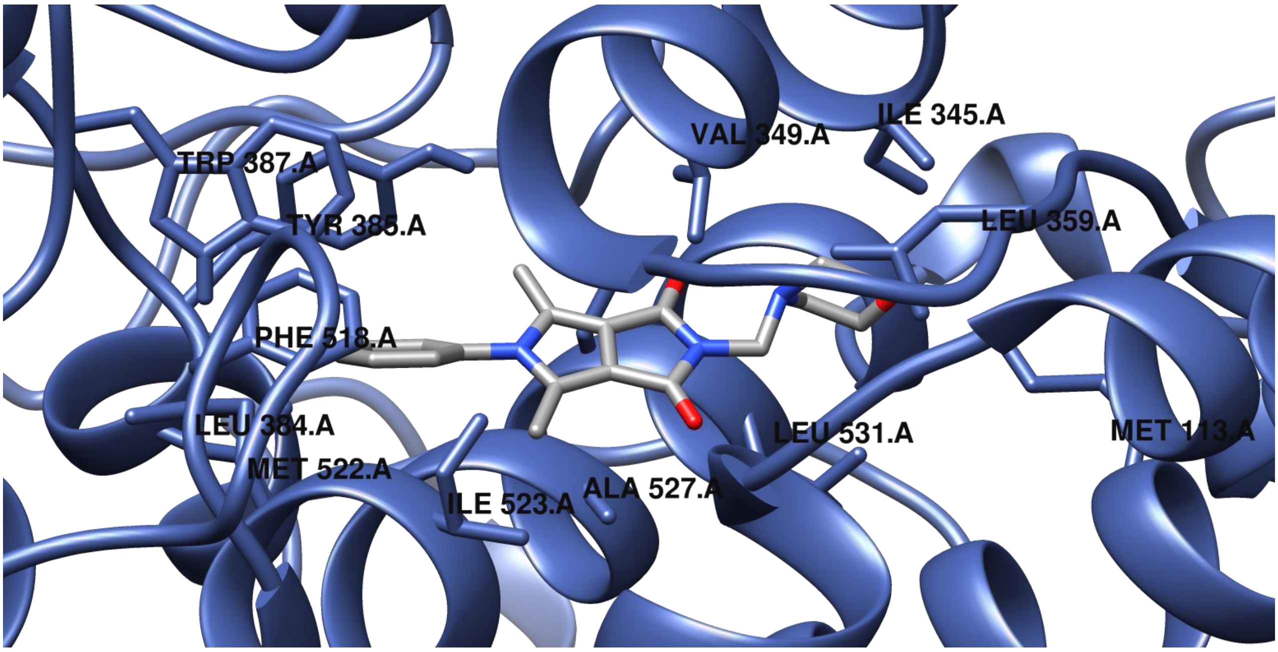
**COX-2**
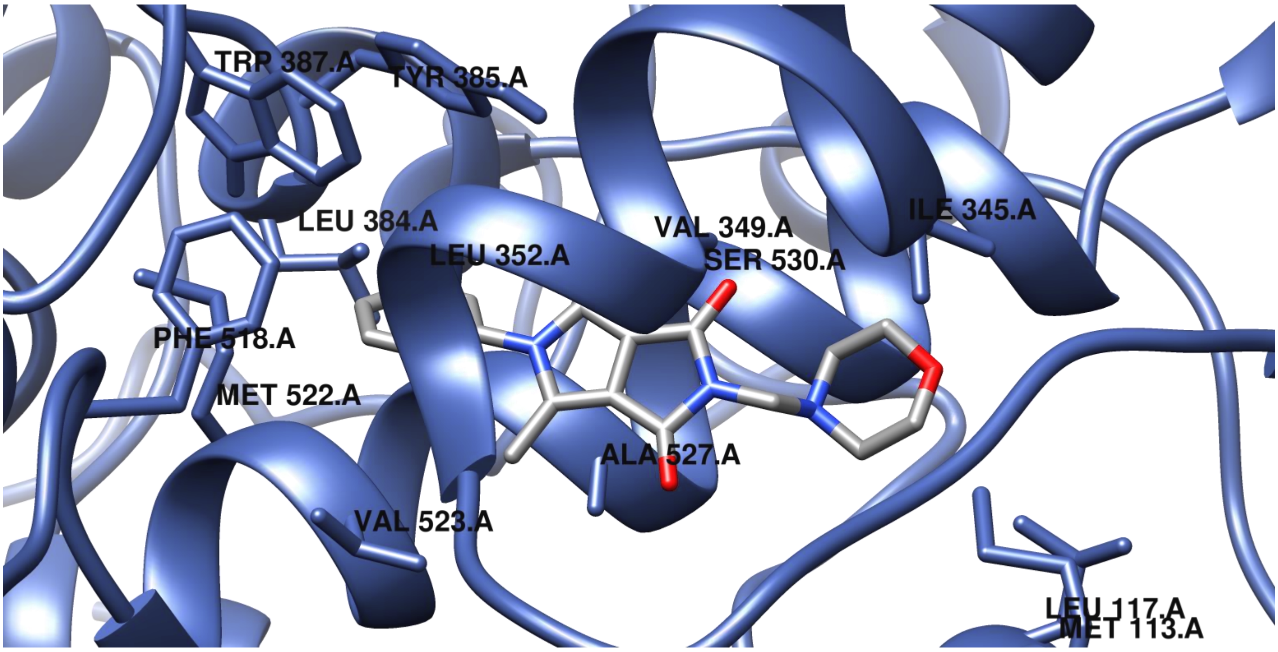

TRP 387.A
TYR 385.A
PHE 518.A
LEU 384.A
MET 522.A
ILE 523.A
ALA 527.A
VAL 349.A
ILE 345.A
LEU 359.A
LEU 531.A
MET 113.A

TRP 387.A
TYR 385.A
LEU 384.A
LEU 352.A
VAL 349.A
SER 530.A
ILE 345.A
PHE 518.A
MET 522.A
ALA 527.A
VAL 523.A
LEU 117.A
MET 113.A

2m

COX-1	COX-2

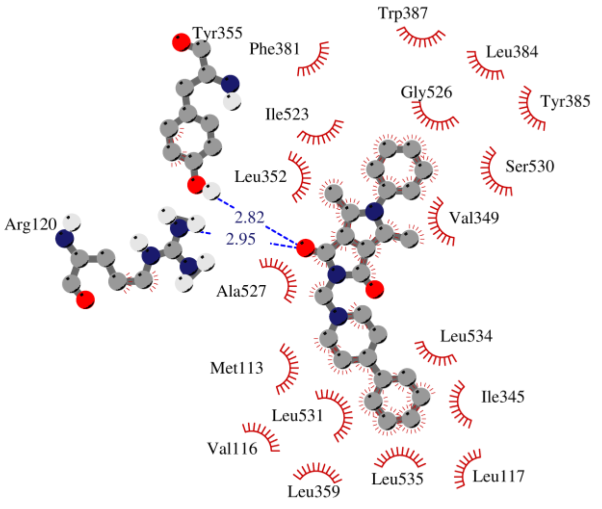

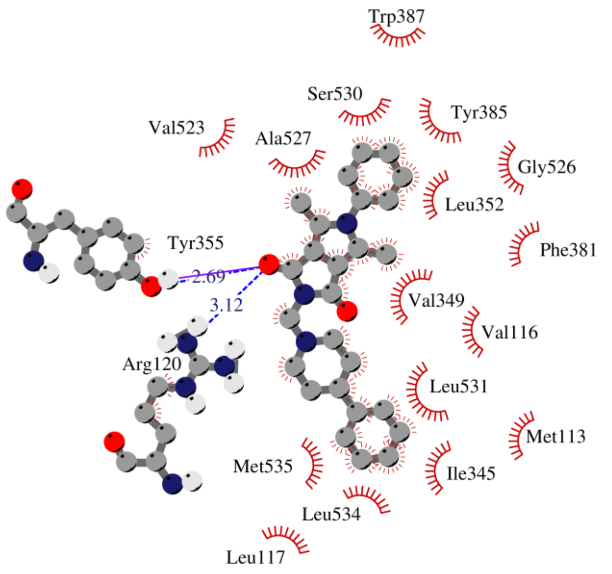

	COX-1	COX-2
Free enthalpy of binding [kcal/mol]	-10.04	-11.03
Inhibition constant [nM]	43.81	8.16

COX-1

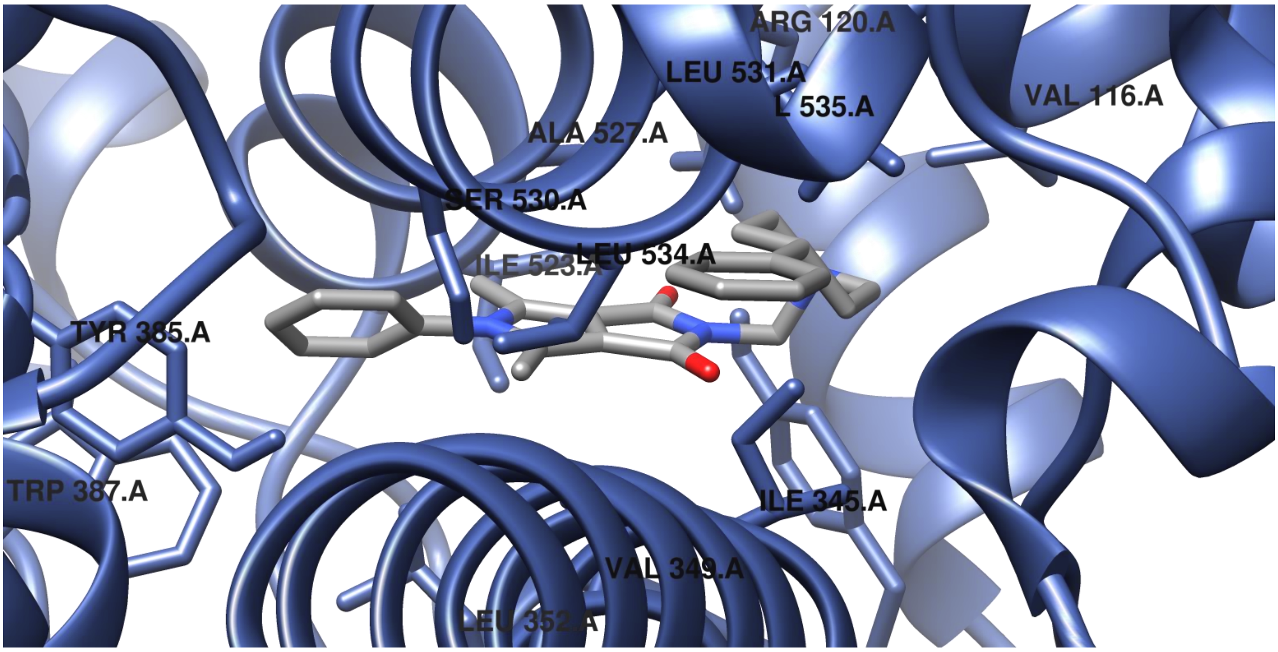
ARG 120.A
LEU 531.A
L 535.A
VAL 116.A
ALA 527.A
SER 530.A
ILE 523.A
LEU 534.A
TYR 385.A
TRP 387.A
ILE 345.A
VAL 349.A
LEU 352.A

COX-2

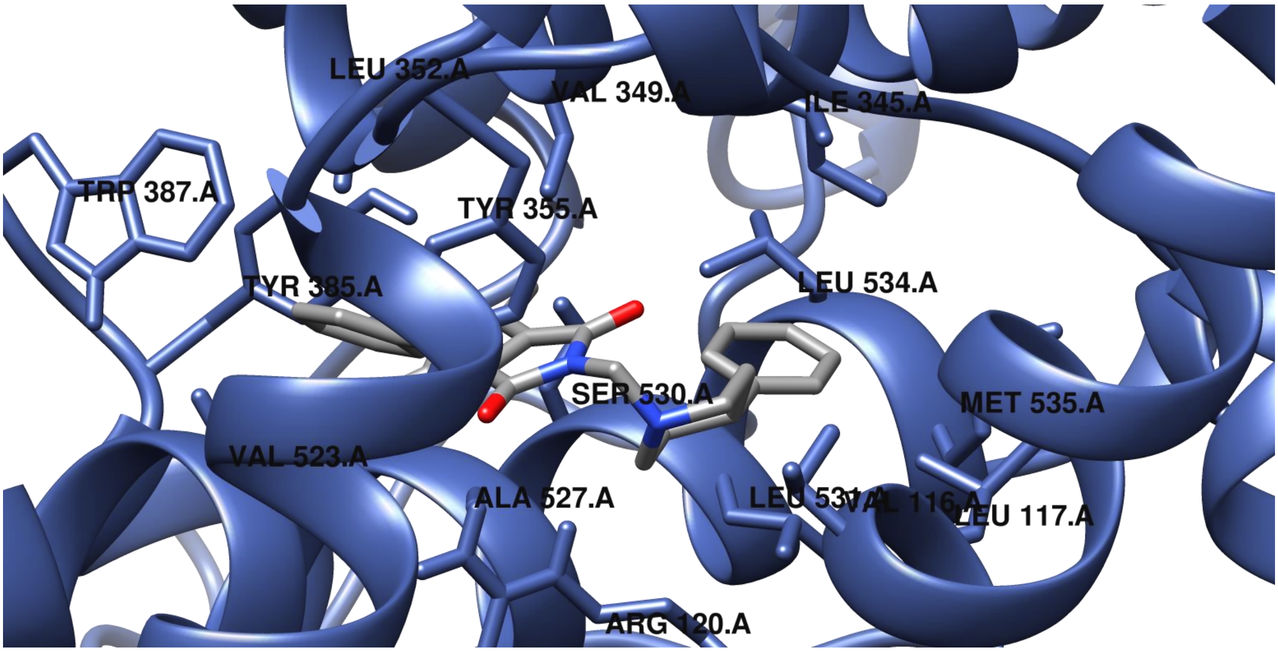
LEU 352.A
VAL 349.A
ILE 345.A
TRP 387.A
TYR 355.A
TYR 385.A
LEU 534.A
SER 530.A
MET 535.A
VAL 523.A
ALA 527.A
LEU 531.A
VAL 116.A
LEU 117.A
ARG 120.A

**2n**	
**COX-1**	**COX-2**

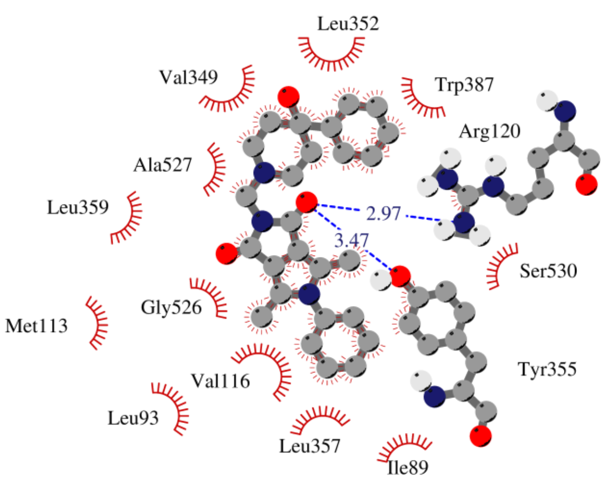

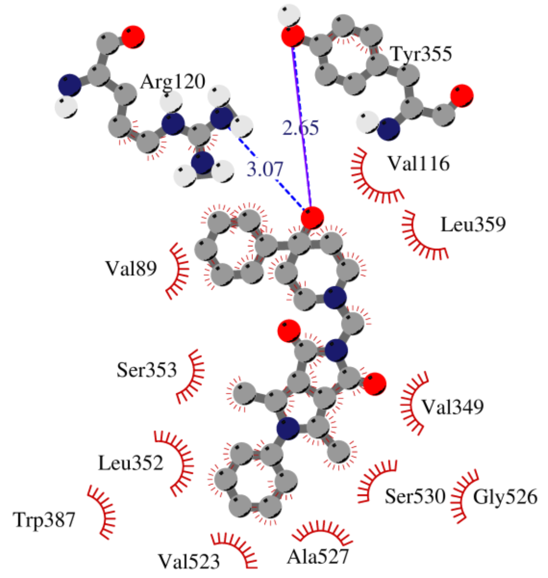

	COX-1	COX-2
Free enthalpy of binding [kcal/mol]	-7.38	-10.33
Inhibition constant [nM]	3900	26.92

COX-1

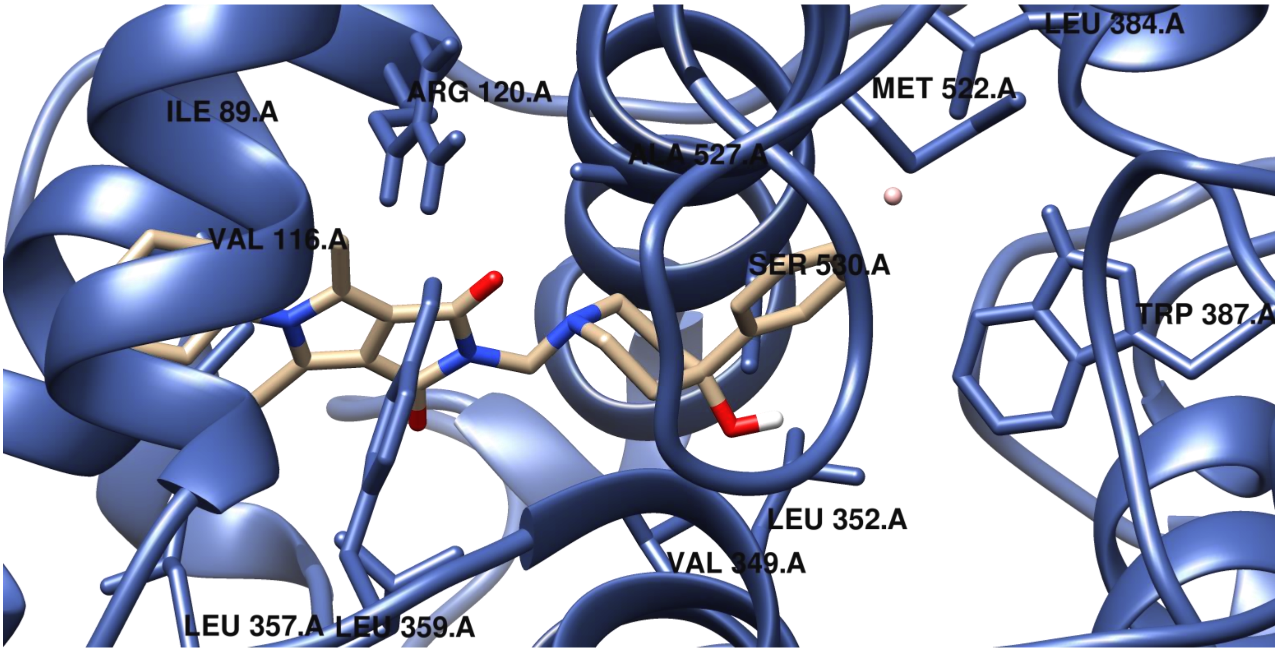
LEU 384.A
ARG 120.A
MET 522.A
ILE 89.A
ALA 527.A
VAL 116.A
SER 530.A
TRP 387.A
LEU 352.A
VAL 349.A
LEU 357.A
LEU 359.A

COX-2

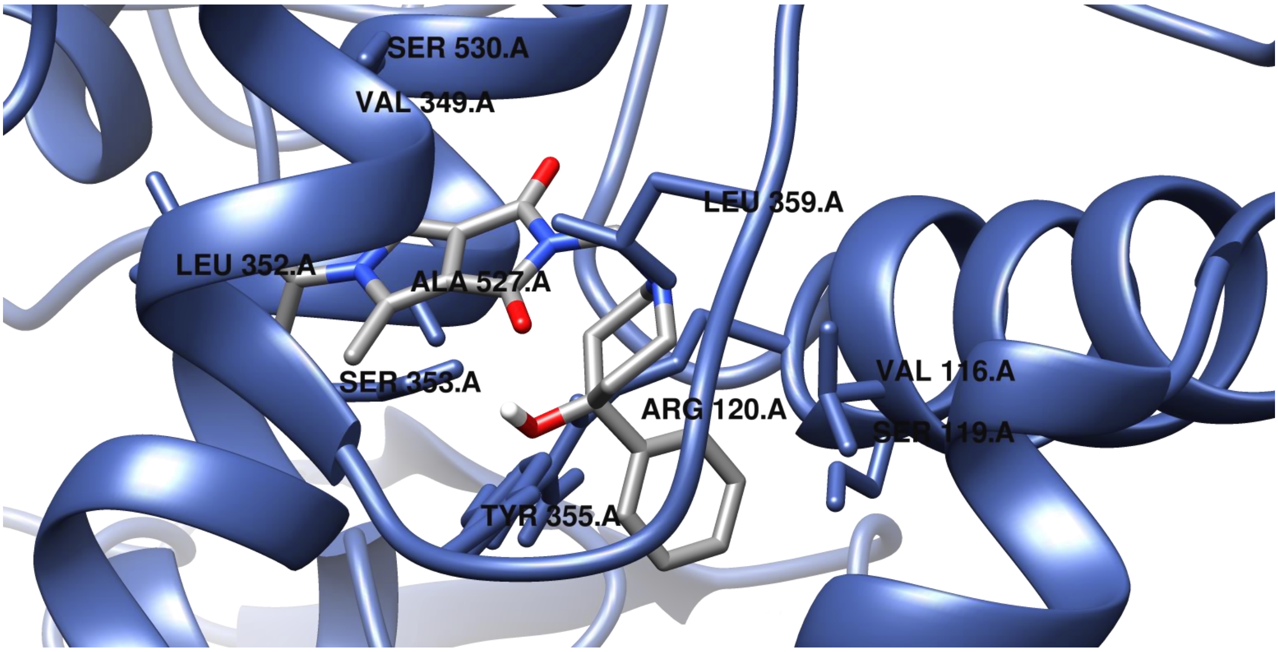
SER 530.A
VAL 349.A
LEU 359.A
LEU 352.A
ALA 527.A
SER 353.A
VAL 116.A
ARG 120.A
SER 119.A
TYR 355.A

2o

COX-1	COX-2
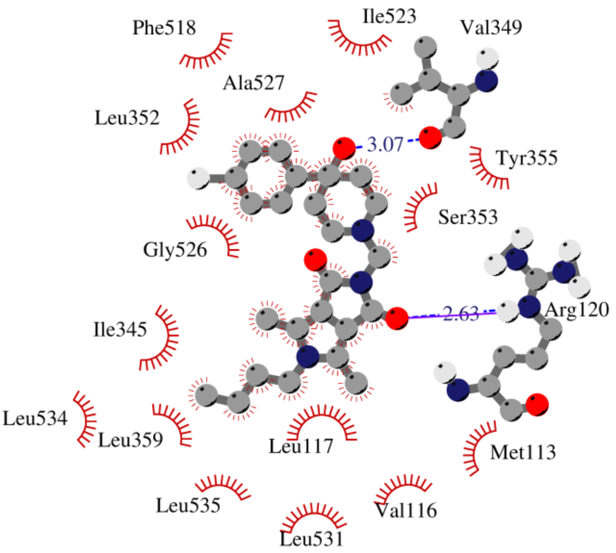	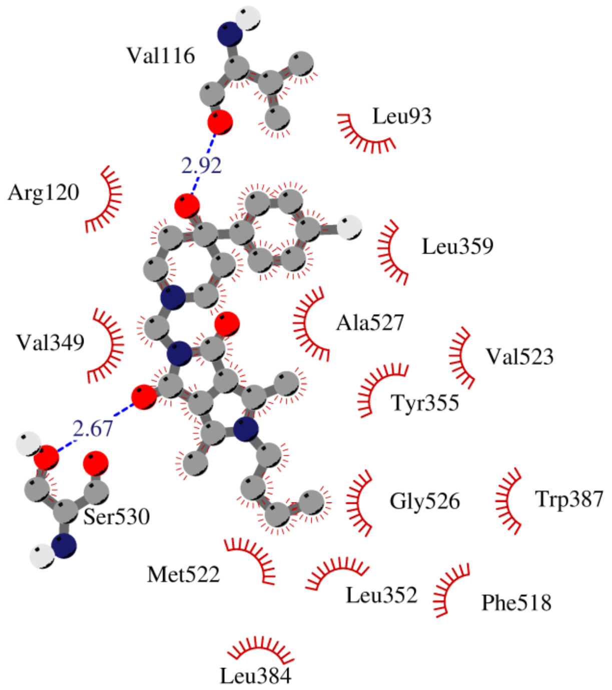

	COX-1	COX-2
Free enthalpy of binding [kcal/mol]	-8.94	-9.81
Inhibition constant [nM]	279	64

COX-1

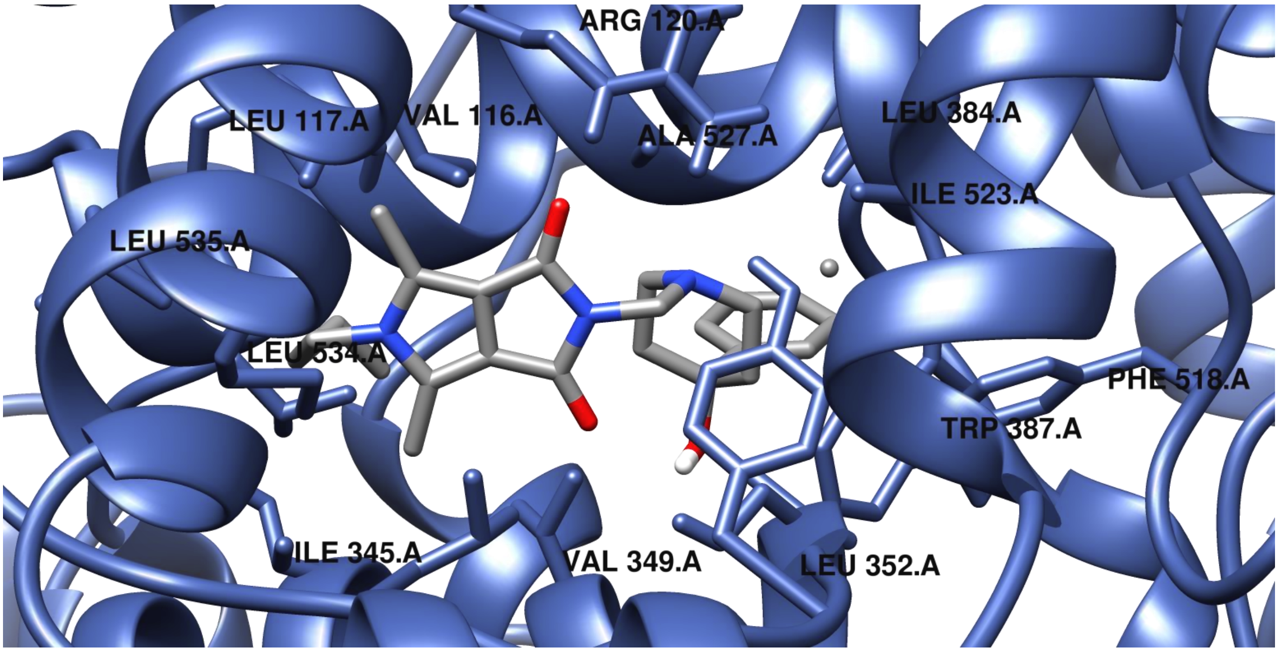
ARG 120.A
LEU 117.A
VAL 116.A
ALA 527.A
LEU 384.A
ILE 523.A
LEU 535.A
LEU 534.A
PHE 518.A
TRP 387.A
ILE 345.A
VAL 349.A
LEU 352.A

COX-2

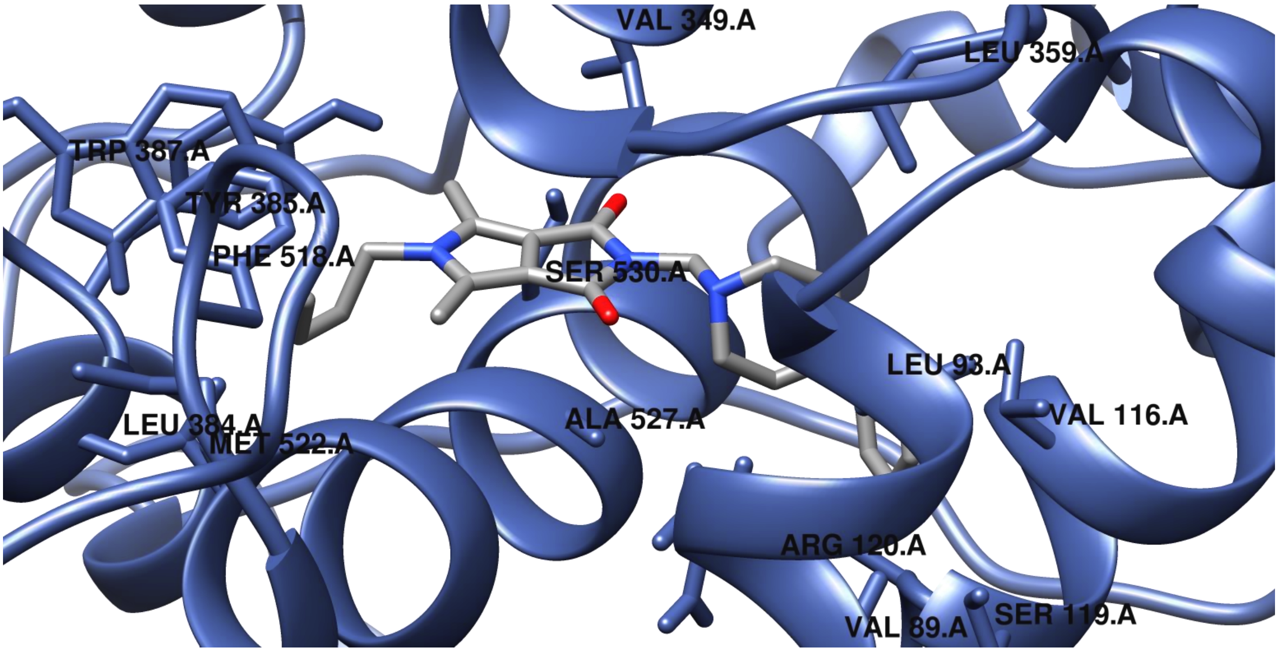
VAL 349.A
LEU 359.A
TRP 387.A
TYR 385.A
PHE 518.A
SER 530.A
LEU 93.A
VAL 116.A
LEU 384.A
MET 522.A
ALA 527.A
ARG 120.A
VAL 89.A
SER 119.A

2p

COX-1	COX-2
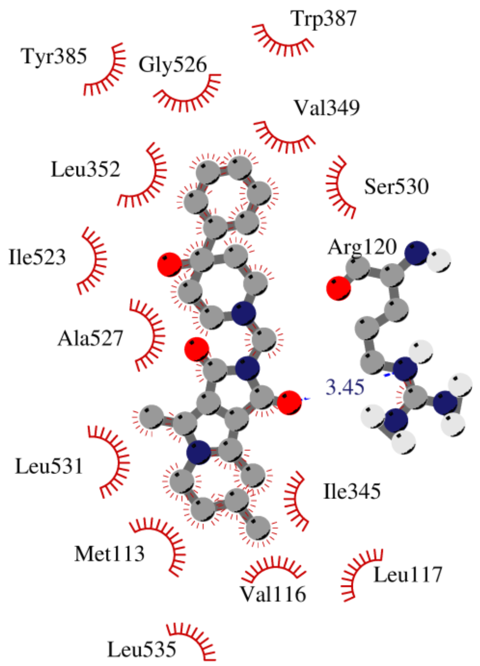	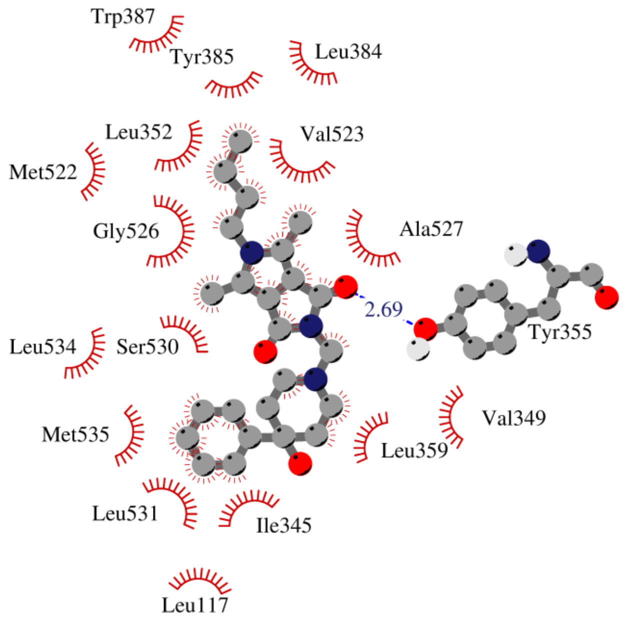

	COX-1	COX-2
Free enthalpy of binding [kcal/mol]	-8.9	-10.02
Inhibition constant [nM]	300	45

COX-1

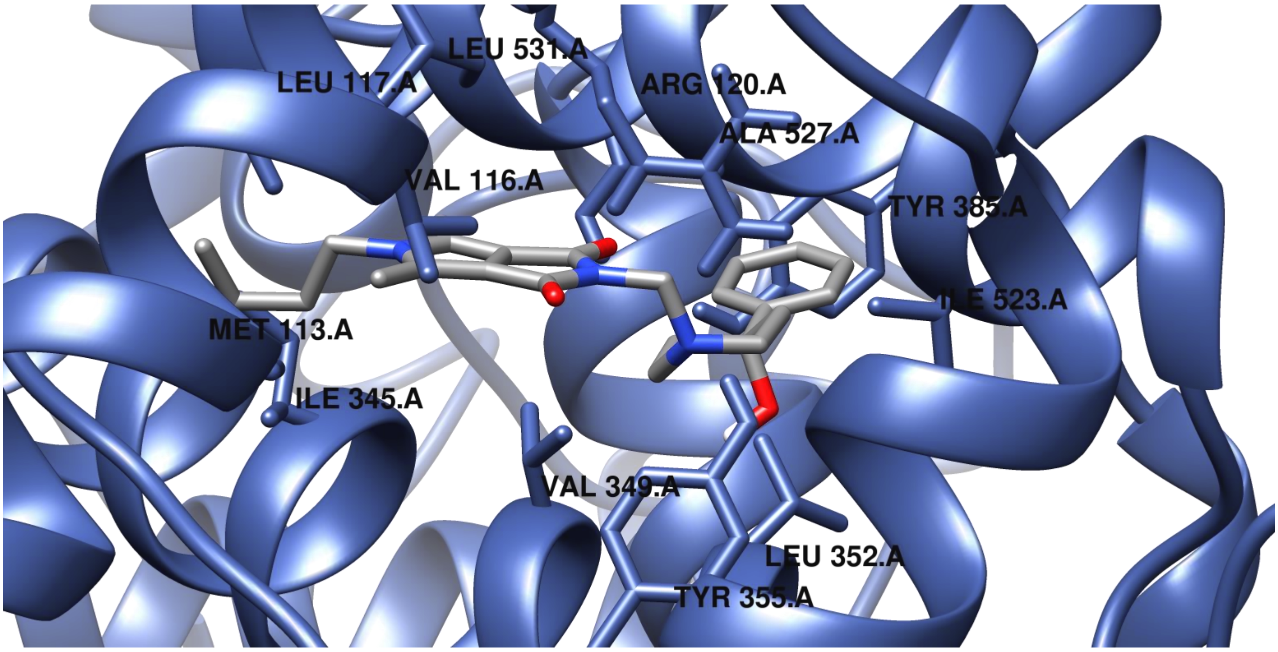

LEU 531.A
LEU 117.A
ARG 120.A
ALA 527.A
VAL 116.A
TYR 385.A
ILE 523.A
MET 113.A
ILE 345.A
VAL 349.A
LEU 352.A
TYR 355.A

COX-2

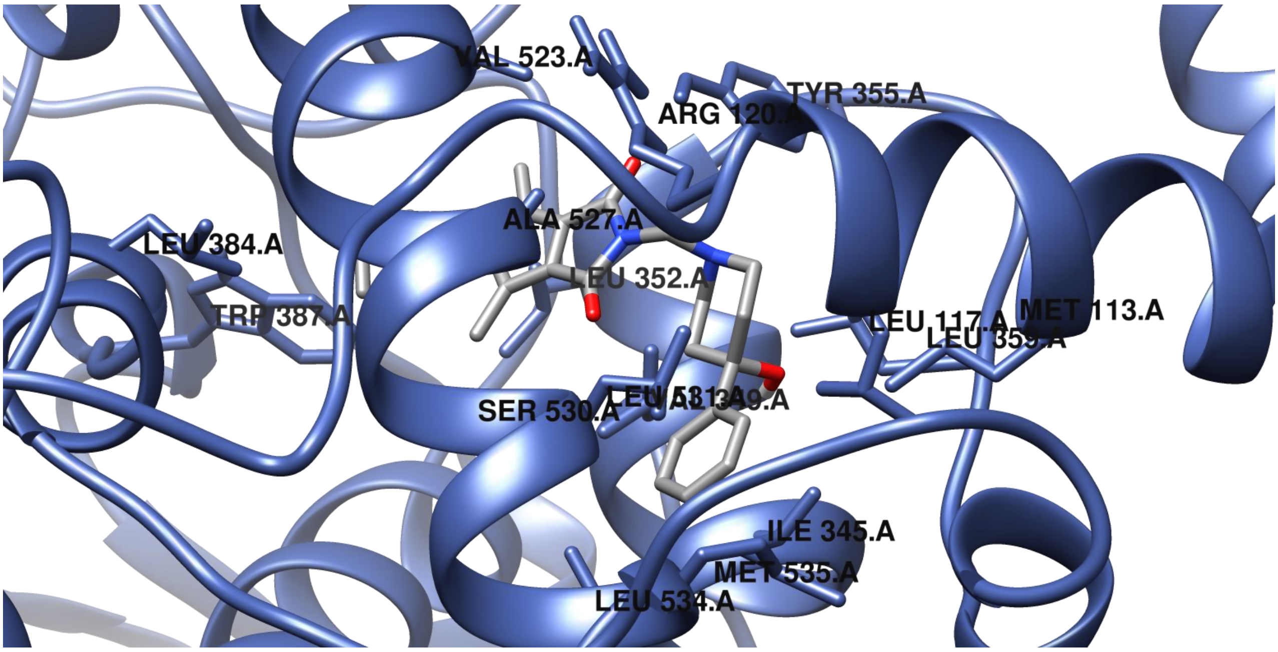

VAL 523.A
TYR 355.A
ARG 120.A
ALA 527.A
LEU 384.A
LEU 352.A
TRP 387.A
LEU 117.A
MET 113.A
SER 530.A
ILE 345.A
MET 535.A
LEU 534.A
