# Supplementary material for: Design and Synthesis of N-Substituted 3,4-Pyrroledicarboximides as Potential Anti-Inflammatory Agents
Source: Int J Mol Sci. 2021 Jan 30;22(3):1410. doi: 10.3390/ijms22031410 (PMC7866801; doi:10.3390/ijms22031410)

Window Display Report

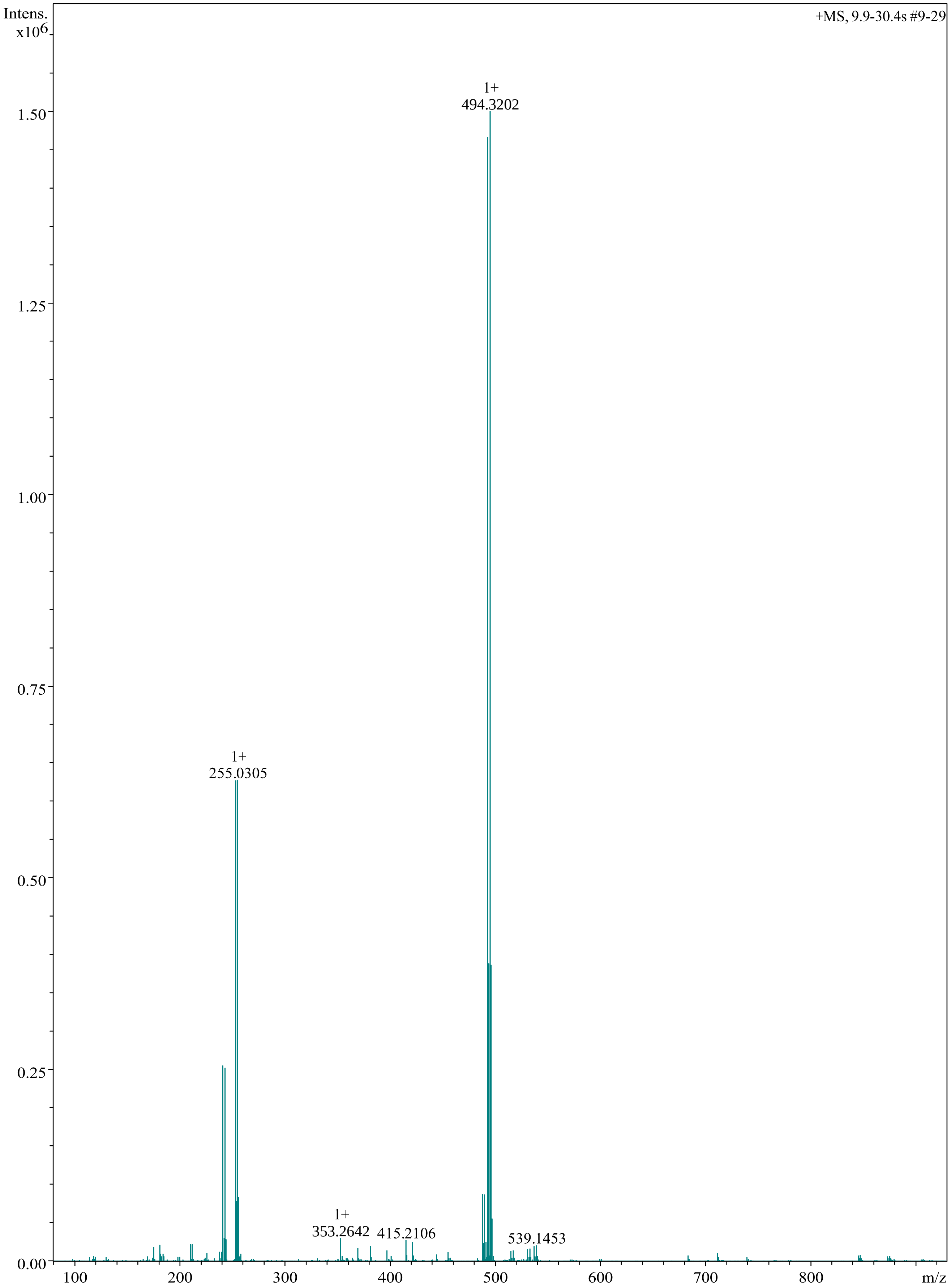

Window Display Report

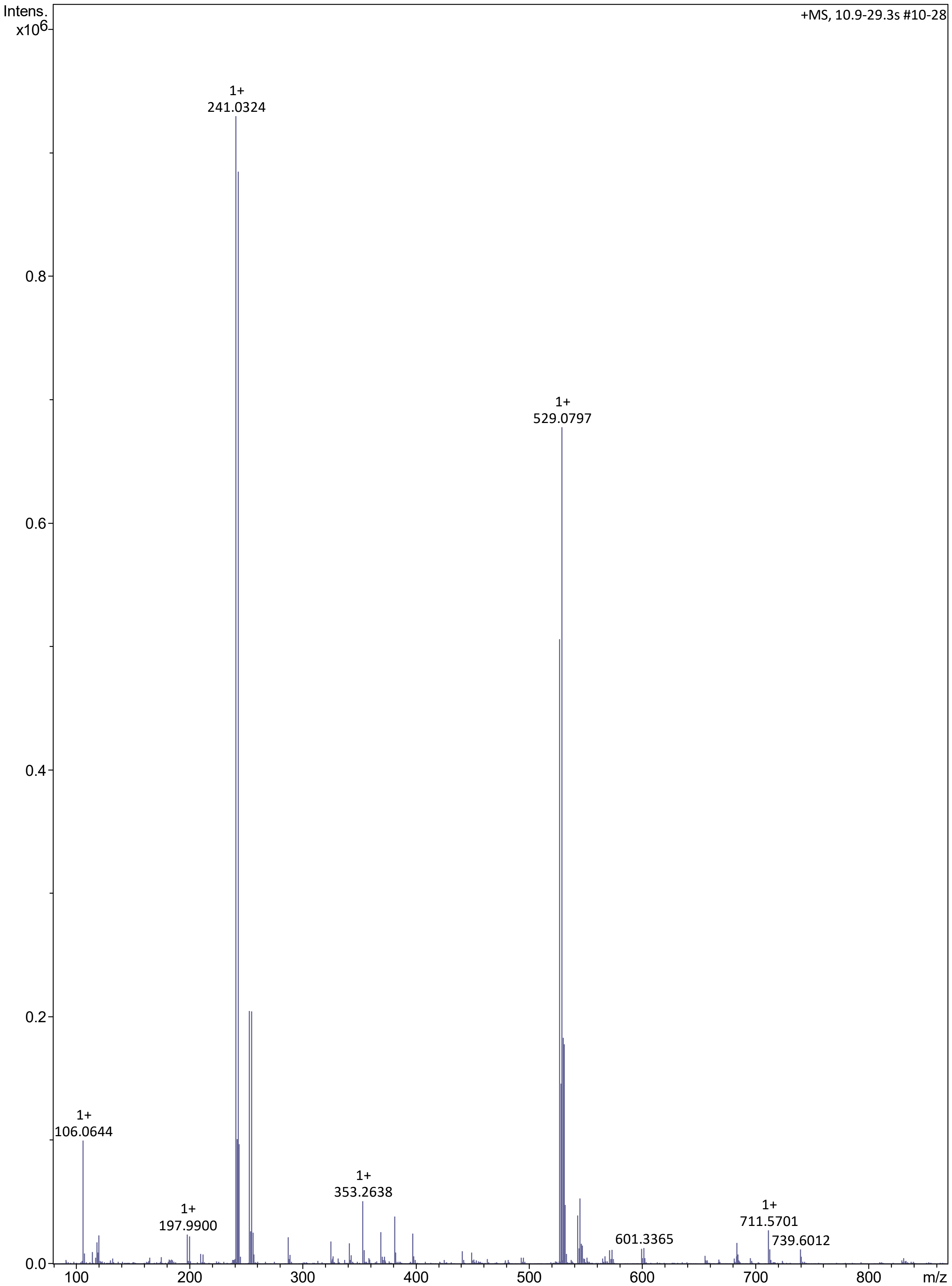

Window Display Report

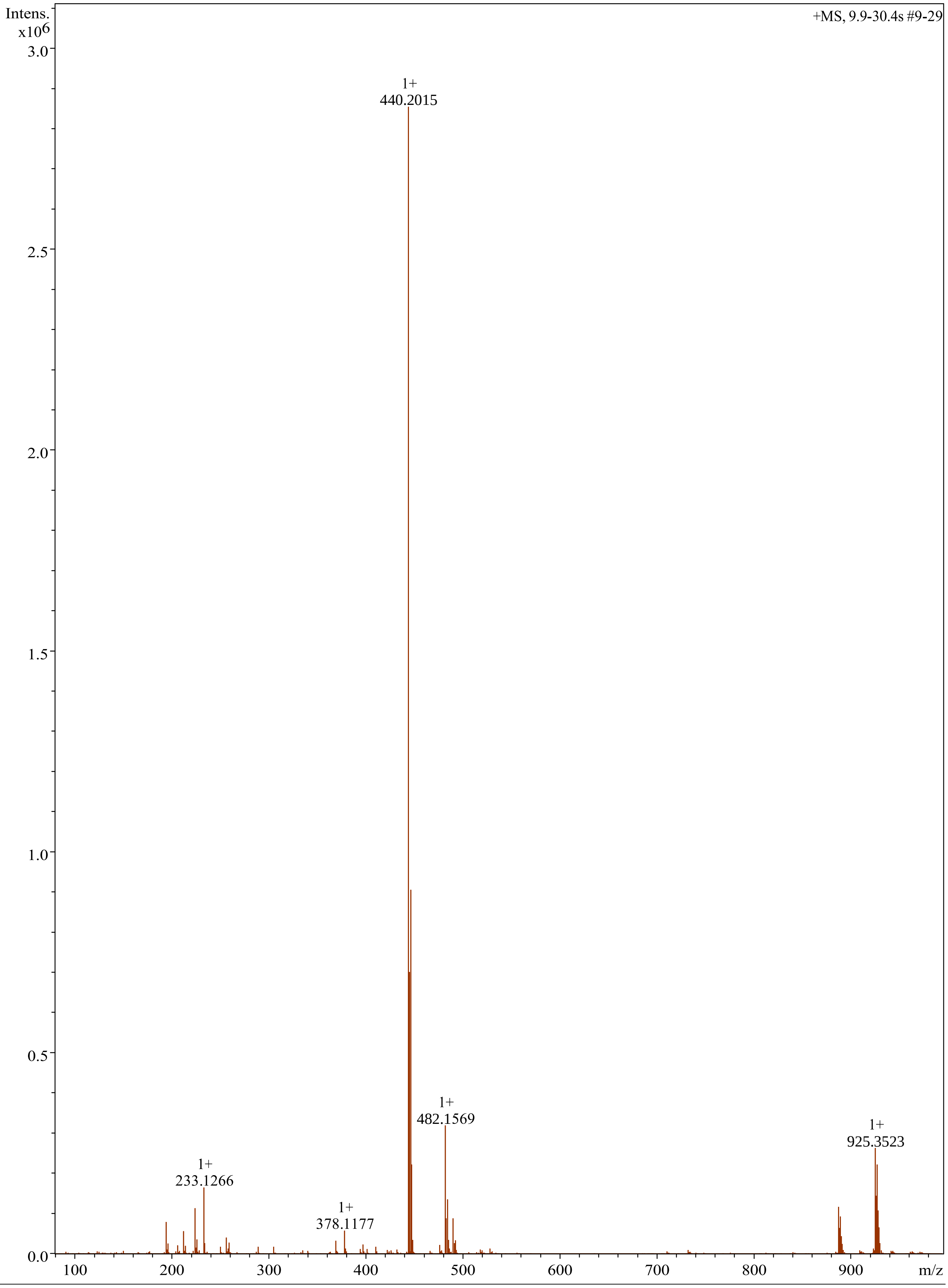

Window Display Report

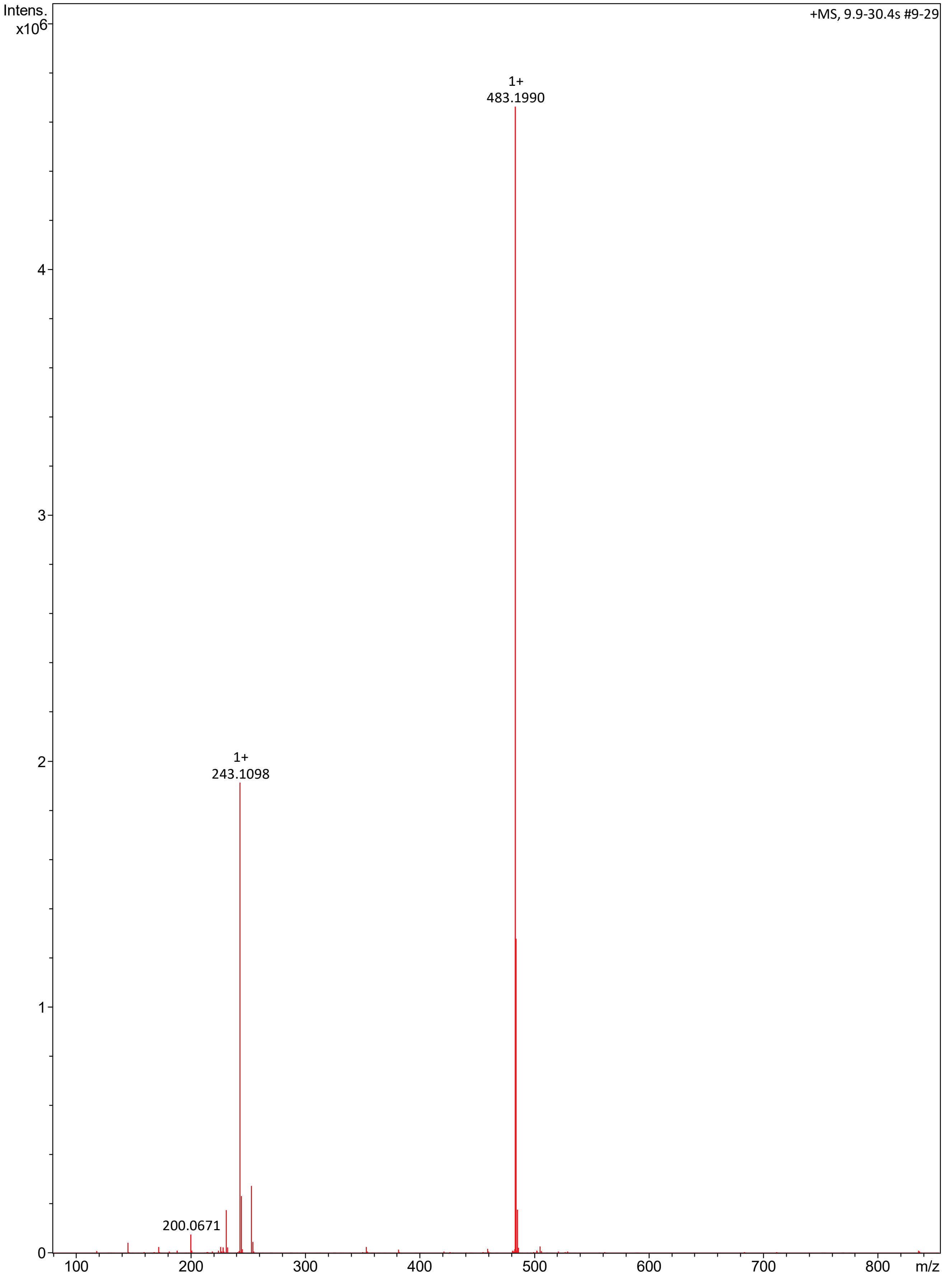

Window Display Report

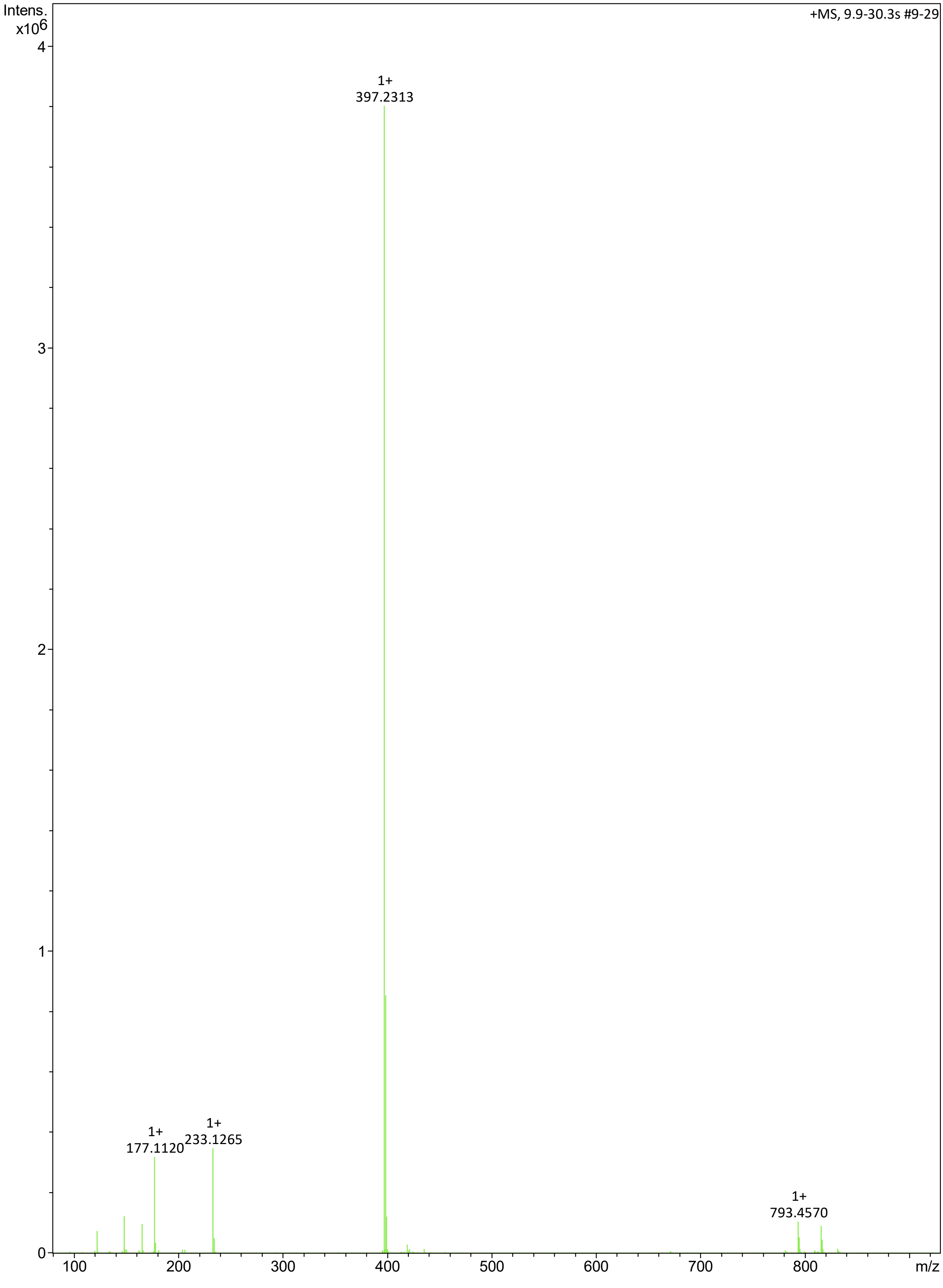

Window Display Report

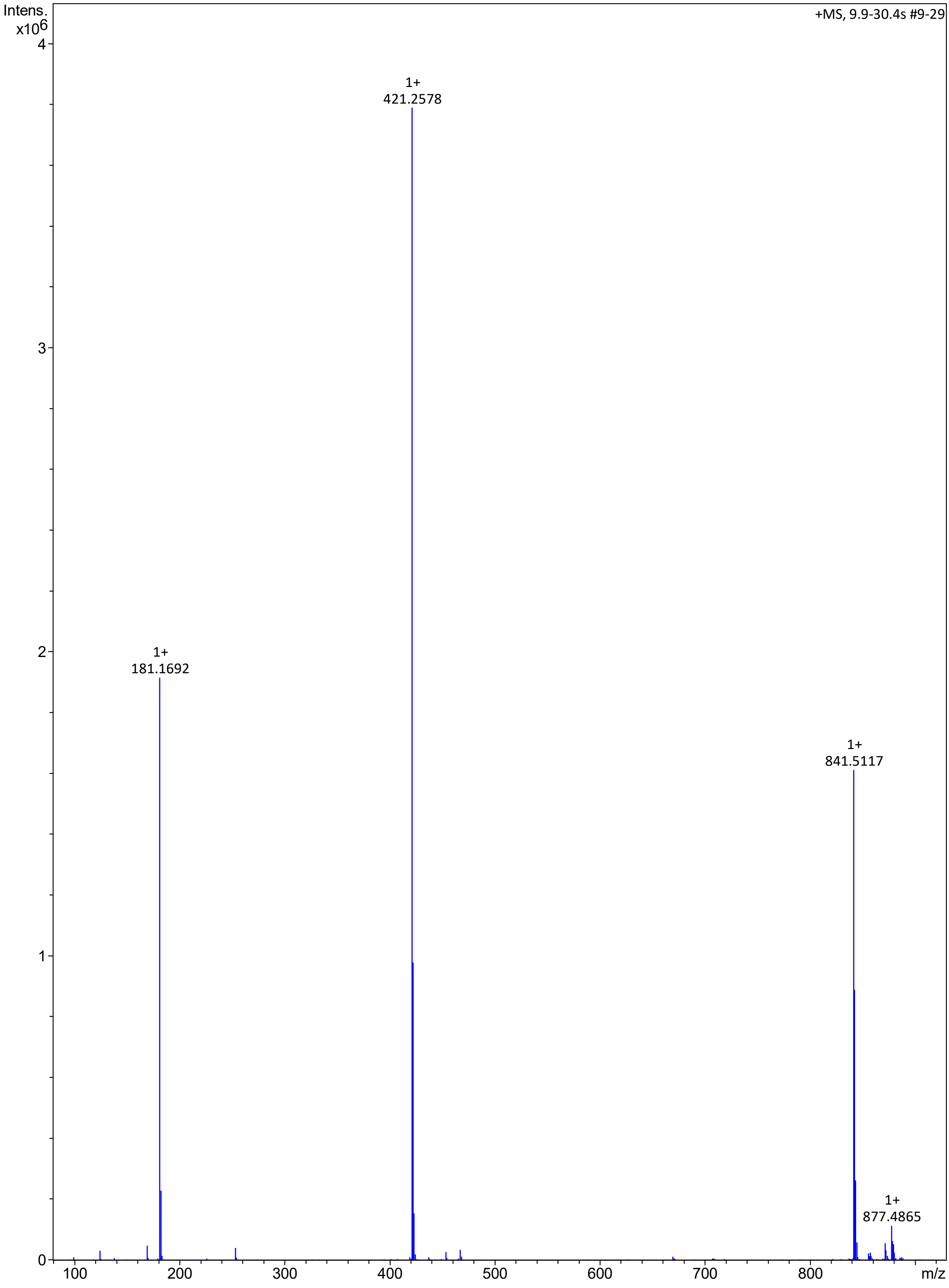

Window Display Report

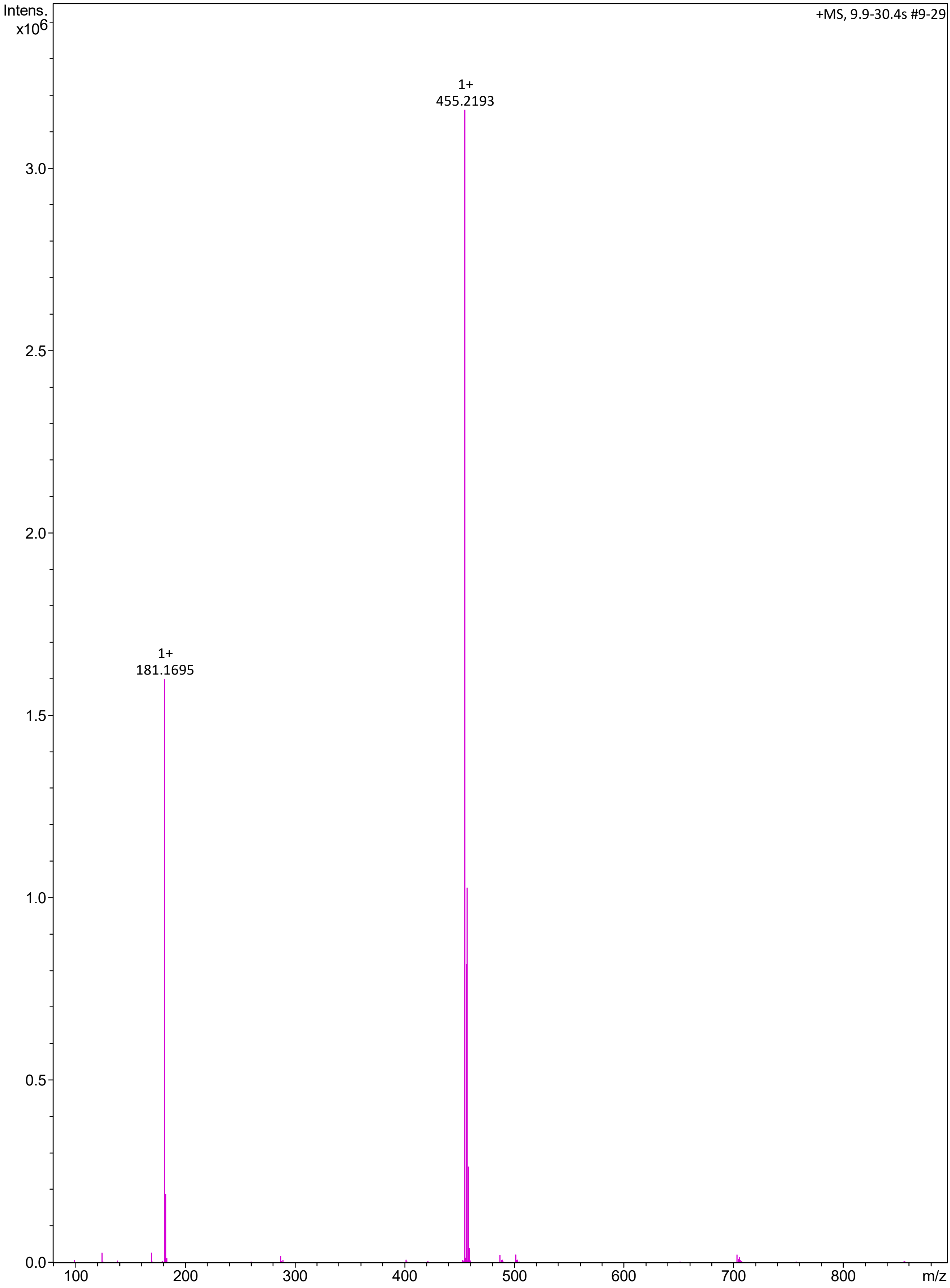

Window Display Report

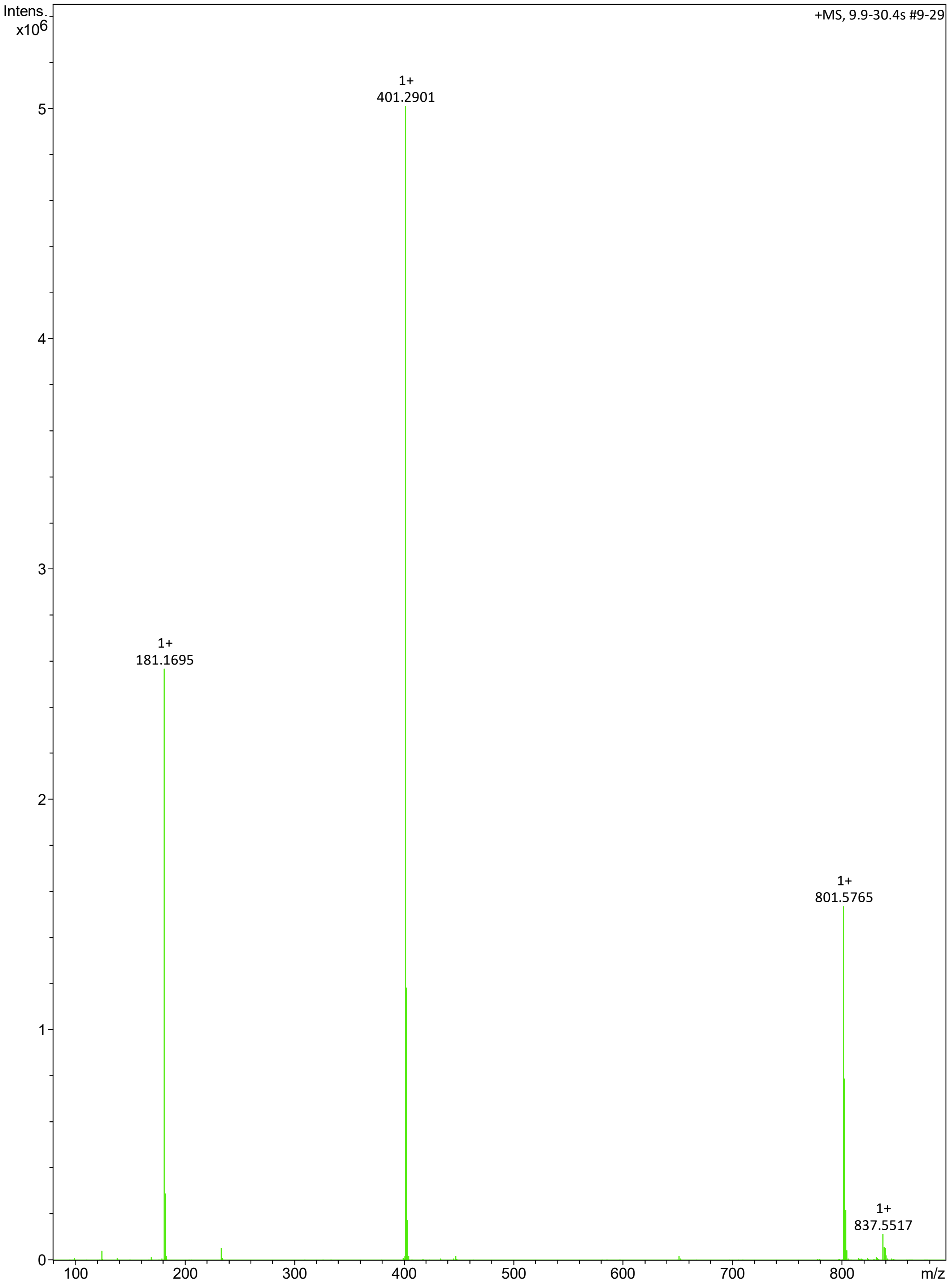

Window Display Report

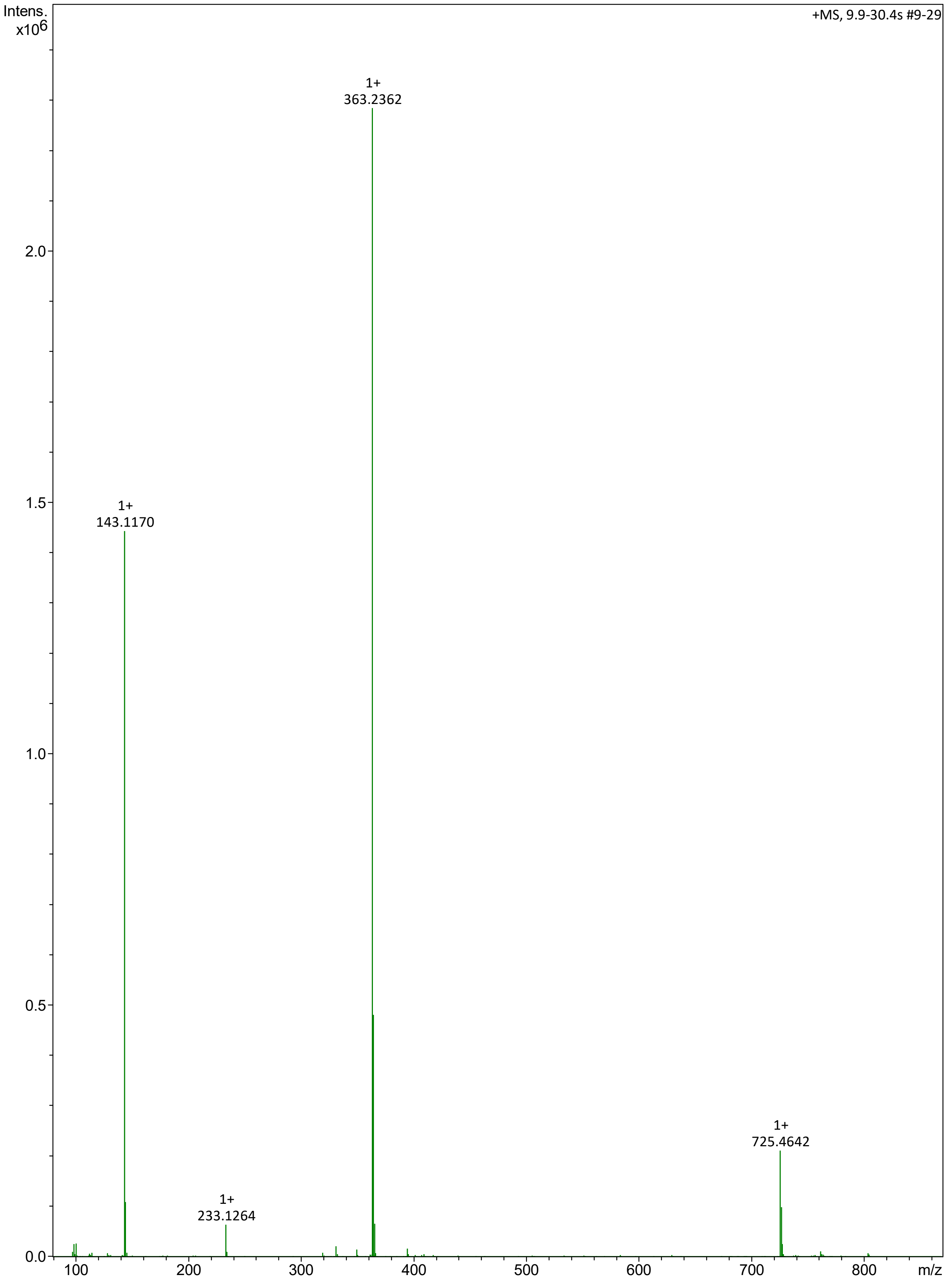

Window Display Report

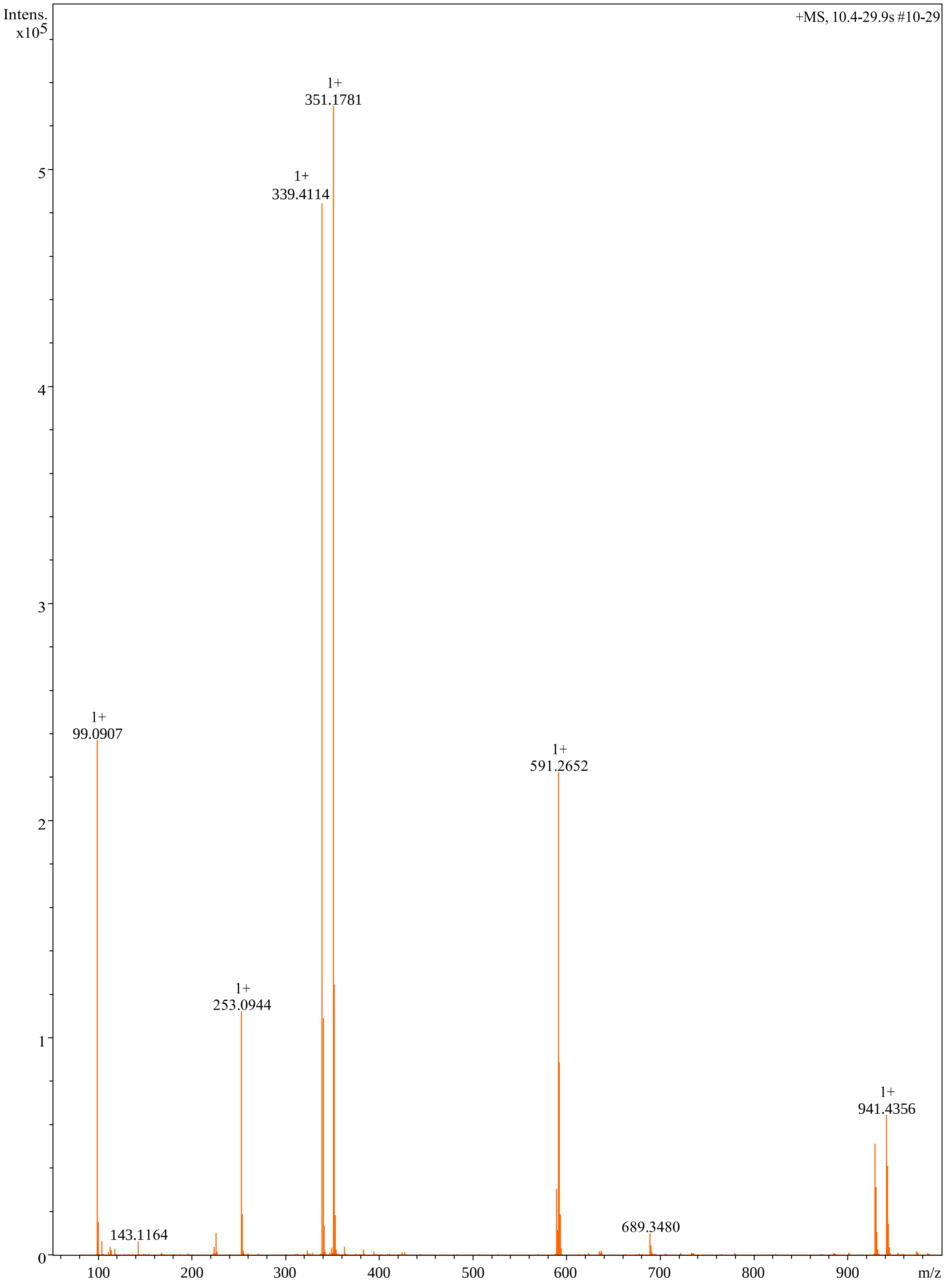

Window Display Report

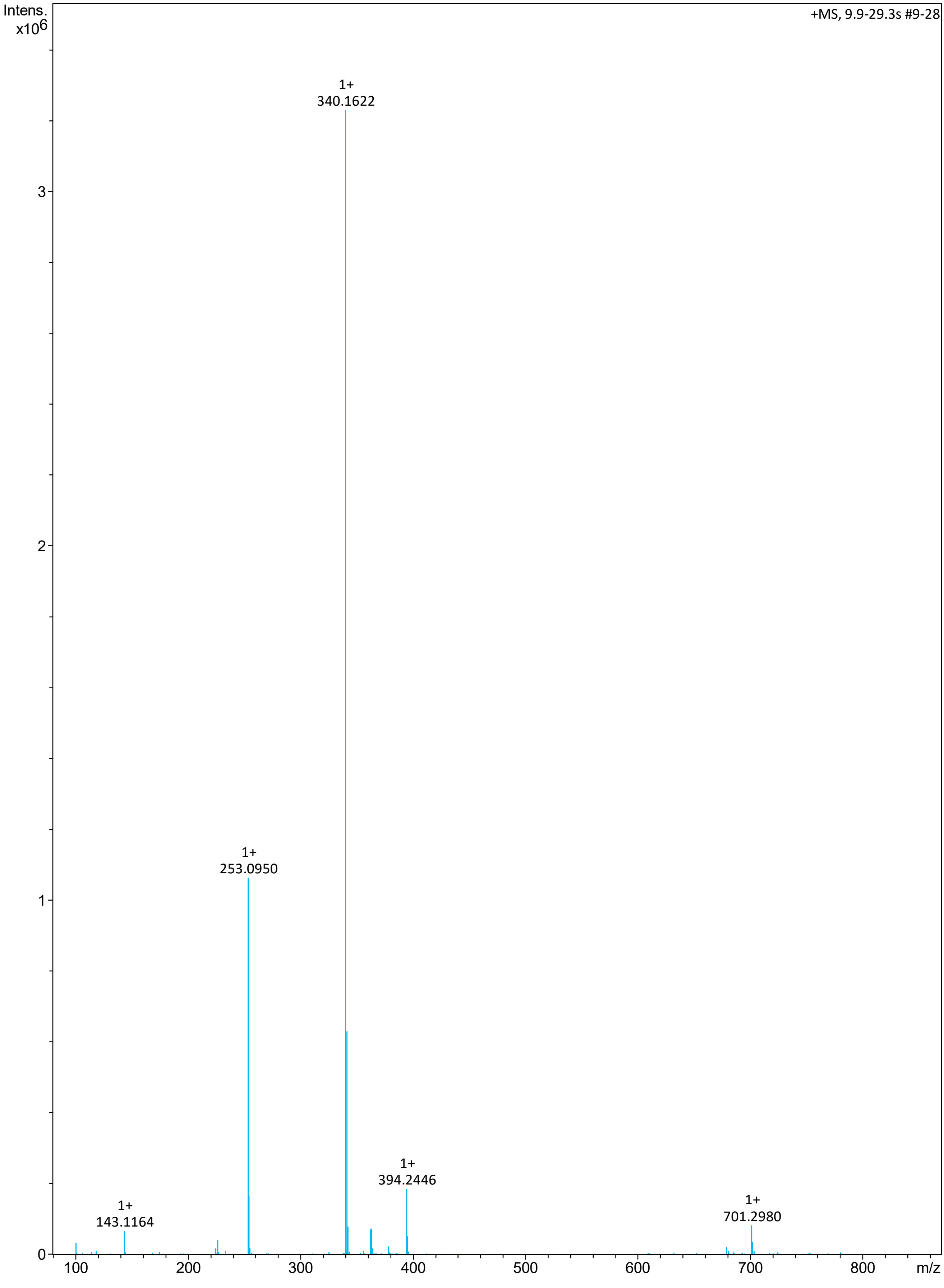

Window Display Report

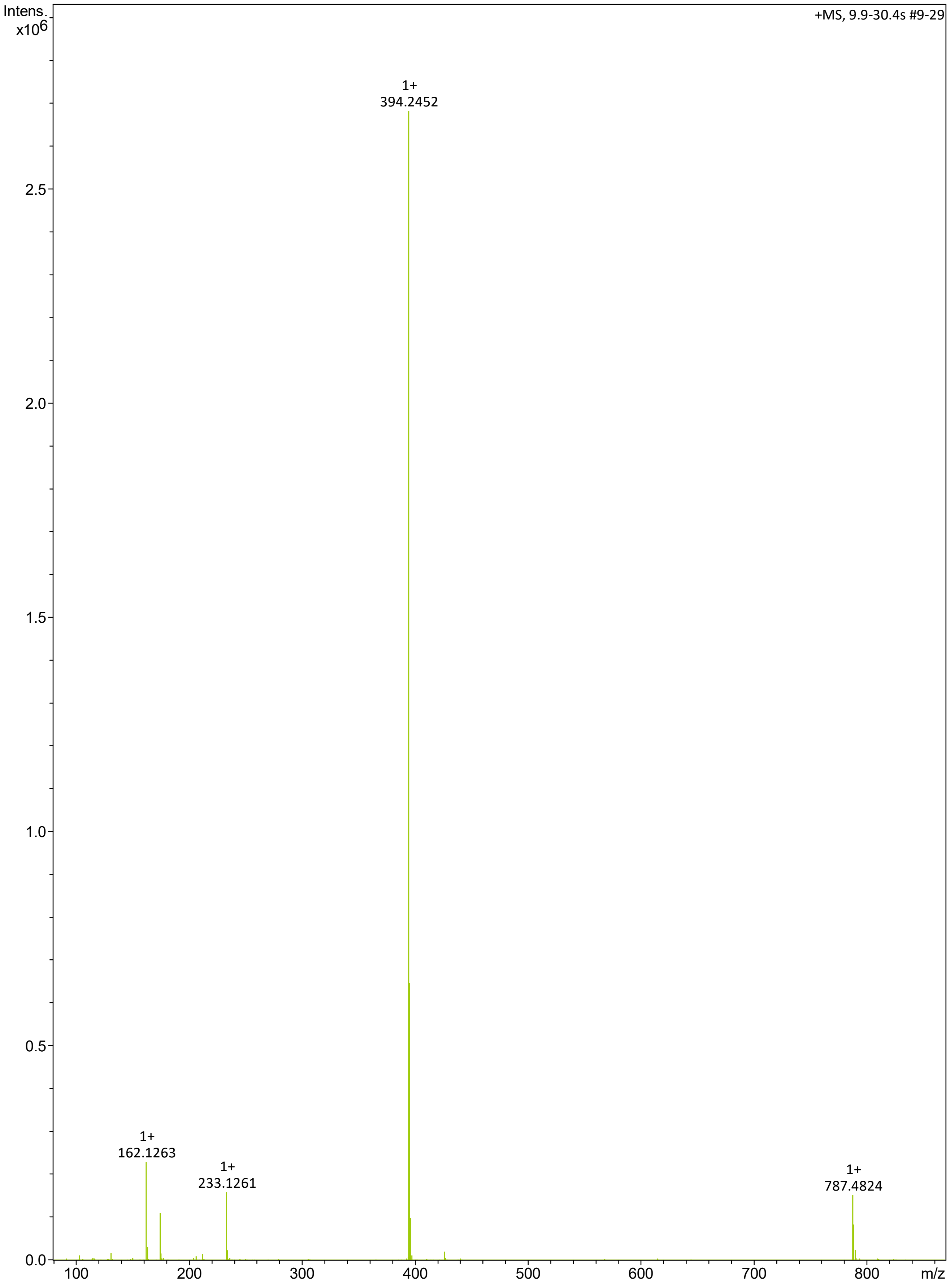

Window Display Report

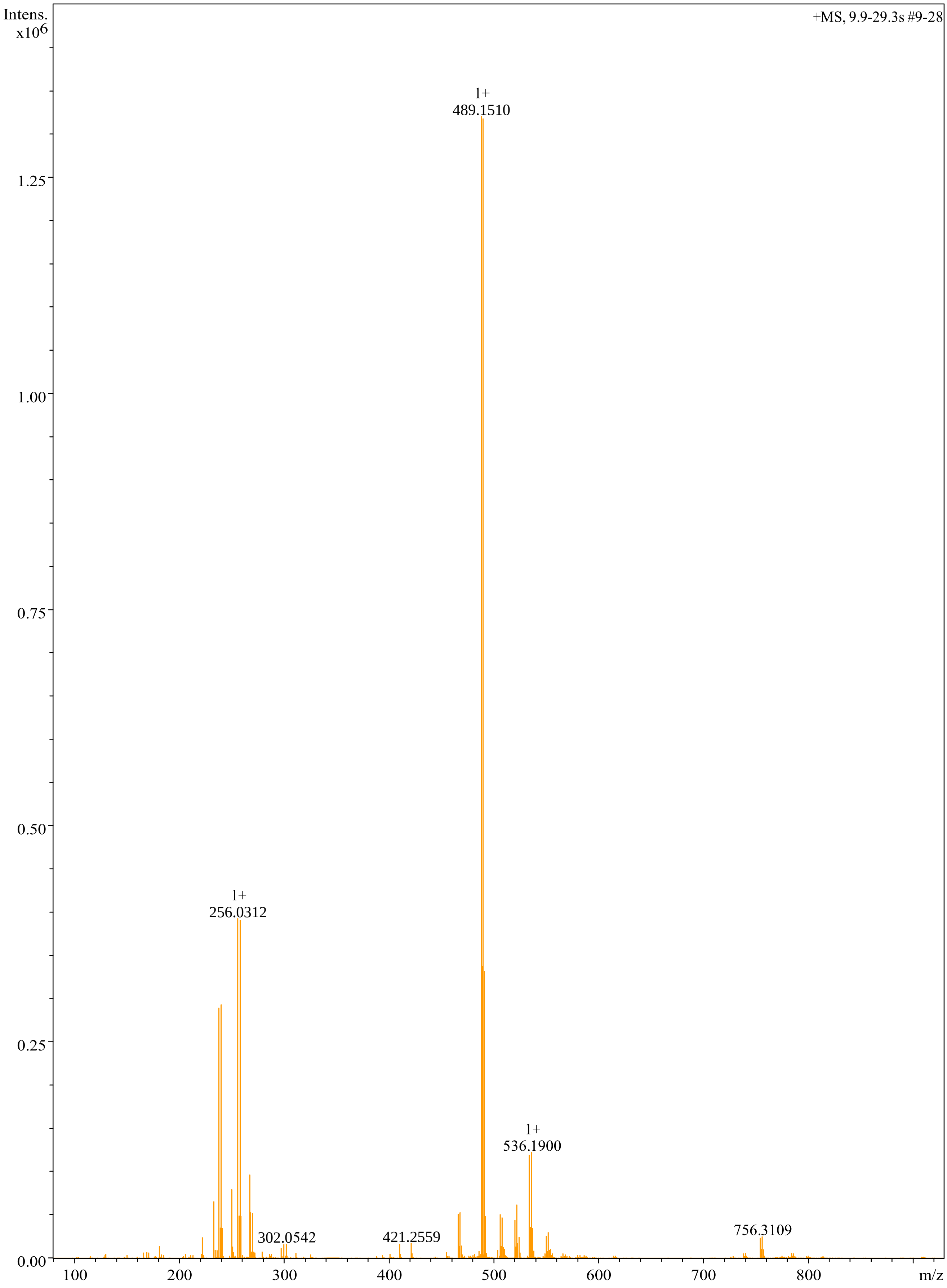

Window Display Report

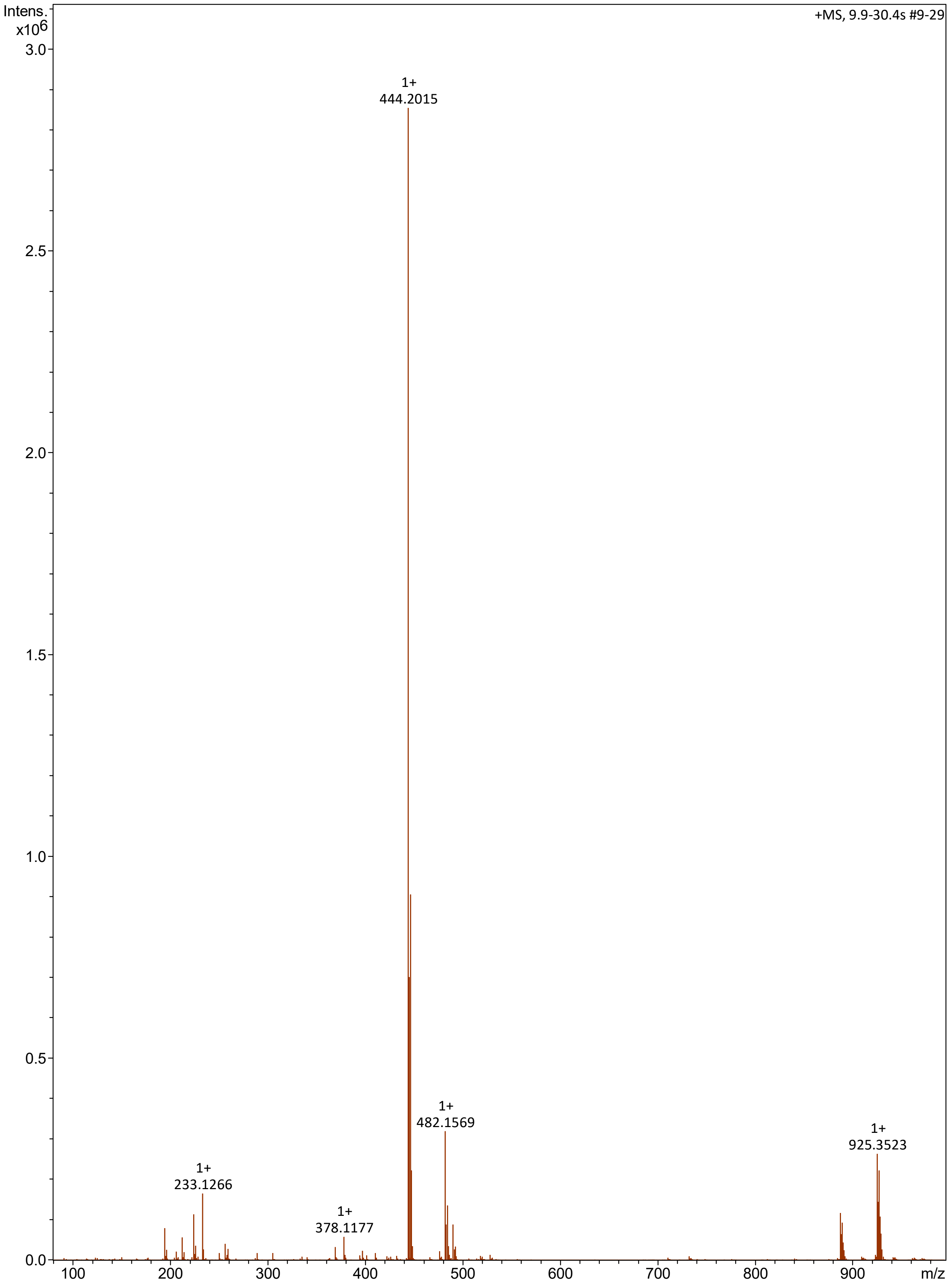

Window Display Report

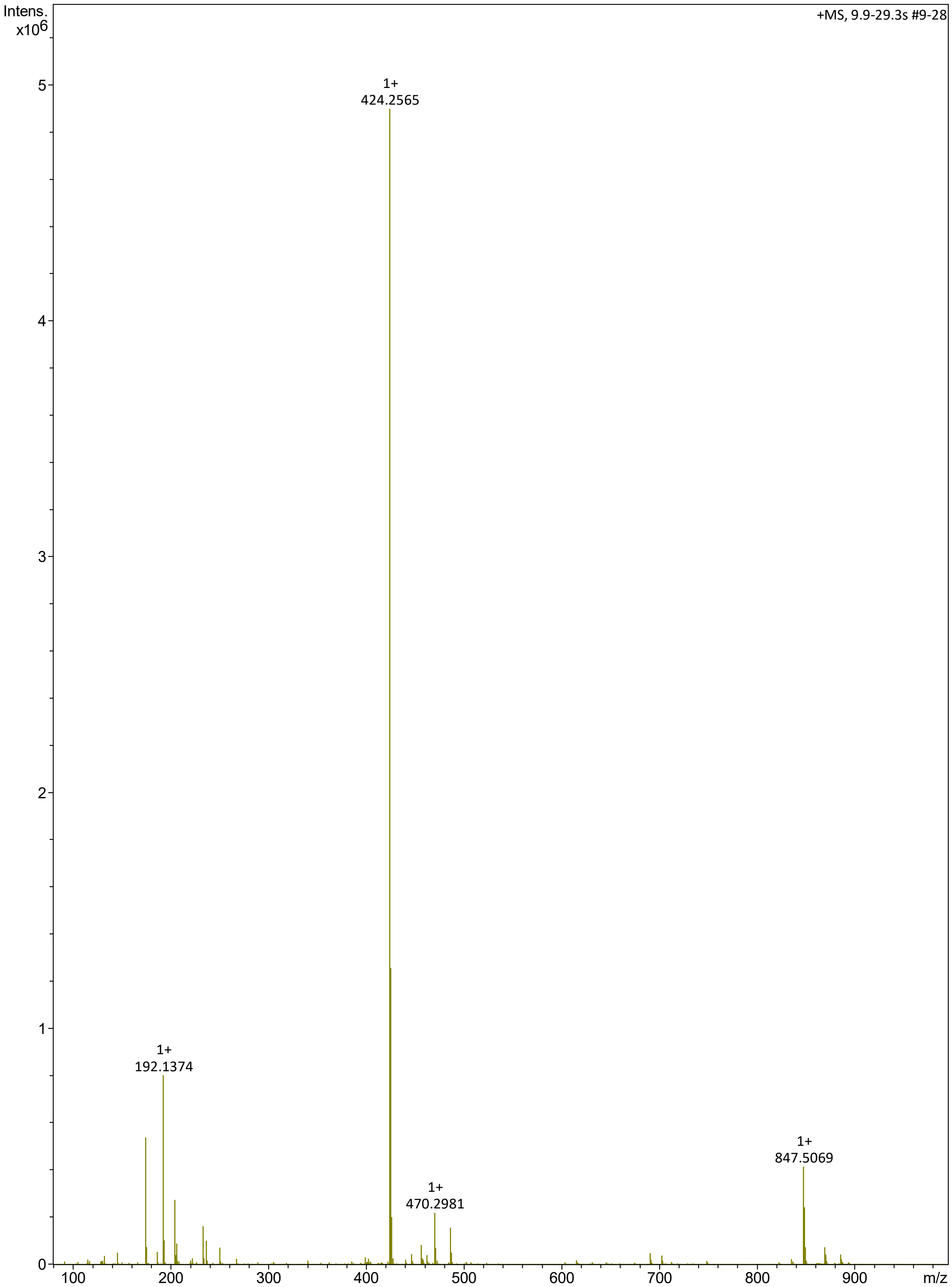

Supplement: Supplementary file 1 [file ijms-22-01410-s001.zip › MS.pdf]
