# Supplementary material for: Design and Synthesis of N-Substituted 3,4-Pyrroledicarboximides as Potential Anti-Inflammatory Agents
Source: Int J Mol Sci. 2021 Jan 30;22(3):1410. doi: 10.3390/ijms22031410 (PMC7866801; doi:10.3390/ijms22031410)
Supplement: Supplementary file 1 [file ijms-22-01410-s001.zip › Spectra H,C (2i-2p).pdf]

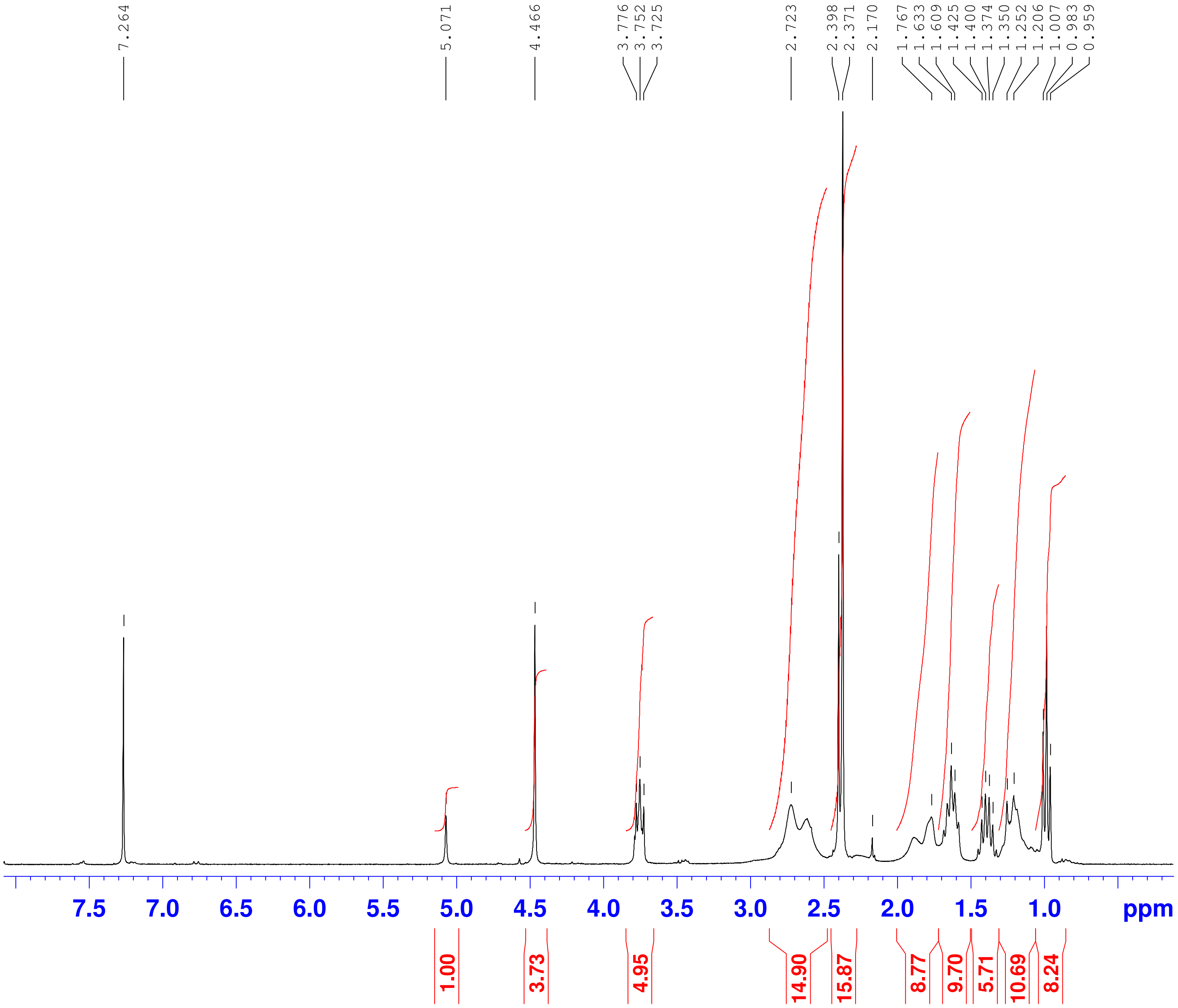

Current Data Parameters
NAME Paciorkowska B
EXPNO 4775
PROCNO 1904

F2 - Acquisition Parameters
Date_ 20180514
Time 10.43
INSTRUM spect
PROBHD 5 mm BBI 1H/D-
PULPROG zg30
TD 65536
SOLVENT CDCl3
NS 16
DS 0
SWH 6172.839 Hz
FIDRES 0.094190 Hz
AQ 5.3084660 sec
RG 80.6
DW 81.000 usec
DE 8.00 usec
TE 298.1 K
D1 1.00000000 sec
TD0 1

======== CHANNEL f1 ========
NUC1 1H
P1 10.40 usec
PL1 2.00 dB
SFO1 300.1518535 MHz

F2 - Processing parameters
SI 32768
SF 300.1500045 MHz
WDW EM
SSB 0
LB 0.30 Hz
GB 0
PC 20.00

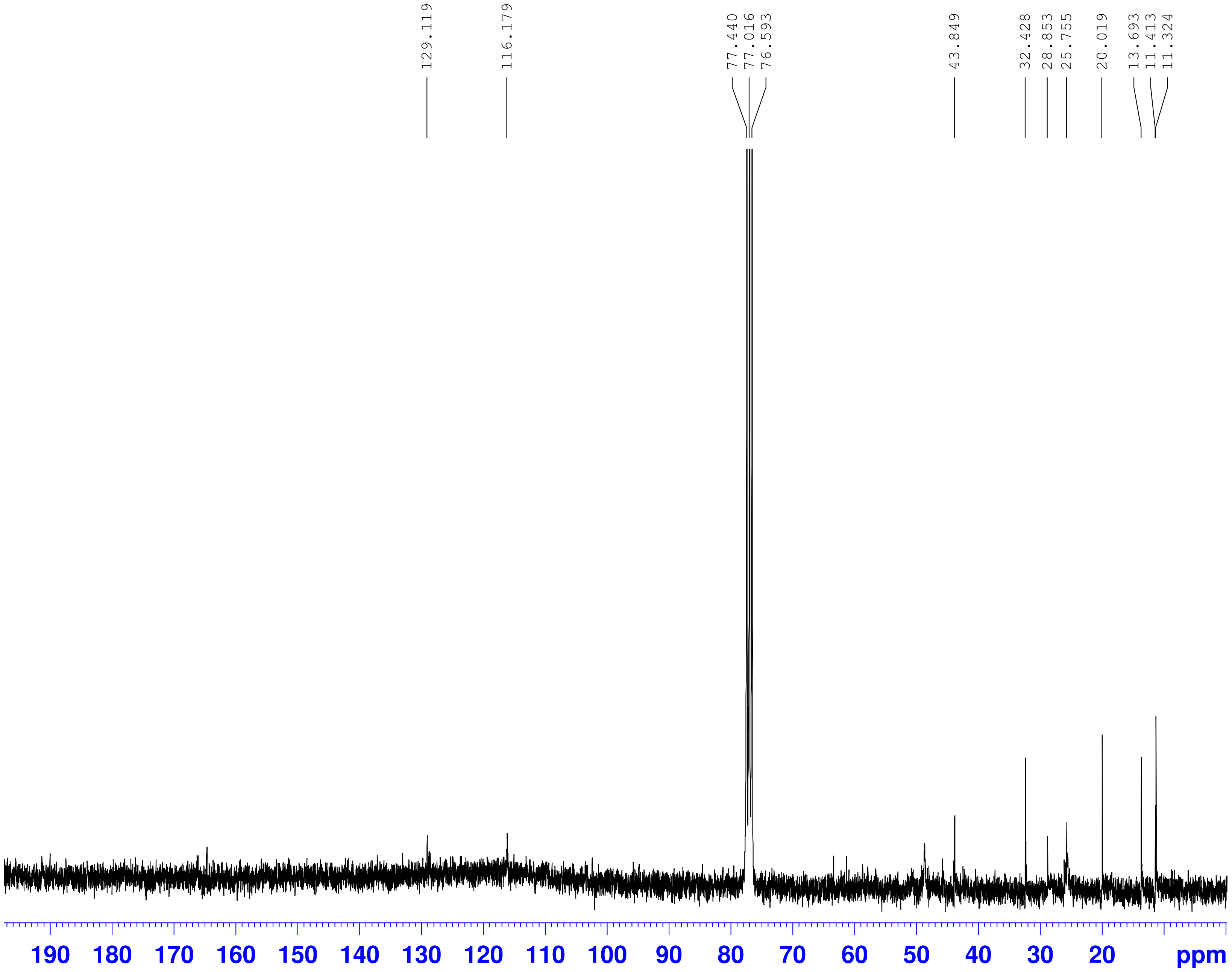

BRUKER

```
Current Data Parameters
NAME              redzicka
EXPNO                 6375
PROCNO                   1

F2 - Acquisition Parameters
Date_             20210120
Time                 22.02
INSTRUM              spect
PROBHD     5 mm BBI 1H/D-
PULPROG            zgpg30
TD                   65536
SOLVENT              CDCl3
NS                    5120
DS                       2
SWH              17985.611 Hz
FIDRES            0.274439 Hz
AQ               1.8219508 sec
RG                   26008
DW                  27.800 usec
DE                   20.00 usec
TE                   296.5 K
D1              2.00000000 sec
d11             0.03000000 sec
DELTA           1.89999998 sec
TD0                      1

======== CHANNEL f1 ========
NUC1                   13C
P1                   11.90 usec
PL1                  -6.00 dB
SFO1            75.4803248 MHz

======== CHANNEL f2 ========
CPDPRG2            waltz16
NUC2                    1H
PCPD2               100.00 usec
PL2                   2.00 dB
PL12                 21.66 dB
PL13                 23.00 dB
SFO2           300.1512006 MHz

F2 - Processing parameters
SI                   32768
SF              75.4727782 MHz
WDW                     EM
SSB                      0
LB                    1.00 Hz
GB                       0
PC                    2.00
```

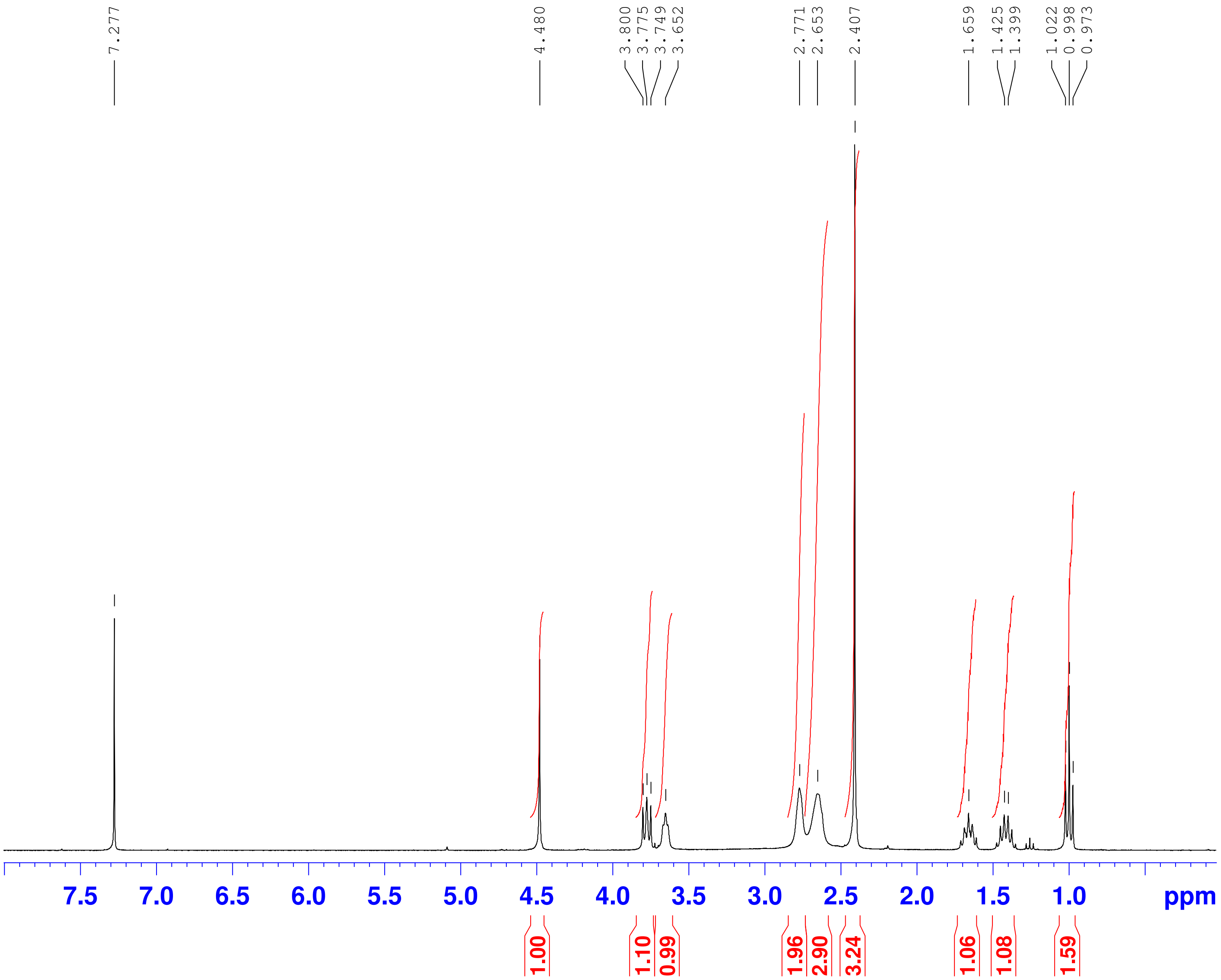

Current Data Parameters	
NAME	Redzicka 6103
EXPNO	1
PROCNO	1
**F2 - Acquisition Parameters**	
Date_	20200128
Time	9.10
INSTRUM	spect
PROBHD	5 mm BBI 1H/D-
PULPROG	zg30
TD	65536
SOLVENT	CDCl3
NS	16
DS	0
SWH	6172.839 Hz
FIDRES	0.094190 Hz
AQ	5.3084660 sec
RG	456.1
DW	81.000 usec
DE	8.00 usec
TE	298.0 K
D1	1.00000000 sec
TD0	1
**======== CHANNEL f1 ========**	
NUC1	1H
P1	11.00 usec
PL1	2.50 dB
SFO1	300.1518535 MHz
**F2 - Processing parameters**	
SI	32768
SF	300.1500000 MHz
WDW	EM
SSB	0
LB	0.30 Hz
GB	0
PC	20.00

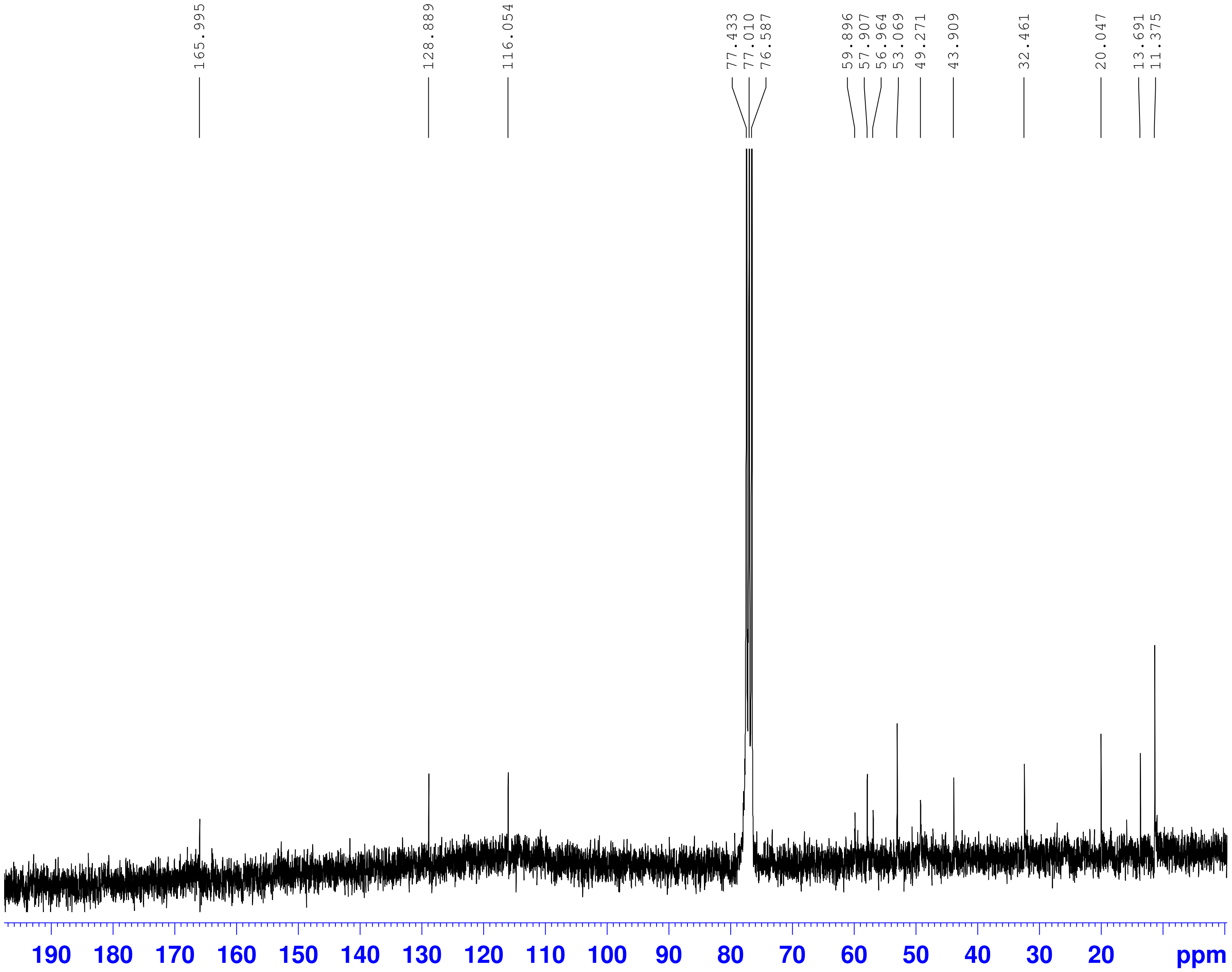

BRUKER

Current Data Parameters
NAME Redzicka 6252
EXPNO 1
PROCNO 1

F2 - Acquisition Parameters
Date_ 20201214
Time 23.13
INSTRUM spect
PROBHD 5 mm BBI 1H/D-
PULPROG zgpg30
TD 65536
SOLVENT CDCl3
NS 5120
DS 2
SWH 17985.611 Hz
FIDRES 0.274439 Hz
AQ 1.8219508 sec
RG 14596.5
DW 27.800 usec
DE 20.00 usec
TE 373.1 K
D1 2.00000000 sec
d11 0.03000000 sec
DELTA 1.89999998 sec
TD0 1

======== CHANNEL f1 ========
NUC1 13C
P1 11.90 usec
PL1 -6.00 dB
SFO1 75.4803248 MHz

======== CHANNEL f2 ========
CPDPRG2 waltz16
NUC2 1H
PCPD2 100.00 usec
PL2 2.00 dB
PL12 21.66 dB
PL13 23.00 dB
SFO2 300.1512006 MHz

F2 - Processing parameters
SI 32768
SF 75.4727782 MHz
WDW EM
SSB 0
LB 1.00 Hz
GB 0
PC 2.00

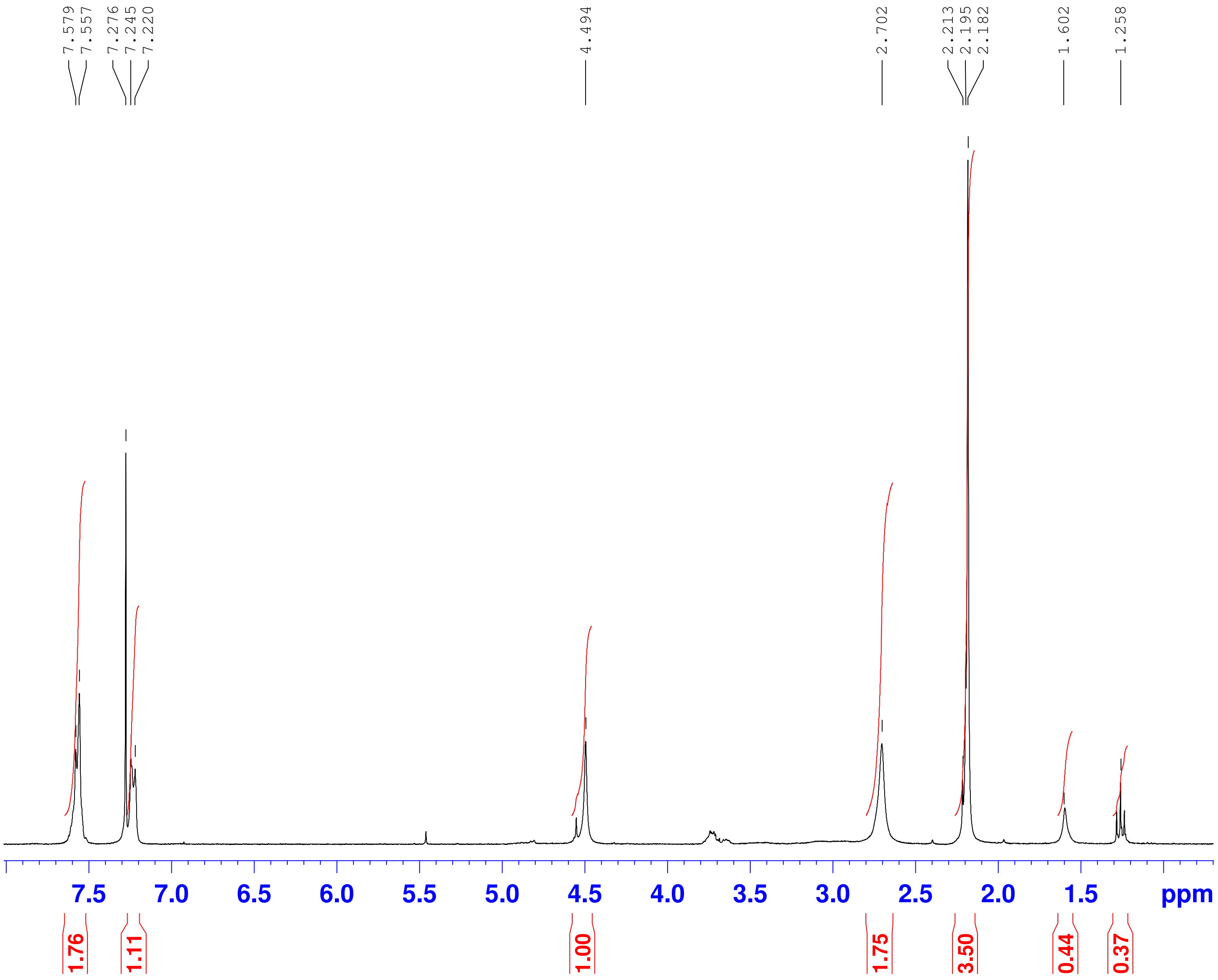


```
Current Data Parameters
NAME        Redzicka 6099
EXPNO                   1
PROCNO                  1

F2 - Acquisition Parameters
Date_            20200127
Time                10.11
INSTRUM             spect
PROBHD     5 mm BBI 1H/D-
PULPROG              zg30
TD                  65536
SOLVENT             CDCl3
NS                     16
DS                      0
SWH              6172.839 Hz
FIDRES           0.094190 Hz
AQ              5.3084660 sec
RG                  574.7
DW                 81.000 usec
DE                   8.00 usec
TE                  297.9 K
D1             1.00000000 sec
TD0                     1

======== CHANNEL f1 ========
NUC1                   1H
P1                  11.00 usec
PL1                  2.50 dB
SFO1          300.1518535 MHz

F2 - Processing parameters
SI                  32768
SF            300.1500000 MHz
WDW                    EM
SSB                     0
LB                   0.30 Hz
GB                      0
PC                  20.00
```

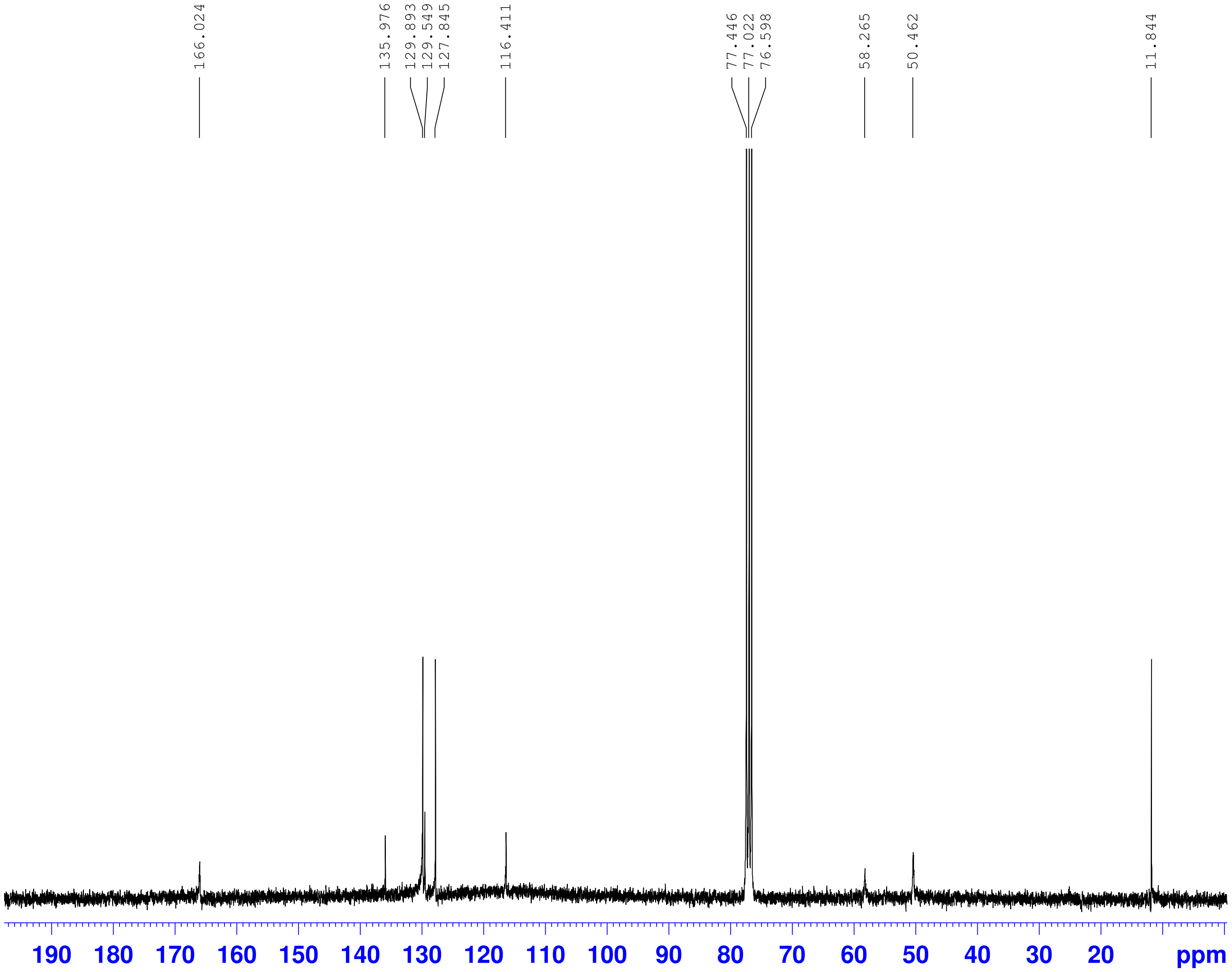

Current Data Parameters
NAME Redzicka 6320
EXPNO 1
PROCNO 1

F2 - Acquisition Parameters
Date_ 20201216
Time 21.27
INSTRUM spect
PROBHD 5 mm BBI 1H/D-
PULPROG zgpg30
TD 65536
SOLVENT CDCl3
NS 5120
DS 2
SWH 17985.611 Hz
FIDRES 0.274439 Hz
AQ 1.8219508 sec
RG 11585.2
DW 27.800 usec
DE 20.00 usec
TE 373.1 K
D1 2.00000000 sec
d11 0.03000000 sec
DELTA 1.89999998 sec
TD0 1

======== CHANNEL f1 ========
NUC1 13C
P1 11.90 usec
PL1 -6.00 dB
SFO1 75.4803248 MHz

======== CHANNEL f2 ========
CPDPRG2 waltz16
NUC2 1H
PCPD2 100.00 usec
PL2 2.00 dB
PL12 21.66 dB
PL13 23.00 dB
SFO2 300.1512006 MHz

F2 - Processing parameters
SI 32768
SF 75.4727782 MHz
WDW EM
SSB 0
LB 1.00 Hz
GB 0
PC 2.00

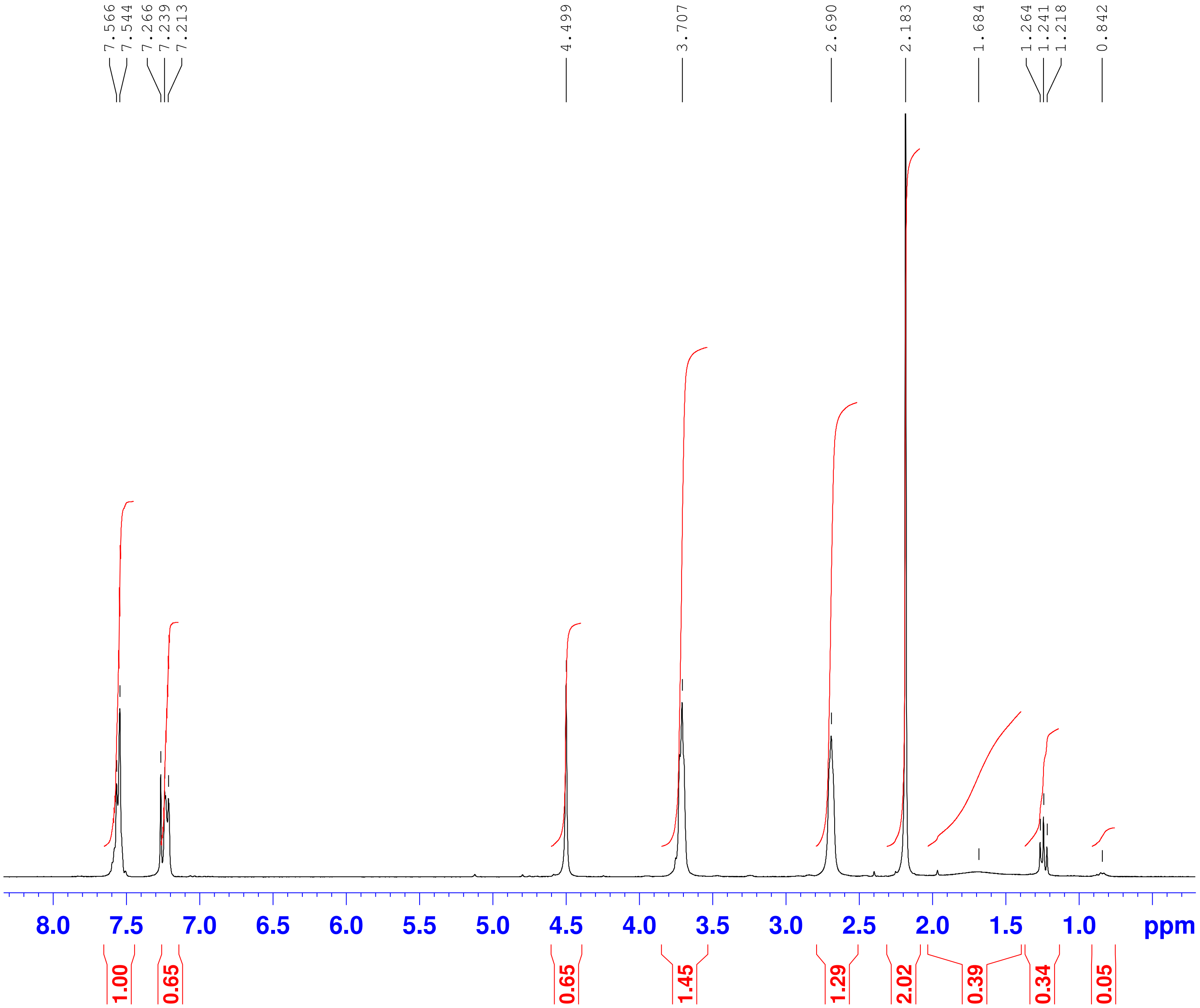


```
Current Data Parameters
NAME       Paciorkowska B
EXPNO                4799
PROCNO               2103

F2 - Acquisition Parameters
Date_            20180604
Time                14.30
INSTRUM             spect
PROBHD     5 mm BBI 1H/D-
PULPROG              zg30
TD                  65536
SOLVENT             CDCl3
NS                     16
DS                      0
SWH              6172.839 Hz
FIDRES           0.094190 Hz
AQ              5.3084660 sec
RG                   80.6
DW                 81.000 usec
DE                   8.00 usec
TE                  298.8 K
D1             1.00000000 sec
TD0                     1

======== CHANNEL f1 ========
NUC1                   1H
P1                  10.40 usec
PL1                  2.00 dB
SFO1          300.1518535 MHz

F2 - Processing parameters
SI                  32768
SF            300.1500045 MHz
WDW                    EM
SSB                     0
LB                   0.30 Hz
GB                      0
PC                  20.00
```

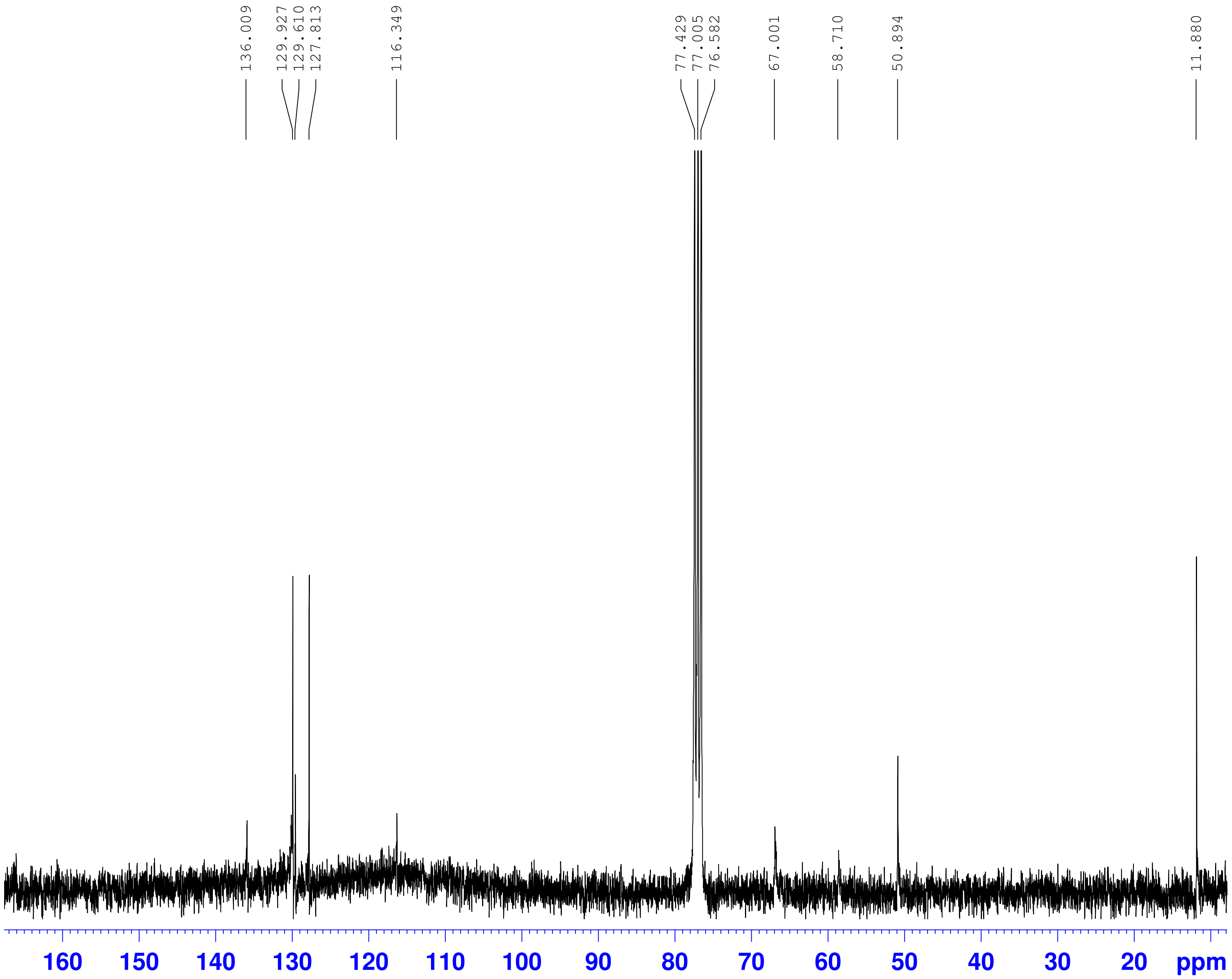

BRUKER

```
Current Data Parameters
NAME      Redzicka 6104
EXPNO                 2
PROCNO                1

F2 - Acquisition Parameters
Date_          20200128
Time              22.15
INSTRUM           spect
PROBHD   5 mm BBI 1H/D-
PULPROG         zgpg30
TD               65536
SOLVENT          CDCl3
NS                5120
DS                   2
SWH          17985.611 Hz
FIDRES        0.274439 Hz
AQ           1.8219508 sec
RG             14596.5
DW              27.800 usec
DE               20.00 usec
TE               298.9 K
D1          2.00000000 sec
d11         0.03000000 sec
DELTA       1.89999998 sec
TD0                  1

======== CHANNEL f1 ========
NUC1               13C
P1               11.00 usec
PL1              -6.00 dB
SFO1        75.4803248 MHz

======== CHANNEL f2 ========
CPDPRG2        waltz16
NUC2                1H
PCPD2           100.00 usec
PL2               2.00 dB
PL12             21.45 dB
PL13             23.00 dB
SFO2       300.1512006 MHz

F2 - Processing parameters
SI               32768
SF          75.4727780 MHz
WDW                 EM
SSB                  0
LB                1.00 Hz
GB                   0
PC                2.00
```

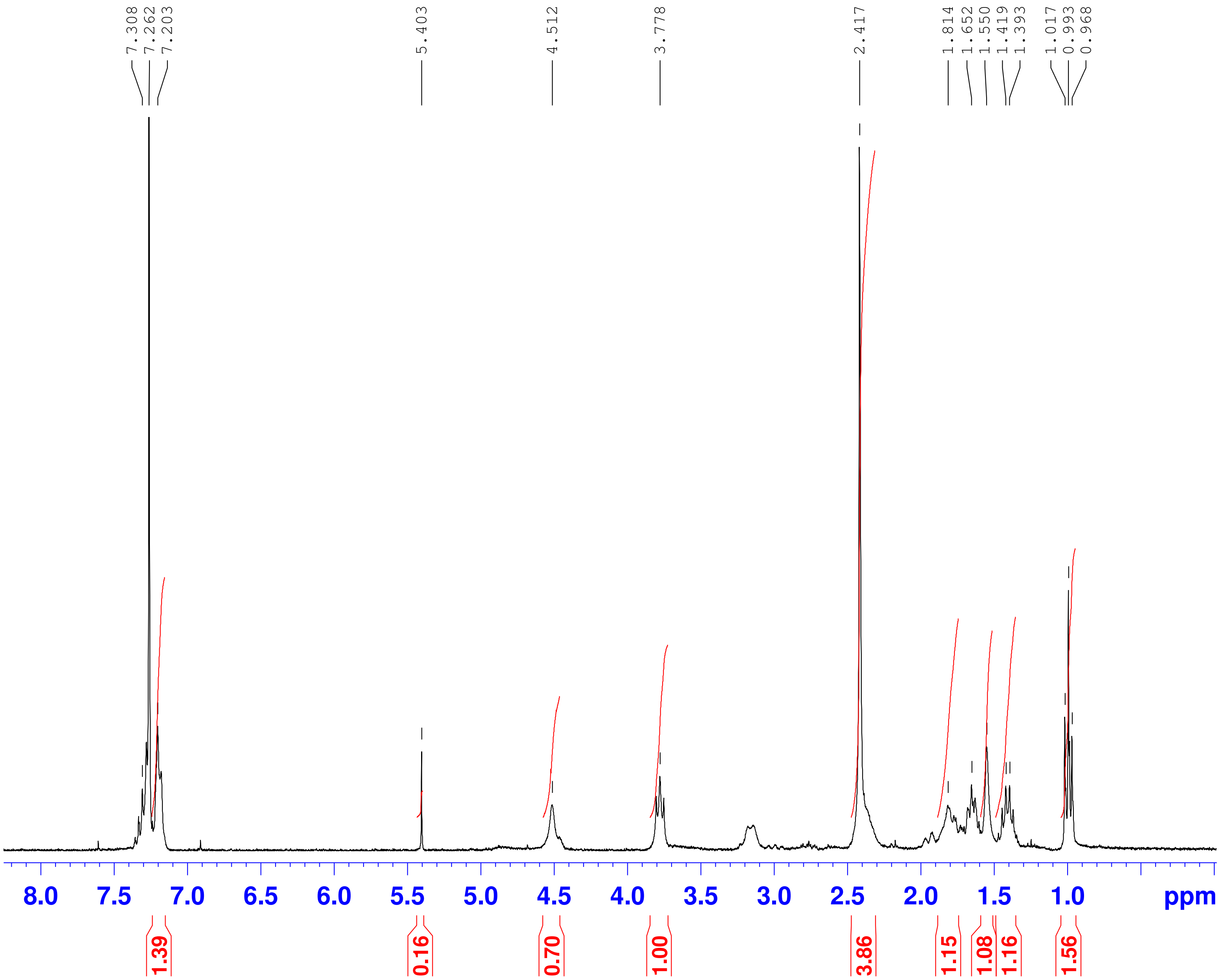

Current Data Parameters

NAME	redzicka 6092
EXPNO	1
PROCNO	1

F2 - Acquisition Parameters

Date_	20191218
Time	10.54
INSTRUM	spect
PROBHD	5 mm BBI 1H/D-
PULPROG	zg30
TD	65536
SOLVENT	CDCl3
NS	16
DS	0
SWH	6172.839 Hz
FIDRES	0.094190 Hz
AQ	5.3084660 sec
RG	724.1
DW	81.000 usec
DE	8.00 usec
TE	297.5 K
D1	1.00000000 sec
TD0	1

======== CHANNEL f1 ========

NUC1	1H
P1	10.40 usec
PL1	2.00 dB
SFO1	300.1518535 MHz

F2 - Processing parameters

SI	32768
SF	300.1500045 MHz
WDW	EM
SSB	0
LB	0.30 Hz
GB	0
PC	10.00

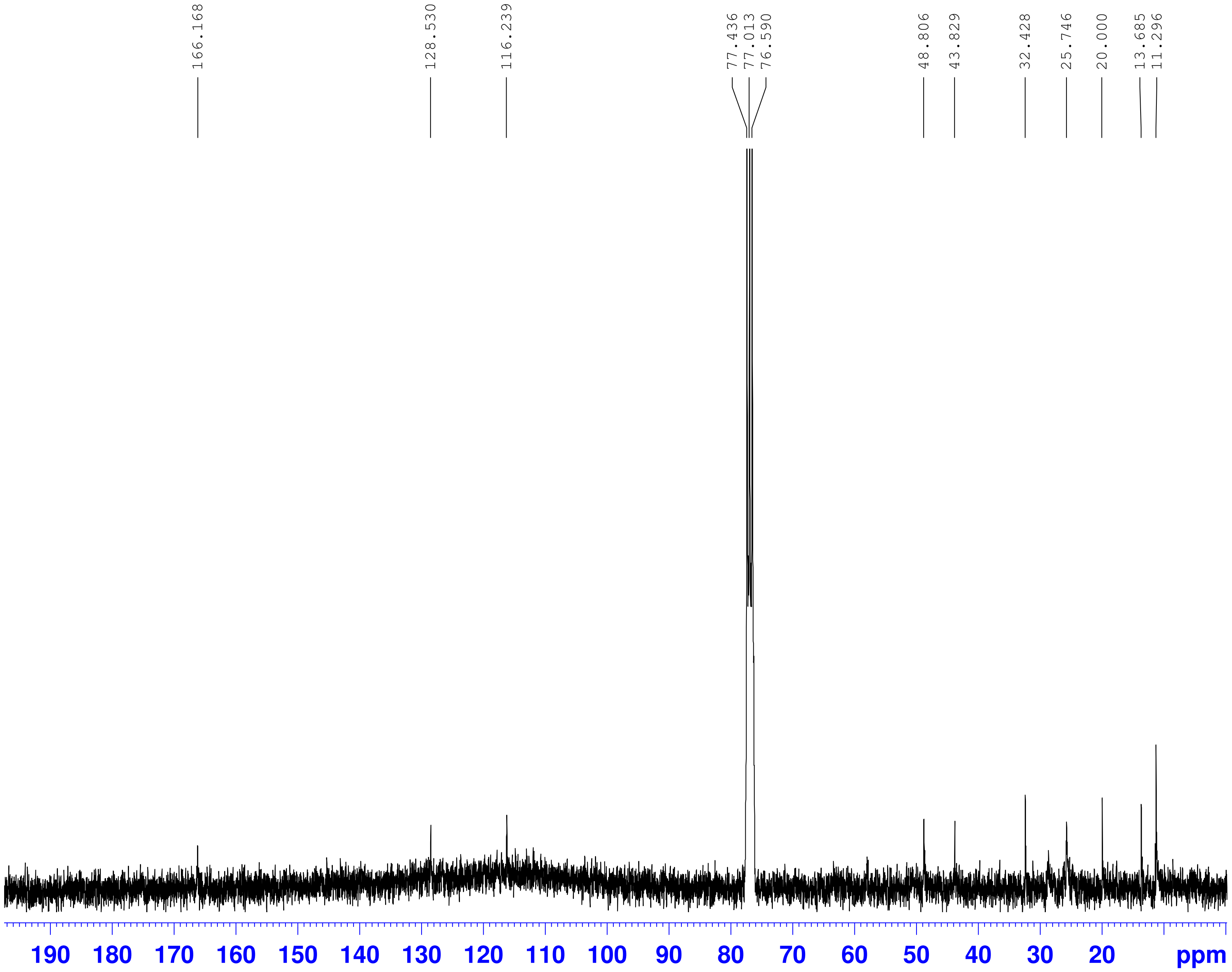

Current Data Parameters	
NAME	Redzicka 6250
EXPNO	1
PROCNO	1
F2 - Acquisition Parameters	
Date_	20201214
Time	15.24
INSTRUM	spect
PROBHD	5 mm BBI 1H/D-
PULPROG	zgpg30
TD	65536
SOLVENT	CDCl3
NS	5120
DS	2
SWH	17985.611 Hz
FIDRES	0.274439 Hz
AQ	1.8219508 sec
RG	11585.2
DW	27.800 usec
DE	20.00 usec
TE	373.1 K
D1	2.00000000 sec
d11	0.03000000 sec
DELTA	1.89999998 sec
TD0	1
======== CHANNEL f1 ========	
NUC1	13C
P1	11.90 usec
PL1	-6.00 dB
SFO1	75.4803248 MHz
======== CHANNEL f2 ========	
CPDPRG2	waltz16
NUC2	1H
PCPD2	100.00 usec
PL2	2.00 dB
PL12	21.66 dB
PL13	23.00 dB
SFO2	300.1512006 MHz
F2 - Processing parameters	
SI	32768
SF	75.4727782 MHz
WDW	EM
SSB	0
LB	1.00 Hz
GB	0
PC	2.00

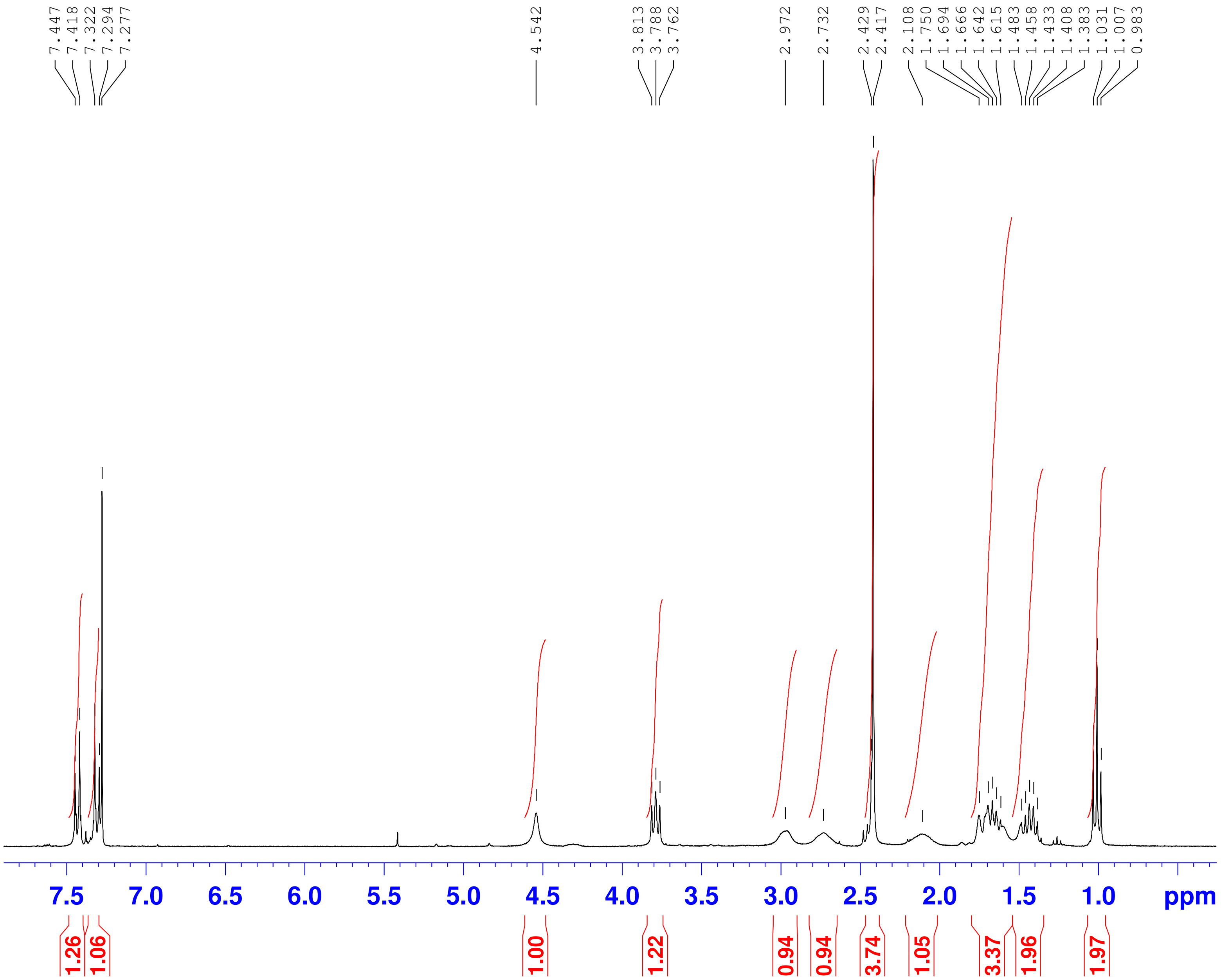

Current Data Parameters
NAME Redzicka 6102
EXPNO 1
PROCNO 1

F2 - Acquisition Parameters
Date_ 20200127
Time 16.52
INSTRUM spect
PROBHD 5 mm BBI 1H/D-
PULPROG zg30
TD 65536
SOLVENT CDCl3
NS 16
DS 0
SWH 6172.839 Hz
FIDRES 0.094190 Hz
AQ 5.3084660 sec
RG 512
DW 81.000 usec
DE 8.00 usec
TE 298.3 K
D1 1.00000000 sec
TD0 1

======== CHANNEL f1 ========
NUC1 1H
P1 11.00 usec
PL1 2.50 dB
SFO1 300.1518535 MHz

F2 - Processing parameters
SI 32768
SF 300.1500000 MHz
WDW EM
SSB 0
LB 0.30 Hz
GB 0
PC 10.00

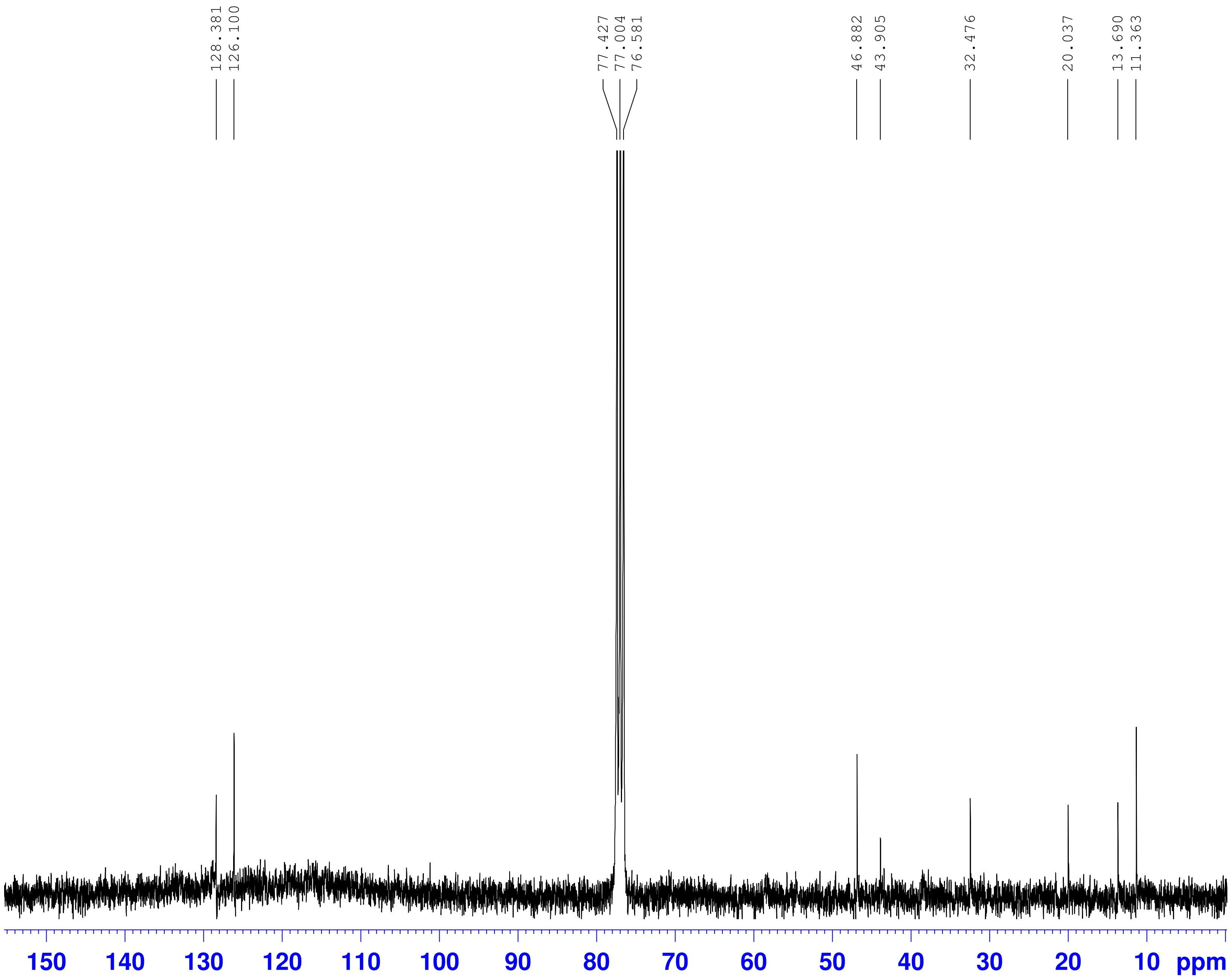

BRUKER

```
Current Data Parameters
NAME      Redzicka 6102
EXPNO                 2
PROCNO                1

F2 - Acquisition Parameters
Date_          20200127
Time              22.24
INSTRUM           spect
PROBHD   5 mm BBI 1H/D-
PULPROG         zgpg30
TD                65536
SOLVENT           CDCl3
NS                 5120
DS                    2
SWH           17985.611 Hz
FIDRES         0.274439 Hz
AQ            1.8219508 sec
RG              14596.5
DW               27.800 usec
DE                20.00 usec
TE                299.4 K
D1           2.00000000 sec
d11          0.03000000 sec
DELTA        1.89999998 sec
TD0                   1

======== CHANNEL f1 ========
NUC1                13C
P1                11.00 usec
PL1               -6.00 dB
SFO1         75.4803248 MHz

======== CHANNEL f2 ========
CPDPRG2         waltz16
NUC2                 1H
PCPD2            100.00 usec
PL2                2.00 dB
PL12              21.45 dB
PL13              23.00 dB
SFO2        300.1512006 MHz

F2 - Processing parameters
SI                32768
SF           75.4727780 MHz
WDW                  EM
SSB                   0
LB                 1.00 Hz
GB                    0
PC                 2.00
```

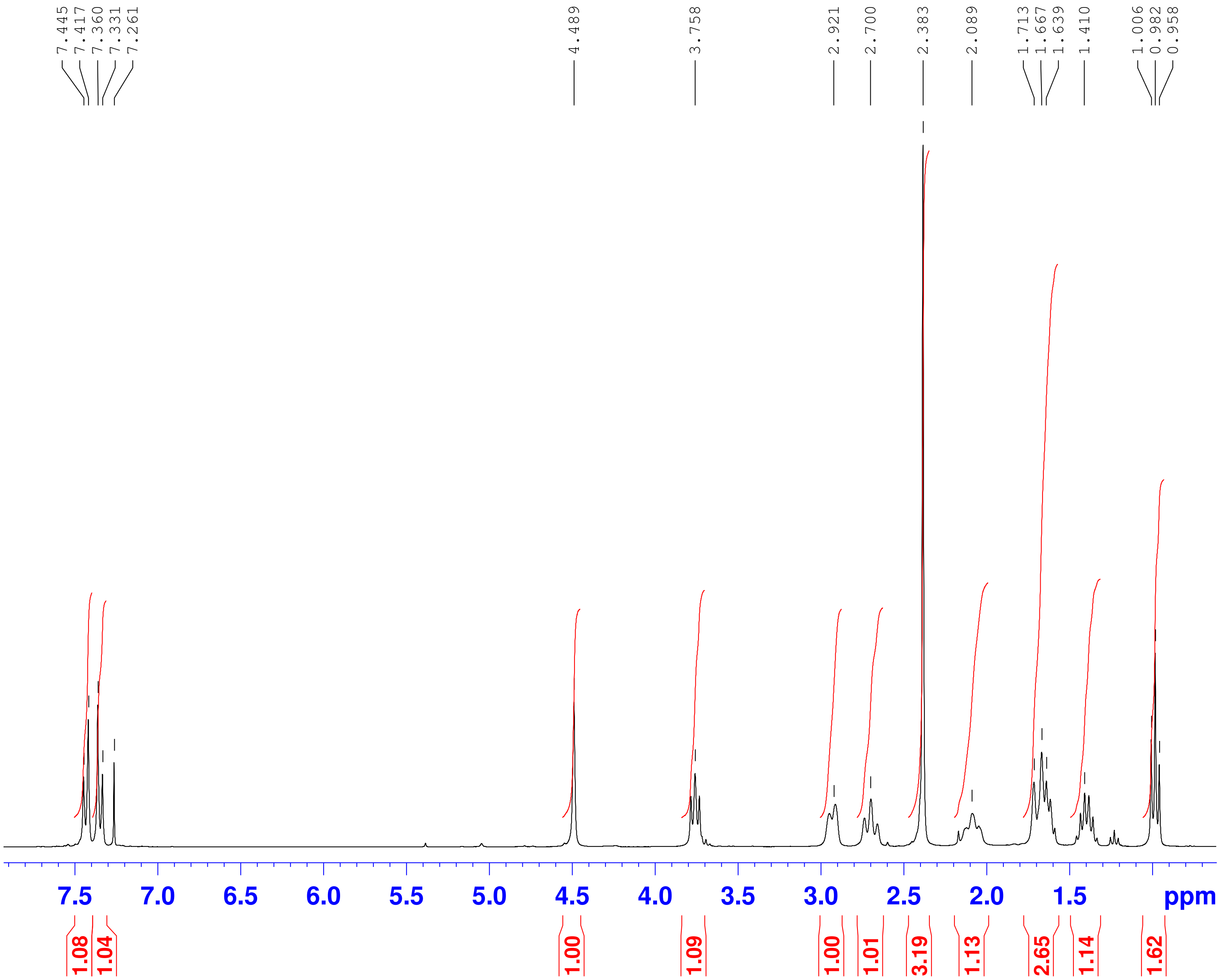


```
Current Data Parameters
NAME              redzicka
EXPNO                 6067
PROCNO                3328

F2 - Acquisition Parameters
Date_             20191106
Time                 14.13
INSTRUM              spect
PROBHD   5 mm BBI 1H/D-
PULPROG               zg30
TD                   65536
SOLVENT              CDCl3
NS                      16
DS                       0
SWH               6172.839 Hz
FIDRES            0.094190 Hz
AQ               5.3084660 sec
RG                     128
DW                  81.000 usec
DE                    8.00 usec
TE                   298.3 K
D1              1.00000000 sec
TD0                      1

======== CHANNEL f1 ========
NUC1                    1H
P1                   10.40 usec
PL1                   2.00 dB
SFO1           300.1518535 MHz

F2 - Processing parameters
SI                   32768
SF             300.1500045 MHz
WDW                     EM
SSB                      0
LB                    0.30 Hz
GB                       0
PC                   20.00
```

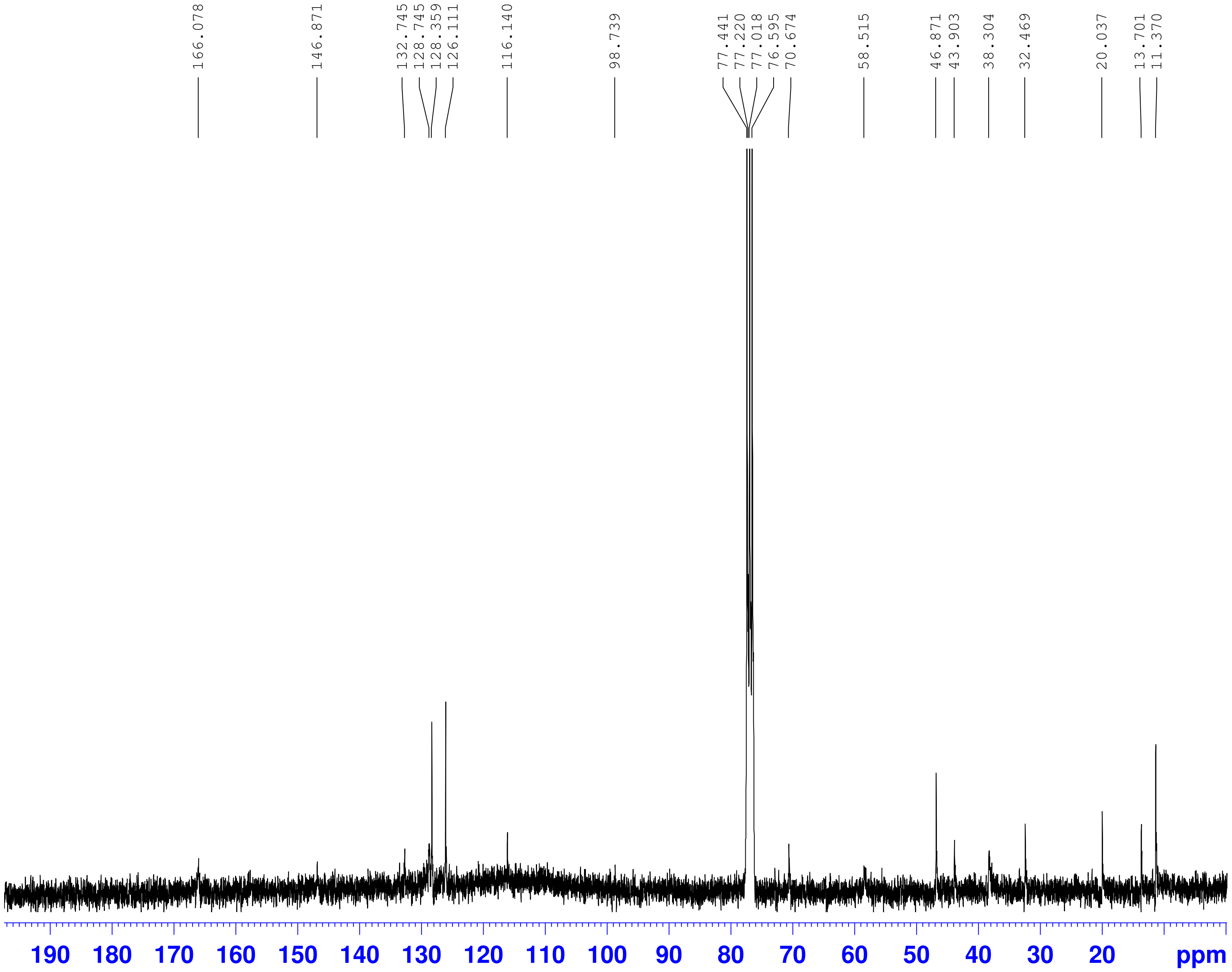

BRUKER

Current Data Parameters
NAME Redzicka 4595
EXPNO 1
PROCNO 1

F2 - Acquisition Parameters
Date_ 20201215
Time 14.11
INSTRUM spect
PROBHD 5 mm BBI 1H/D-
PULPROG zgpg30
TD 65536
SOLVENT CDCl3
NS 5120
DS 2
SWH 17985.611 Hz
FIDRES 0.274439 Hz
AQ 1.8219508 sec
RG 11585.2
DW 27.800 usec
DE 20.00 usec
TE 373.1 K
D1 2.00000000 sec
d11 0.03000000 sec
DELTA 1.89999998 sec
TD0 1

======== CHANNEL f1 ========
NUC1 13C
P1 11.90 usec
PL1 -6.00 dB
SFO1 75.4803248 MHz

======== CHANNEL f2 ========
CPDPRG2 waltz16
NUC2 1H
PCPD2 100.00 usec
PL2 2.00 dB
PL12 21.66 dB
PL13 23.00 dB
SFO2 300.1512006 MHz

F2 - Processing parameters
SI 32768
SF 75.4727782 MHz
WDW EM
SSB 0
LB 1.00 Hz
GB 0
PC 1.60

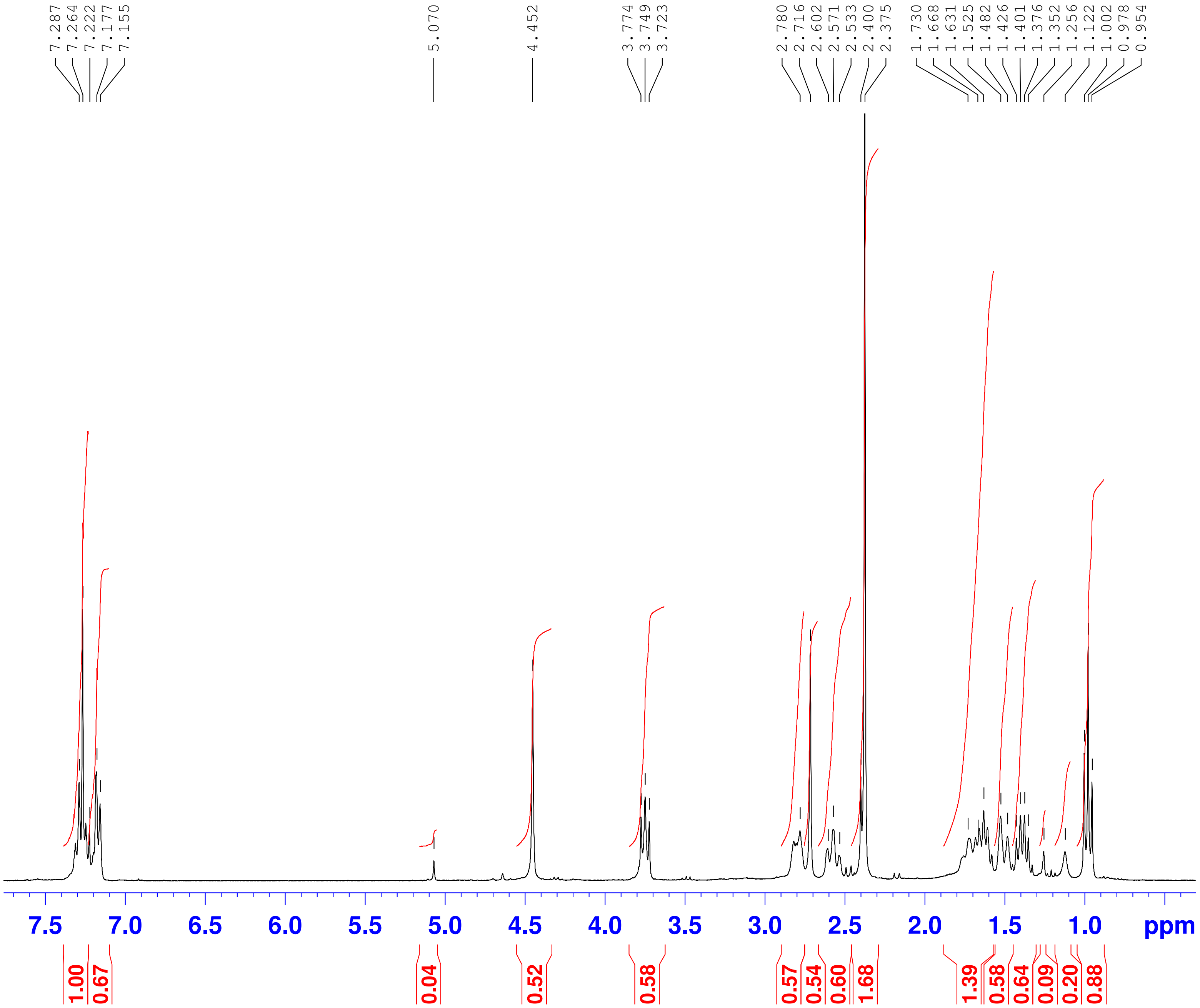

Current Data Parameters
NAME Paciorkowska B
EXPNO 4791
PROCNO 1988

F2 - Acquisition Parameters
Date_ 20180521
Time 13.07
INSTRUM spect
PROBHD 5 mm BBI 1H/D-
PULPROG zg30
TD 65536
SOLVENT CDCl3
NS 16
DS 0
SWH 6172.839 Hz
FIDRES 0.094190 Hz
AQ 5.3084660 sec
RG 80.6
DW 81.000 usec
DE 8.00 usec
TE 298.2 K
D1 1.00000000 sec
TD0 1

======== CHANNEL f1 ========
NUC1 1H
P1 10.40 usec
PL1 2.00 dB
SFO1 300.1518535 MHz

F2 - Processing parameters
SI 32768
SF 300.1500045 MHz
WDW EM
SSB 0
LB 0.30 Hz
GB 0
PC 20.00

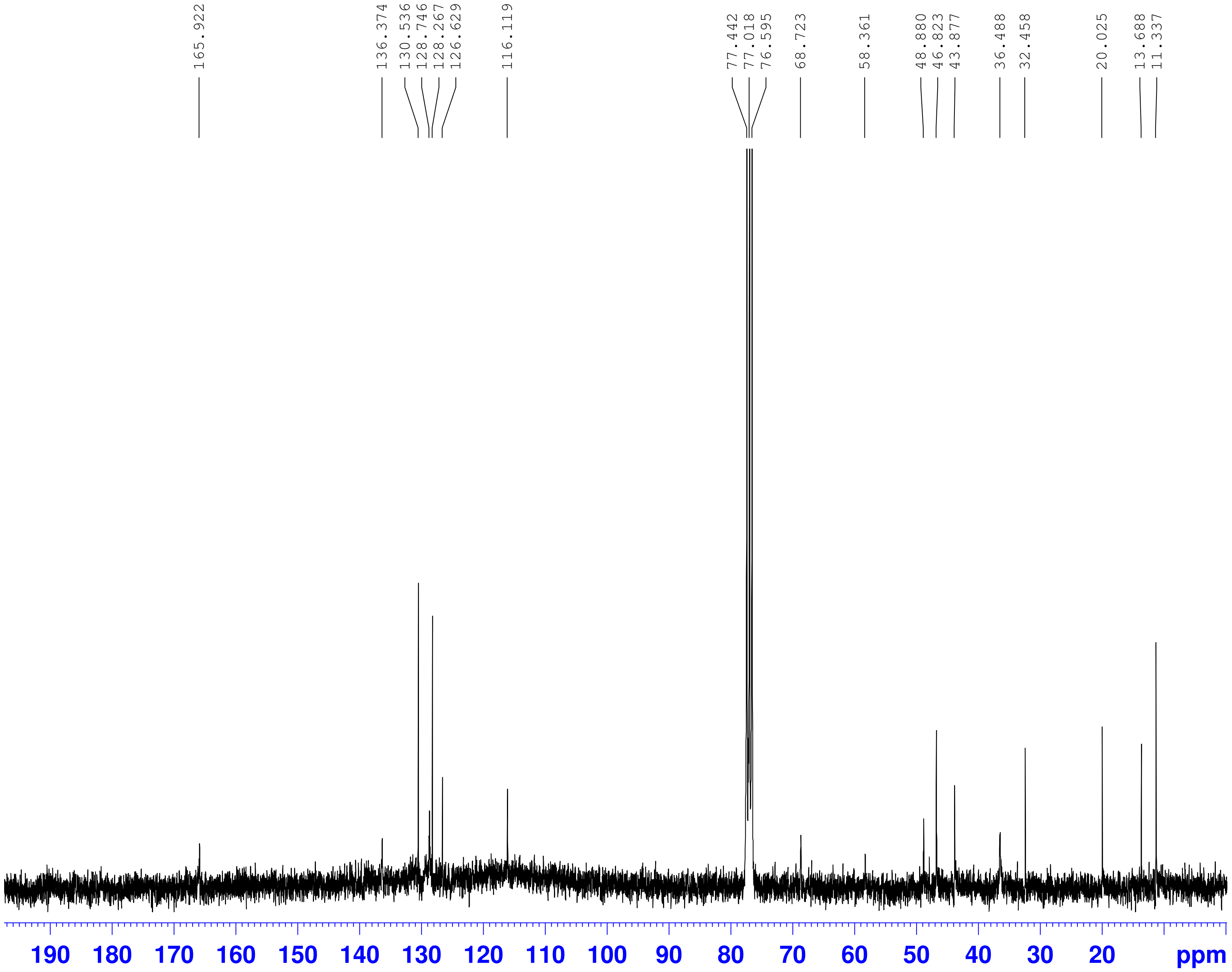

Current Data Parameters		
NAME	Redzicka 6316	
EXPNO	1	
PROCNO	1	
F2 - Acquisition Parameters		
Date_	20201216	
Time	15.03	
INSTRUM	spect	
PROBHD	5 mm BBI 1H/D-	
PULPROG	zgpg30	
TD	65536	
SOLVENT	CDCl3	
NS	5120	
DS	2	
SWH	17985.611	Hz
FIDRES	0.274439	Hz
AQ	1.8219508	sec
RG	16384	
DW	27.800	usec
DE	20.00	usec
TE	373.1	K
D1	2.00000000	sec
d11	0.03000000	sec
DELTA	1.89999998	sec
TD0	1	
======== CHANNEL f1 ========		
NUC1	13C	
P1	11.90	usec
PL1	-6.00	dB
SFO1	75.4803248	MHz
======== CHANNEL f2 ========		
CPDPRG2	waltz16	
NUC2	1H	
PCPD2	100.00	usec
PL2	2.00	dB
PL12	21.66	dB
PL13	23.00	dB
SFO2	300.1512006	MHz
F2 - Processing parameters		
SI	32768	
SF	75.4727782	MHz
WDW	EM	
SSB	0	
LB	1.00	Hz
GB	0	
PC	2.00	
