# Supplementary material for: Design and Synthesis of N-Substituted 3,4-Pyrroledicarboximides as Potential Anti-Inflammatory Agents
Source: Int J Mol Sci. 2021 Jan 30;22(3):1410. doi: 10.3390/ijms22031410 (PMC7866801; doi:10.3390/ijms22031410)
Supplement: Supplementary file 1 [file ijms-22-01410-s001.zip › Spectra H,C (2a-2h).pdf]

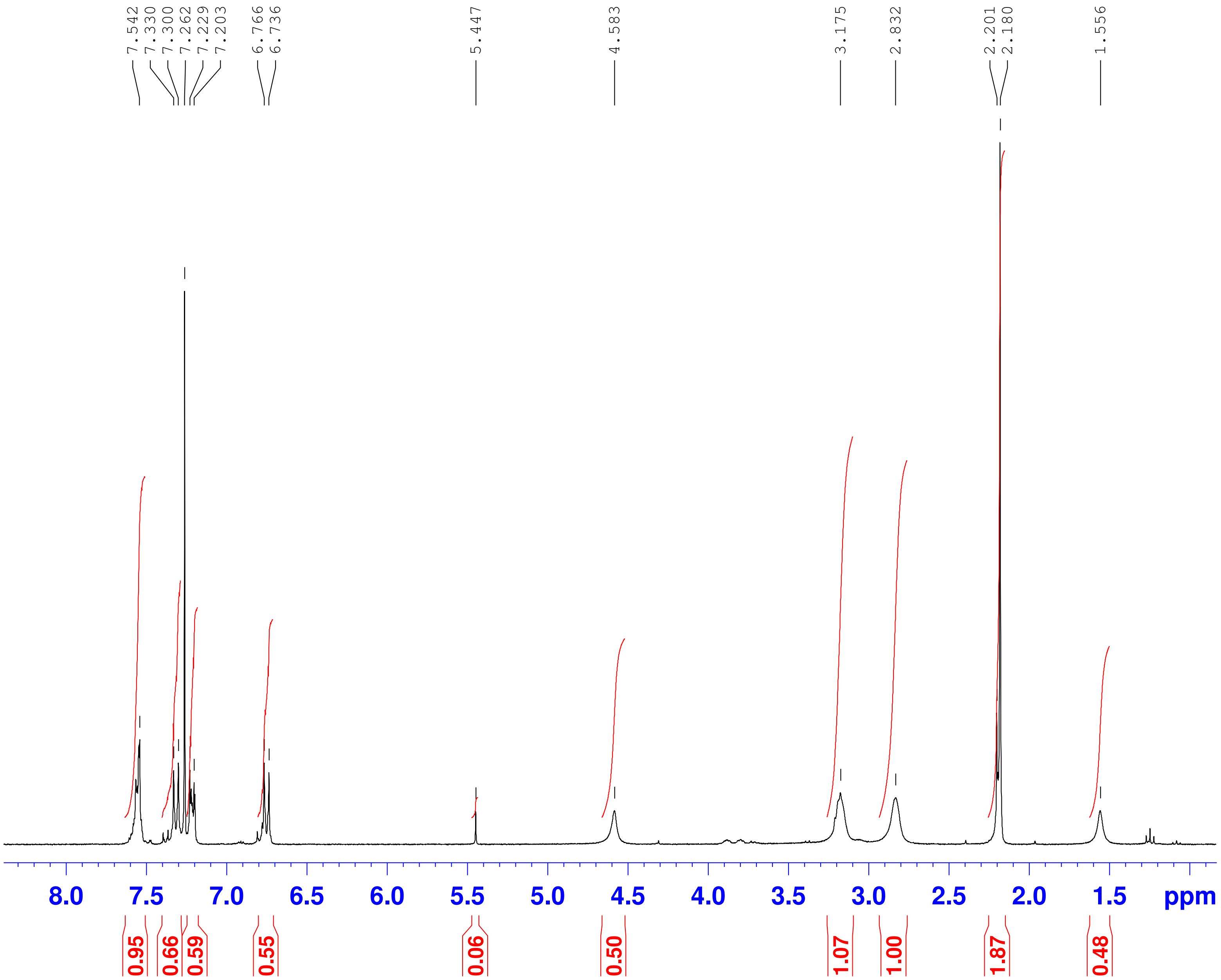

BRUKER

Current Data Parameters
NAME redzicka 6093
EXPNO 1
PROCNO 1

F2 - Acquisition Parameters
Date_ 20191218
Time 16.37
INSTRUM spect
PROBHD 5 mm BBI 1H/D-
PULPROG zg30
TD 65536
SOLVENT CDCl3
NS 16
DS 0
SWH 6172.839 Hz
FIDRES 0.094190 Hz
AQ 5.3084660 sec
RG 645.1
DW 81.000 usec
DE 8.00 usec
TE 298.0 K
D1 1.00000000 sec
TD0 1

======== CHANNEL f1 ========
NUC1 1H
P1 10.40 usec
PL1 2.00 dB
SFO1 300.1518535 MHz

F2 - Processing parameters
SI 32768
SF 300.1500045 MHz
WDW EM
SSB 0
LB 0.30 Hz
GB 0
PC 20.00

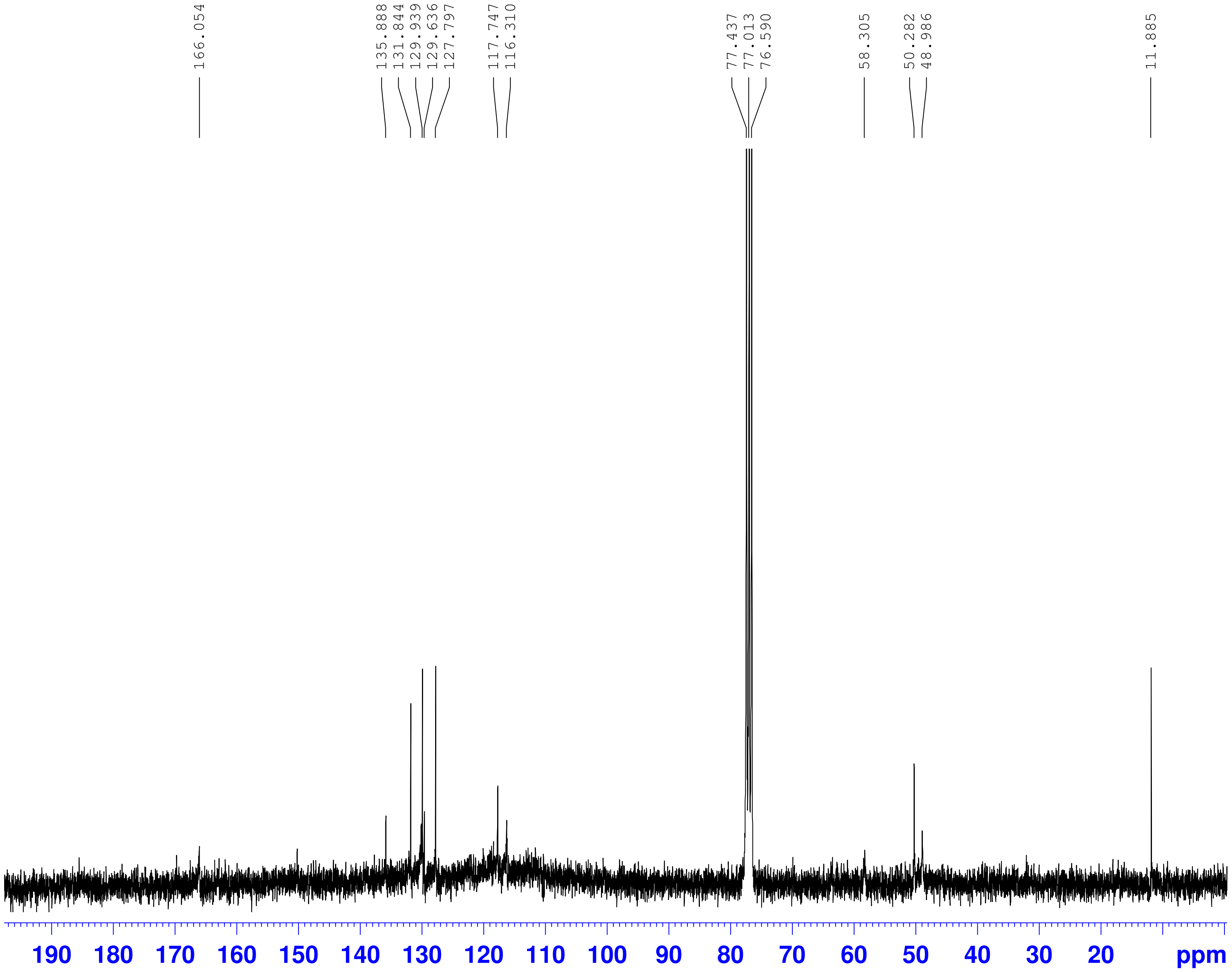


```
Current Data Parameters
NAME      Redzicka 6324
EXPNO                 1
PROCNO                1

F2 - Acquisition Parameters
Date_          20201217
Time              14.31
INSTRUM           spect
PROBHD   5 mm BBI 1H/D-
PULPROG         zgpg30
TD               65536
SOLVENT          CDCl3
NS                5120
DS                   2
SWH          17985.611 Hz
FIDRES        0.274439 Hz
AQ           1.8219508 sec
RG                8192
DW              27.800 usec
DE               20.00 usec
TE               298.3 K
D1          2.00000000 sec
d11         0.03000000 sec
DELTA       1.89999998 sec
TD0                  1

======== CHANNEL f1 ========
NUC1               13C
P1               11.90 usec
PL1              -6.00 dB
SFO1        75.4803248 MHz

======== CHANNEL f2 ========
CPDPRG2        waltz16
NUC2                1H
PCPD2           100.00 usec
PL2               2.00 dB
PL12             21.66 dB
PL13             23.00 dB
SFO2       300.1512006 MHz

F2 - Processing parameters
SI               32768
SF          75.4727782 MHz
WDW                 EM
SSB                  0
LB                1.00 Hz
GB                   0
PC                2.00
```

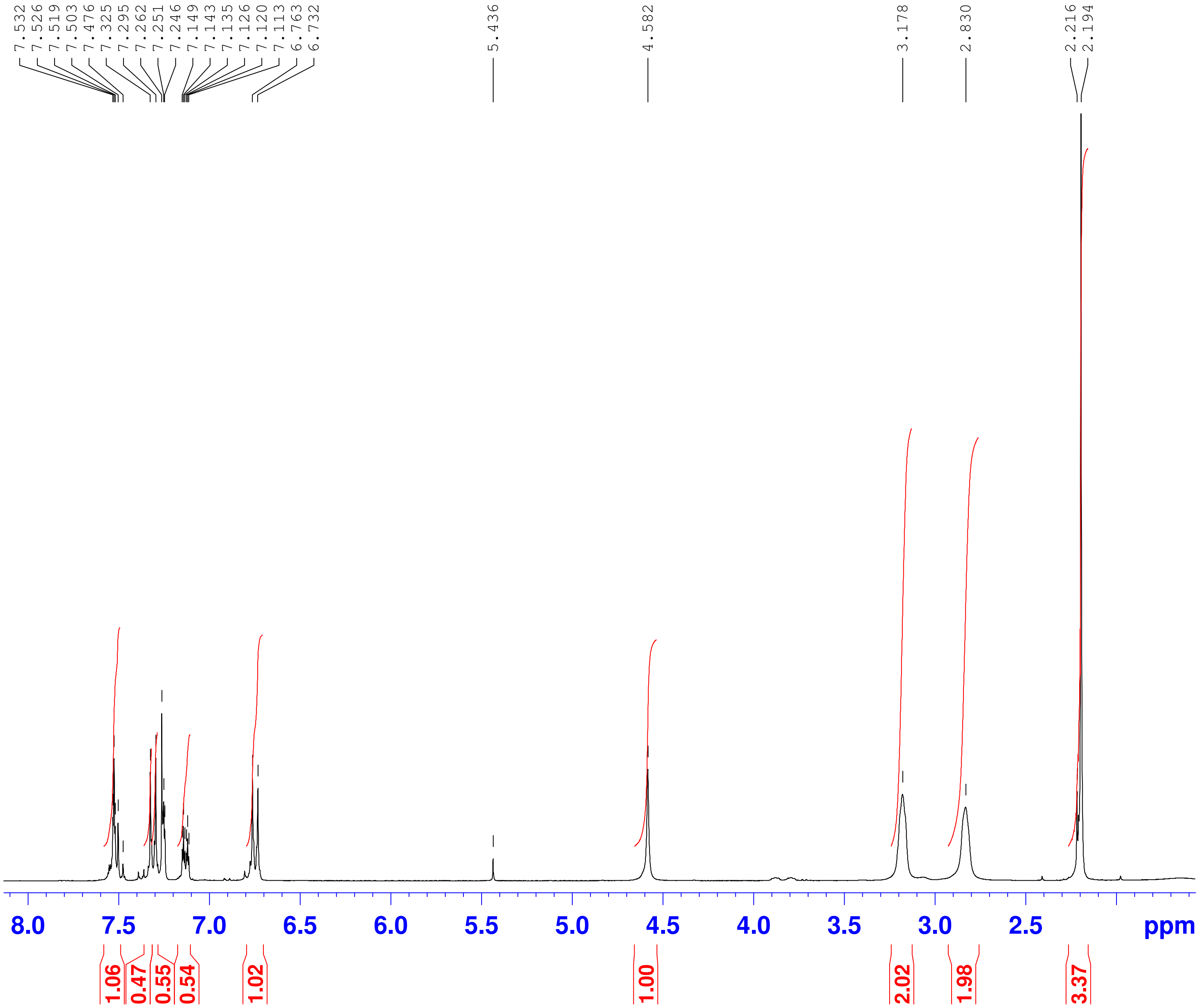


```
Current Data Parameters
NAME        Redzicka 6328
EXPNO                   1
PROCNO                  1

F2 - Acquisition Parameters
Date_            20201217
Time                16.47
INSTRUM             spect
PROBHD    5 mm BBI 1H/D-
PULPROG              zg30
TD                  65536
SOLVENT             CDCl3
NS                     16
DS                      0
SWH              6172.839 Hz
FIDRES           0.094190 Hz
AQ              5.3084660 sec
RG                  287.4
DW                 81.000 usec
DE                   8.00 usec
TE                  296.9 K
D1             1.00000000 sec
TD0                     1

======== CHANNEL f1 ========
NUC1                   1H
P1                  10.40 usec
PL1                  2.00 dB
SFO1          300.1518535 MHz

F2 - Processing parameters
SI                  32768
SF            300.1500045 MHz
WDW                    EM
SSB                     0
LB                   0.30 Hz
GB                      0
PC                  20.00
```

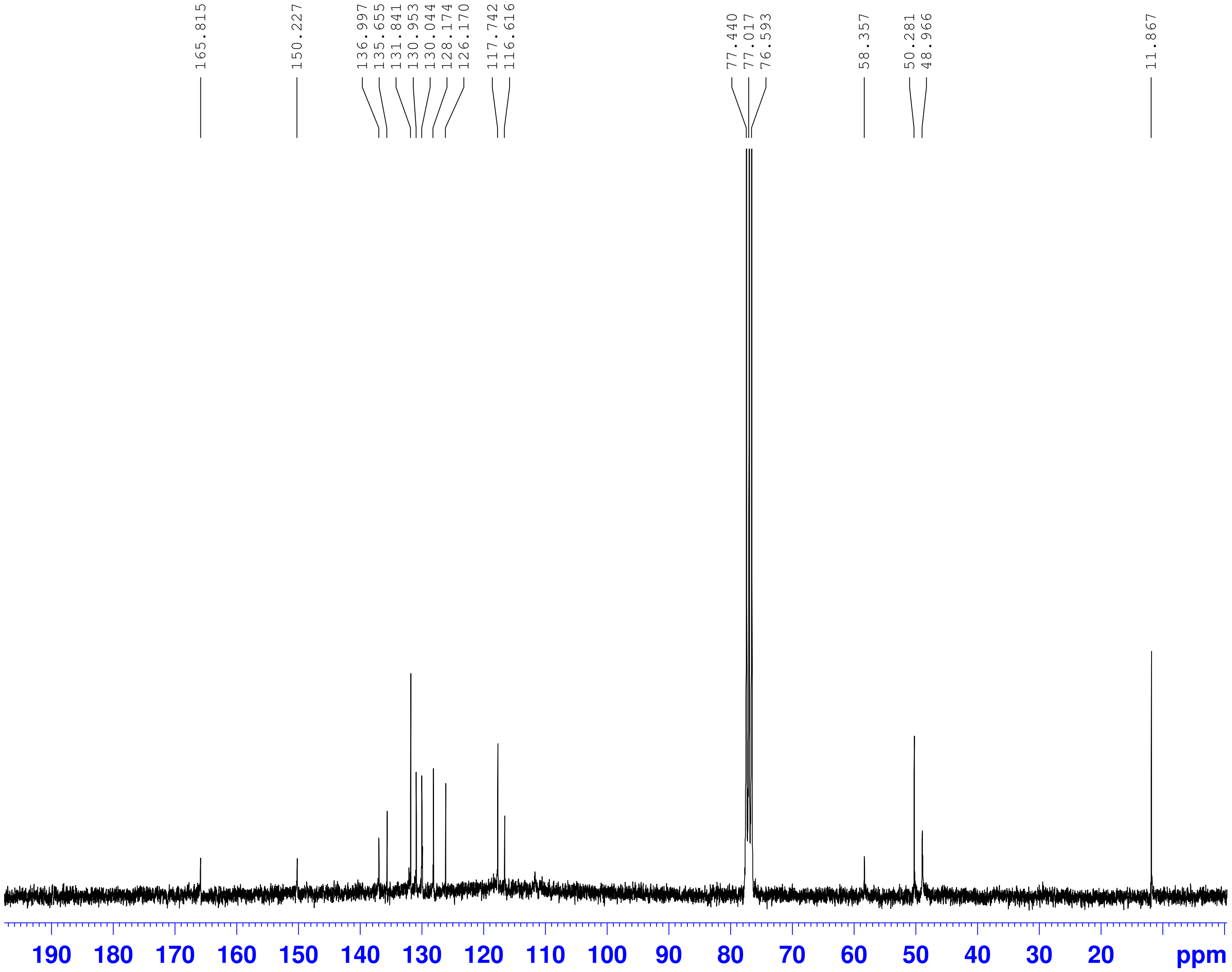

Current Data Parameters
NAME Redzicka 6328
EXPNO 2
PROCNO 1

F2 - Acquisition Parameters
Date_ 20201217
Time 22.20
INSTRUM spect
PROBHD 5 mm BBI 1H/D-
PULPROG zgpg30
TD 65536
SOLVENT CDCl3
NS 5120
DS 2
SWH 17985.611 Hz
FIDRES 0.274439 Hz
AQ 1.8219508 sec
RG 16384
DW 27.800 usec
DE 20.00 usec
TE 297.9 K
D1 2.00000000 sec
d11 0.03000000 sec
DELTA 1.89999998 sec
TD0 1

======== CHANNEL f1 ========
NUC1 13C
P1 11.90 usec
PL1 -6.00 dB
SFO1 75.4803248 MHz

======== CHANNEL f2 ========
CPDPRG2 waltz16
NUC2 1H
PCPD2 100.00 usec
PL2 2.00 dB
PL12 21.66 dB
PL13 23.00 dB
SFO2 300.1512006 MHz

F2 - Processing parameters
SI 32768
SF 75.4727782 MHz
WDW EM
SSB 0
LB 1.00 Hz
GB 0
PC 2.00

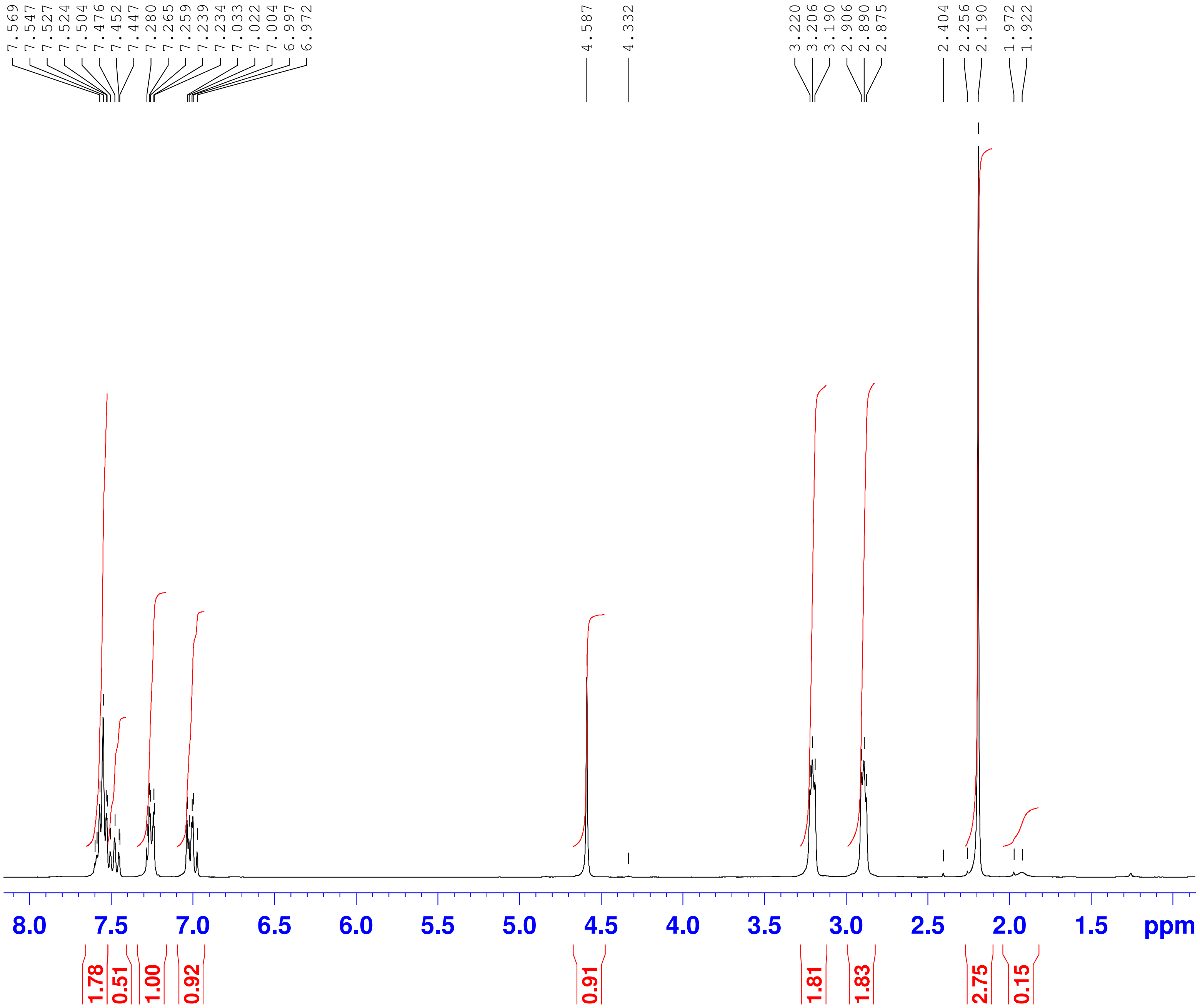

BRUKER

```
Current Data Parameters
NAME       Redzicka 4902
EXPNO                  1
PROCNO                 1

F2 - Acquisition Parameters
Date_           20180830
Time               11.39
INSTRUM            spect
PROBHD   5 mm BBI 1H/D-
PULPROG             zg30
TD                 65536
SOLVENT            CDCl3
NS                    16
DS                     0
SWH          6172.839 Hz
FIDRES       0.094190 Hz
AQ          5.3084660 sec
RG                  71.8
DW            81.000 usec
DE              8.00 usec
TE               298.9 K
D1        1.00000000 sec
TD0                    1

======== CHANNEL f1 ========
NUC1                  1H
P1             11.00 usec
PL1               2.50 dB
SFO1      300.1518535 MHz

F2 - Processing parameters
SI                 32768
SF        300.1500000 MHz
WDW                   EM
SSB                    0
LB               0.30 Hz
GB                     0
PC                 20.00
```

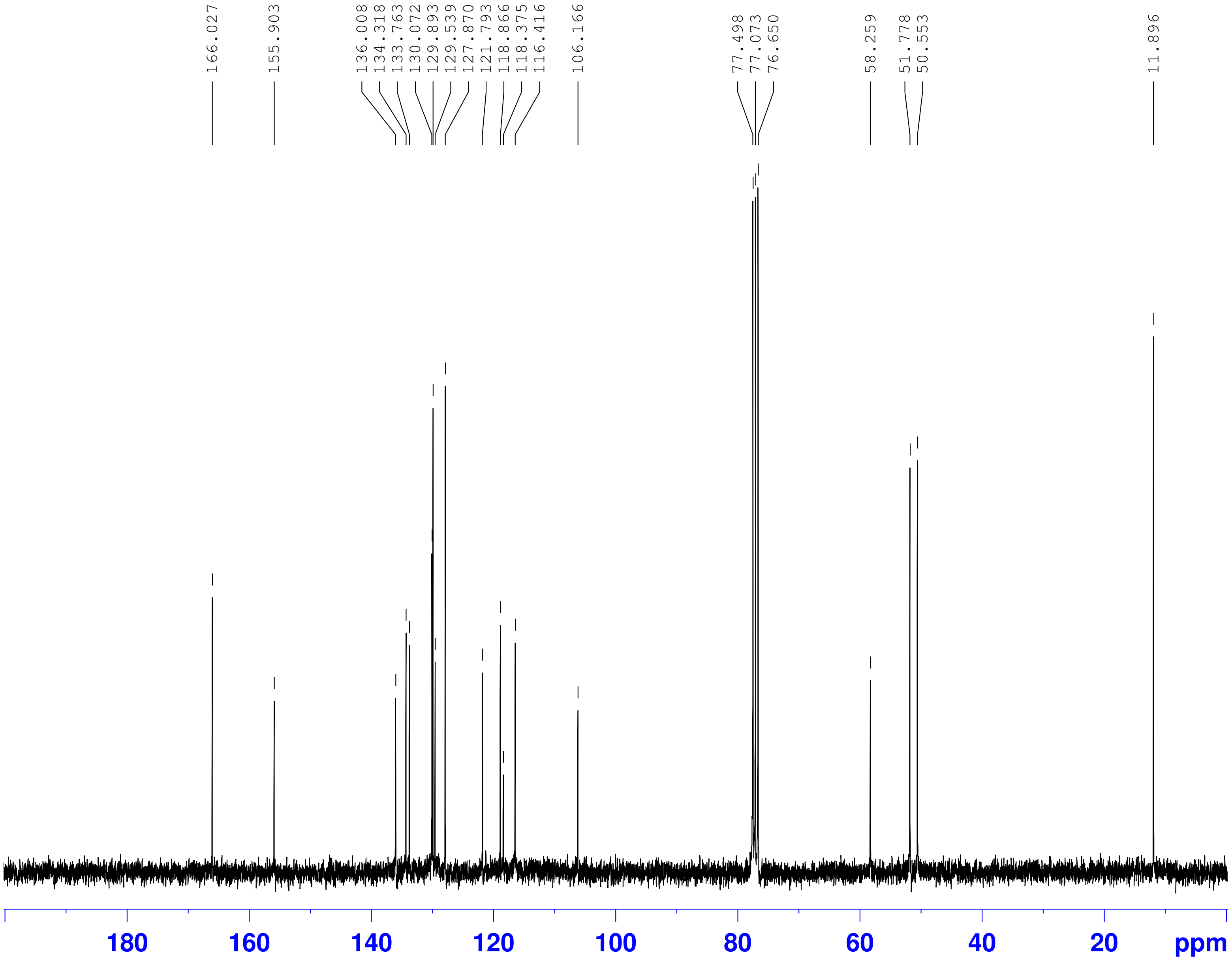


```
Current Data Parameters
NAME       Redzicka 4902
EXPNO                  2
PROCNO                 1

F2 - Acquisition Parameters
Date_           20180830
Time               12.13
INSTRUM            spect
PROBHD   5 mm BBI 1H/D-
PULPROG           zgpg30
TD                 65536
SOLVENT            CDCl3
NS                   512
DS                     2
SWH            17985.611 Hz
FIDRES          0.274439 Hz
AQ             1.8219508 sec
RG               18390.4
DW                27.800 usec
DE                 20.00 usec
TE                 299.2 K
D1            2.00000000 sec
d11           0.03000000 sec
DELTA         1.89999998 sec
TD0                    1

======== CHANNEL f1 ========
NUC1                 13C
P1                 11.00 usec
PL1                -6.00 dB
SFO1          75.4803248 MHz

======== CHANNEL f2 ========
CPDPRG2          waltz16
NUC2                  1H
PCPD2             100.00 usec
PL2                 2.00 dB
PL12               21.45 dB
PL13               23.00 dB
SFO2         300.1512006 MHz

F2 - Processing parameters
SI                 32768
SF            75.4727780 MHz
WDW                   EM
SSB                    0
LB                  1.00 Hz
GB                     0
PC                  2.00
```

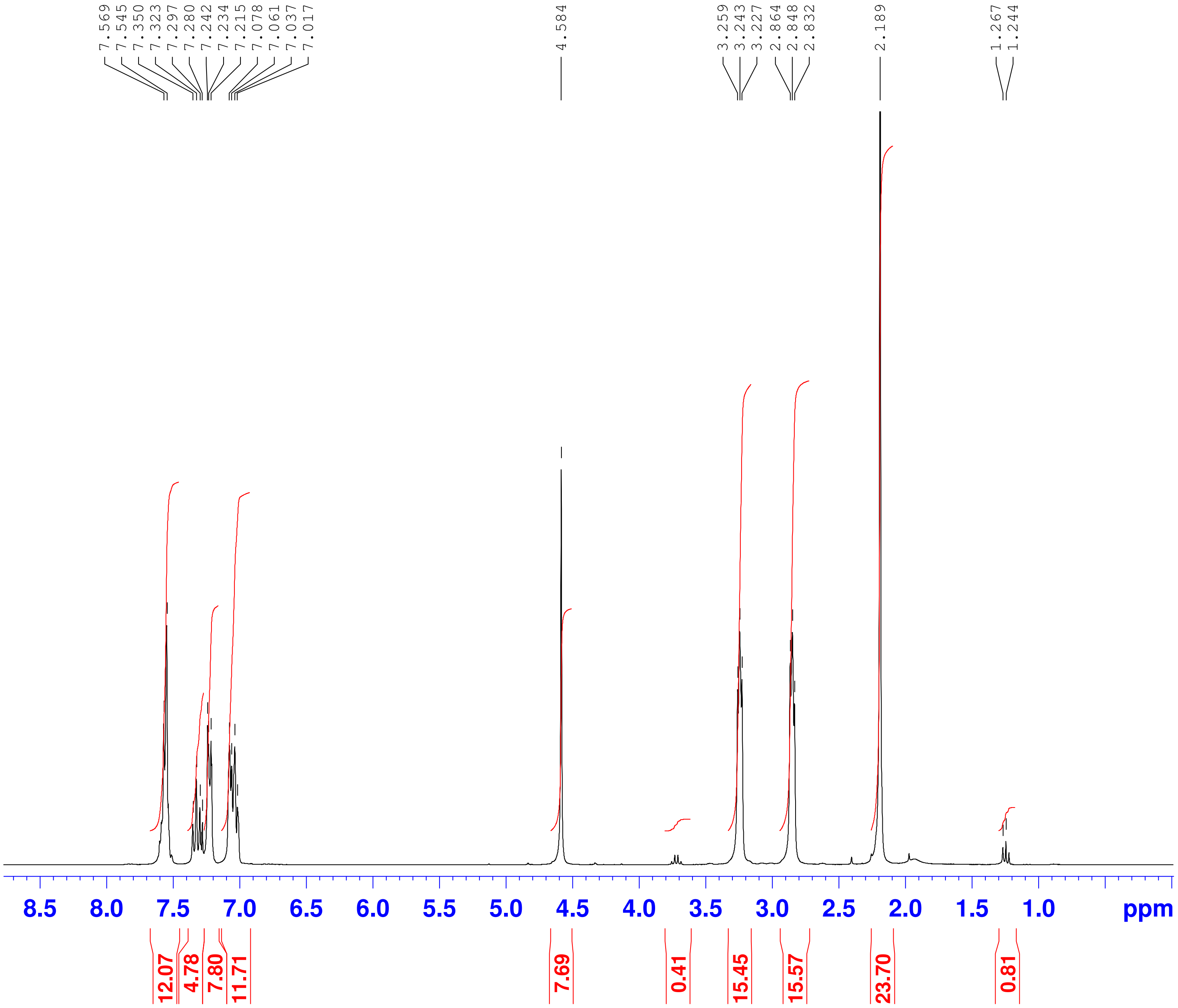

BRUKER

Current Data Parameters
NAME Redzicka 4908
EXPNO 1
PROCNO 1

F2 - Acquisition Parameters
Date_ 20180830
Time 10.52
INSTRUM spect
PROBHD 5 mm BBI 1H/D-
PULPROG zg30
TD 65536
SOLVENT CDCl3
NS 16
DS 0
SWH 6172.839 Hz
FIDRES 0.094190 Hz
AQ 5.3084660 sec
RG 71.8
DW 81.000 usec
DE 8.00 usec
TE 298.9 K
D1 1.00000000 sec
TD0 1

======== CHANNEL f1 ========
NUC1 1H
P1 11.00 usec
PL1 2.50 dB
SFO1 300.1518535 MHz

F2 - Processing parameters
SI 32768
SF 300.1500000 MHz
WDW EM
SSB 0
LB 0.30 Hz
GB 0
PC 20.00

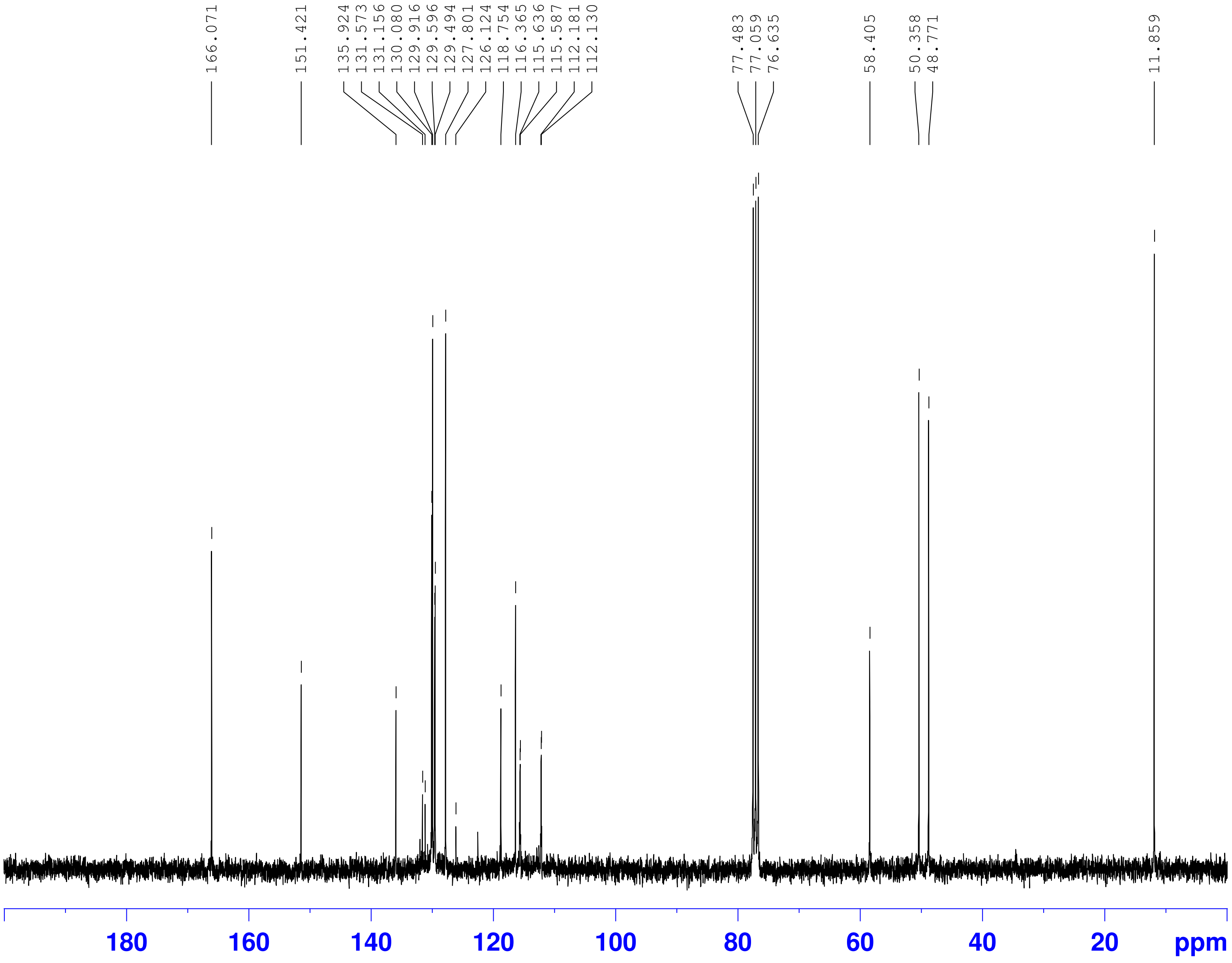


```
Current Data Parameters
NAME        Redzicka 4908
EXPNO                   2
PROCNO                  1

F2 - Acquisition Parameters
Date_            20180830
Time                11.26
INSTRUM             spect
PROBHD    5 mm BBI 1H/D-
PULPROG           zgpg30
TD                  65536
SOLVENT             CDCl3
NS                    512
DS                      2
SWH             17985.611 Hz
FIDRES           0.274439 Hz
AQ              1.8219508 sec
RG                  13004
DW                 27.800 usec
DE                  20.00 usec
TE                  299.1 K
D1             2.00000000 sec
d11            0.03000000 sec
DELTA          1.89999998 sec
TD0                     1

======== CHANNEL f1 ========
NUC1                  13C
P1                  11.00 usec
PL1                 -6.00 dB
SFO1           75.4803248 MHz

======== CHANNEL f2 ========
CPDPRG2           waltz16
NUC2                   1H
PCPD2              100.00 usec
PL2                  2.00 dB
PL12                21.45 dB
PL13                23.00 dB
SFO2          300.1512006 MHz

F2 - Processing parameters
SI                  32768
SF             75.4727780 MHz
WDW                    EM
SSB                     0
LB                   1.00 Hz
GB                      0
PC                   2.00
```

Redzicka 4559

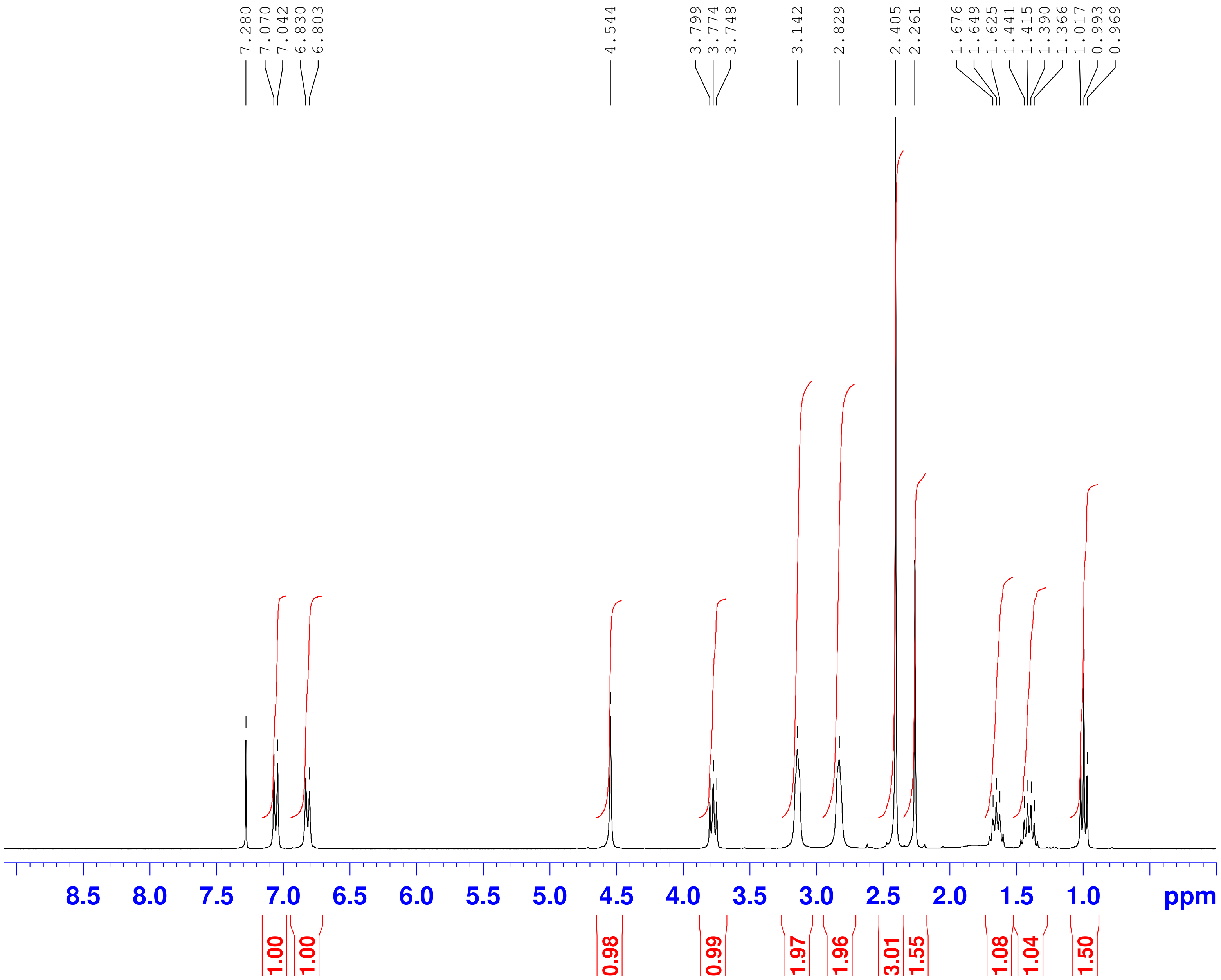

BRUKER

Current Data Parameters
NAME Redzicka 4559
EXPNO 1
PROCNO 1

F2 - Acquisition Parameters

Date_	20180214
Time	10.30
INSTRUM	spect
PROBHD	5 mm BBI 1H/D-
PULPROG	zg30
TD	65536
SOLVENT	CDCl3
NS	16
DS	0
SWH	6172.839 Hz
FIDRES	0.094190 Hz
AQ	5.3084660 sec
RG	181
DW	81.000 usec
DE	8.00 usec
TE	297.1 K
D1	1.00000000 sec
TD0	1

======== CHANNEL f1 ========

NUC1	1H
P1	11.00 usec
PL1	2.50 dB
SFO1	300.1518535 MHz

F2 - Processing parameters

SI	32768
SF	300.1500000 MHz
WDW	EM
SSB	0
LB	0.30 Hz
GB	0
PC	20.00

Redzicka 4559

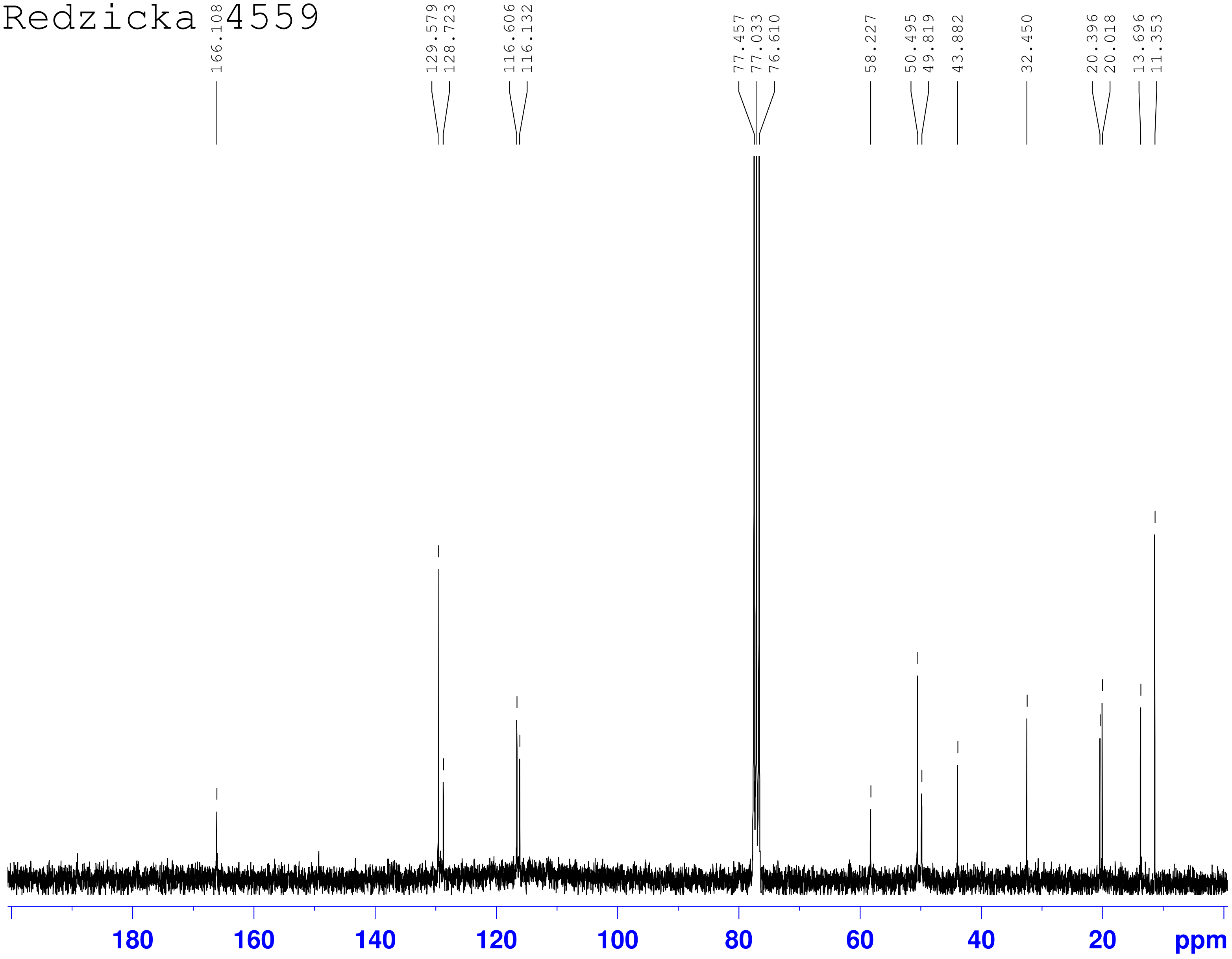

BRUKER

Current Data Parameters
NAME Redzicka 4559
EXPNO 2
PROCNO 1

F2 - Acquisition Parameters
Date_ 20180214
Time 13.50
INSTRUM spect
PROBHD 5 mm BBI 1H/D-
PULPROG zgpg30
TD 65536
SOLVENT CDCl3
NS 3072
DS 2
SWH 17985.611 Hz
FIDRES 0.274439 Hz
AQ 1.8219508 sec
RG 18390.4
DW 27.800 usec
DE 20.00 usec
TE 298.0 K
D1 2.00000000 sec
d11 0.03000000 sec
DELTA 1.89999998 sec
TD0 1

======== CHANNEL f1 ========
NUC1 13C
P1 11.00 usec
PL1 -6.00 dB
SFO1 75.4803248 MHz

======== CHANNEL f2 ========
CPDPRG2 waltz16
NUC2 1H
PCPD2 100.00 usec
PL2 2.00 dB
PL12 21.45 dB
PL13 23.00 dB
SFO2 300.1512006 MHz

F2 - Processing parameters
SI 32768
SF 75.4727780 MHz
WDW EM
SSB 0
LB 1.00 Hz
GB 0
PC 2.00

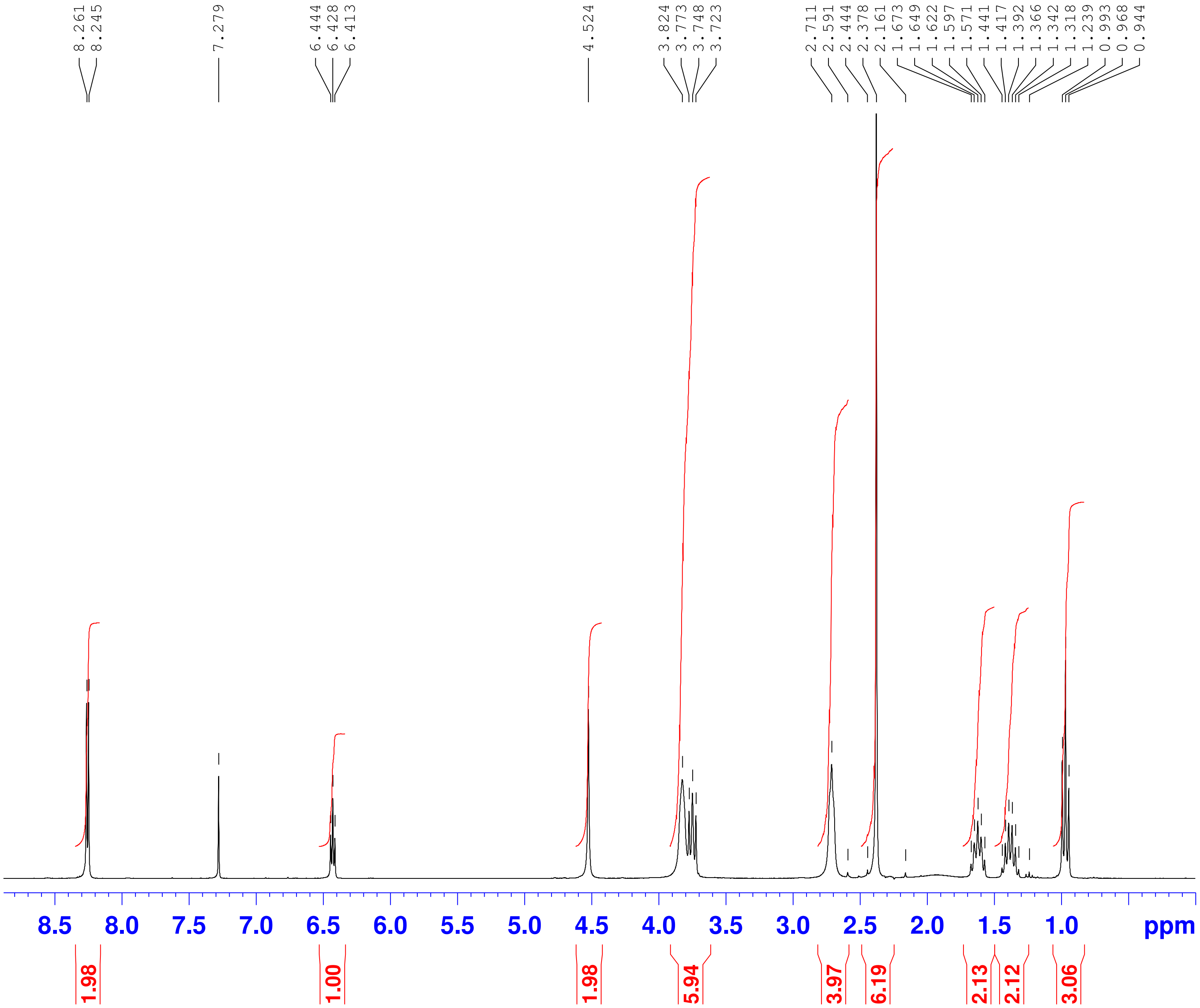

BRUKER

```
Current Data Parameters
NAME       Redzicka 4558
EXPNO                  1
PROCNO                 1

F2 - Acquisition Parameters
Date_           20180213
Time               13.01
INSTRUM            spect
PROBHD   5 mm BBI 1H/D-
PULPROG             zg30
TD                 65536
SOLVENT            CDCl3
NS                    16
DS                     0
SWH             6172.839 Hz
FIDRES          0.094190 Hz
AQ             5.3084660 sec
RG                 161.3
DW                81.000 usec
DE                  8.00 usec
TE                 297.3 K
D1            1.00000000 sec
TD0                    1

======== CHANNEL f1 ========
NUC1                  1H
P1                 11.00 usec
PL1                 2.50 dB
SFO1         300.1518535 MHz

F2 - Processing parameters
SI                 32768
SF           300.1500000 MHz
WDW                   EM
SSB                    0
LB                  0.30 Hz
GB                     0
PC                 20.00
```

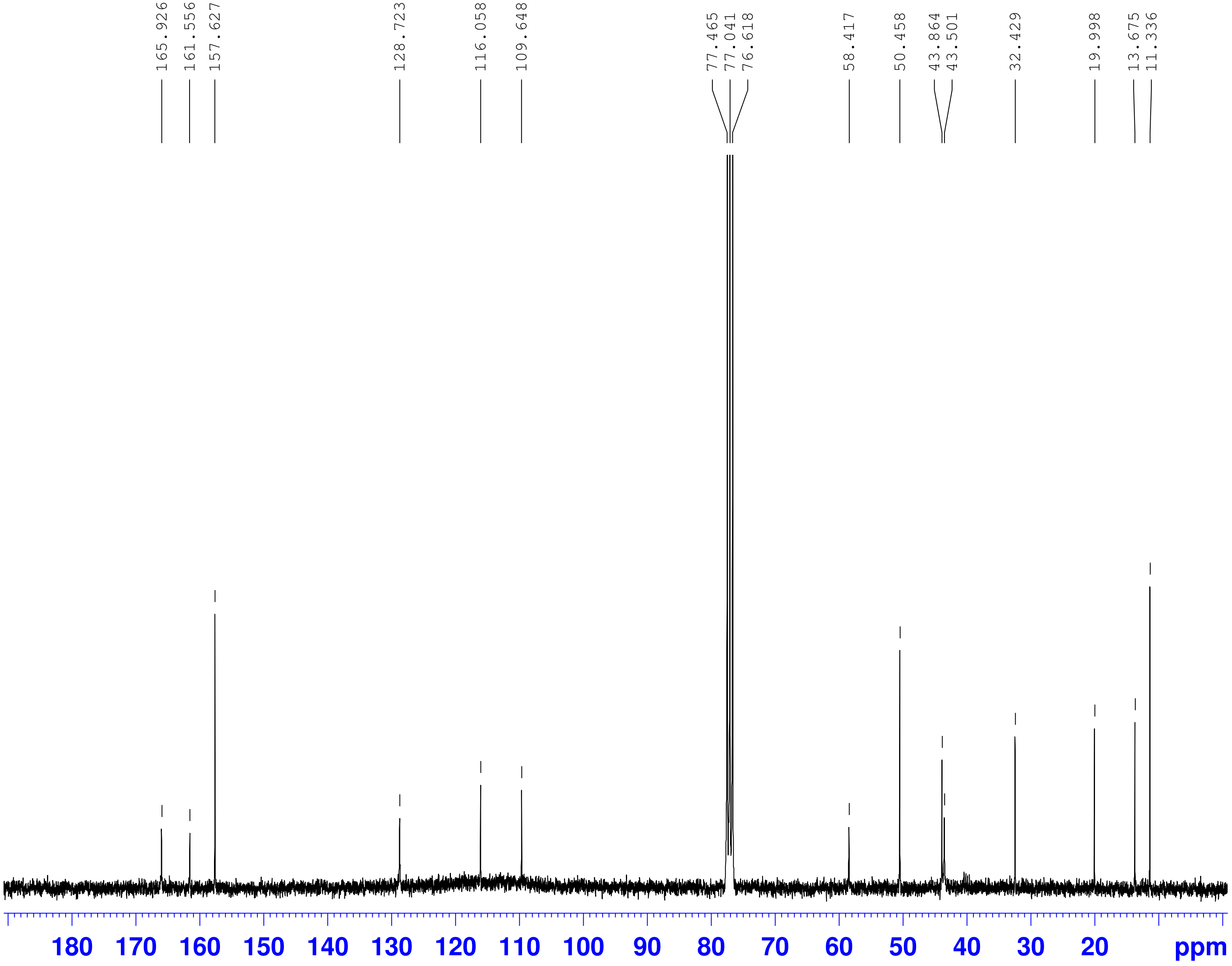


```
Current Data Parameters
NAME       Redzicka 4558
EXPNO                  2
PROCNO                 1

F2 - Acquisition Parameters
Date_           20180213
Time               18.33
INSTRUM            spect
PROBHD    5 mm BBI 1H/D-
PULPROG          zgpg30
TD                 65536
SOLVENT            CDCl3
NS                  5120
DS                     2
SWH           17985.611 Hz
FIDRES         0.274439 Hz
AQ            1.8219508 sec
RG               18390.4
DW               27.800 usec
DE                20.00 usec
TE                298.0 K
D1           2.00000000 sec
d11          0.03000000 sec
DELTA        1.89999998 sec
TD0                    1

======== CHANNEL f1 ========
NUC1                 13C
P1                11.00 usec
PL1               -6.00 dB
SFO1         75.4803248 MHz

======== CHANNEL f2 ========
CPDPRG2          waltz16
NUC2                  1H
PCPD2            100.00 usec
PL2                 2.00 dB
PL12               21.45 dB
PL13               23.00 dB
SFO2        300.1512006 MHz

F2 - Processing parameters
SI                 32768
SF            75.4727780 MHz
WDW                   EM
SSB                    0
LB                  1.00 Hz
GB                     0
PC                  2.00
```

redzicka

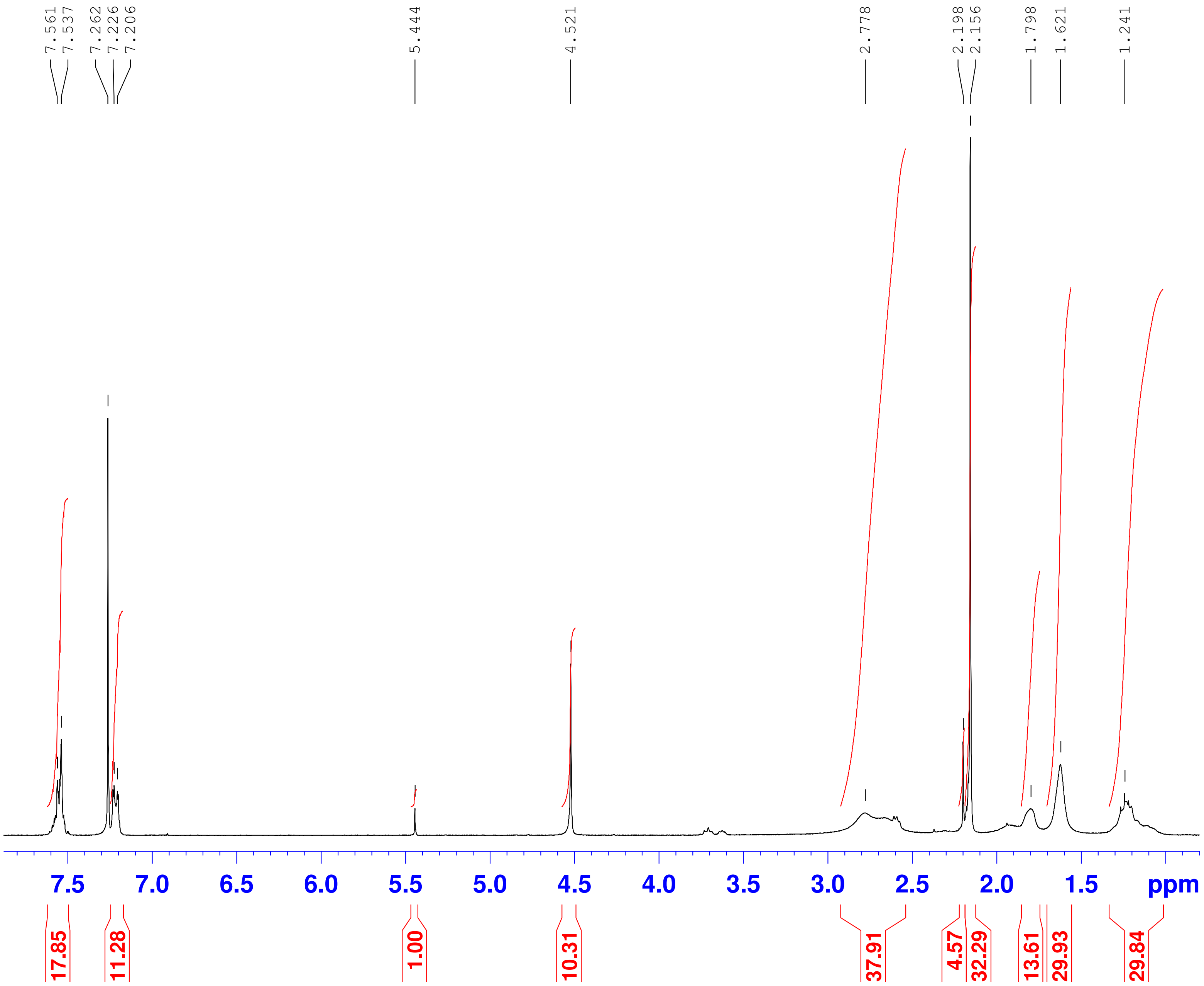


```
Current Data Parameters
NAME              redzicka
EXPNO                 6053
PROCNO                3324

F2 - Acquisition Parameters
Date_             20191106
Time                 13.43
INSTRUM              spect
PROBHD    5 mm BBI 1H/D-
PULPROG               zg30
TD                   65536
SOLVENT              CDCl3
NS                      16
DS                       0
SWH             6172.839 Hz
FIDRES          0.094190 Hz
AQ             5.3084660 sec
RG                   406.4
DW                  81.000 usec
DE                    8.00 usec
TE                   298.3 K
D1            1.00000000 sec
TD0                      1

======== CHANNEL f1 ========
NUC1                    1H
P1                   10.40 usec
PL1                   2.00 dB
SFO1           300.1518535 MHz

F2 - Processing parameters
SI                   32768
SF             300.1500045 MHz
WDW                     EM
SSB                      0
LB                    0.30 Hz
GB                       0
PC                   20.00
```

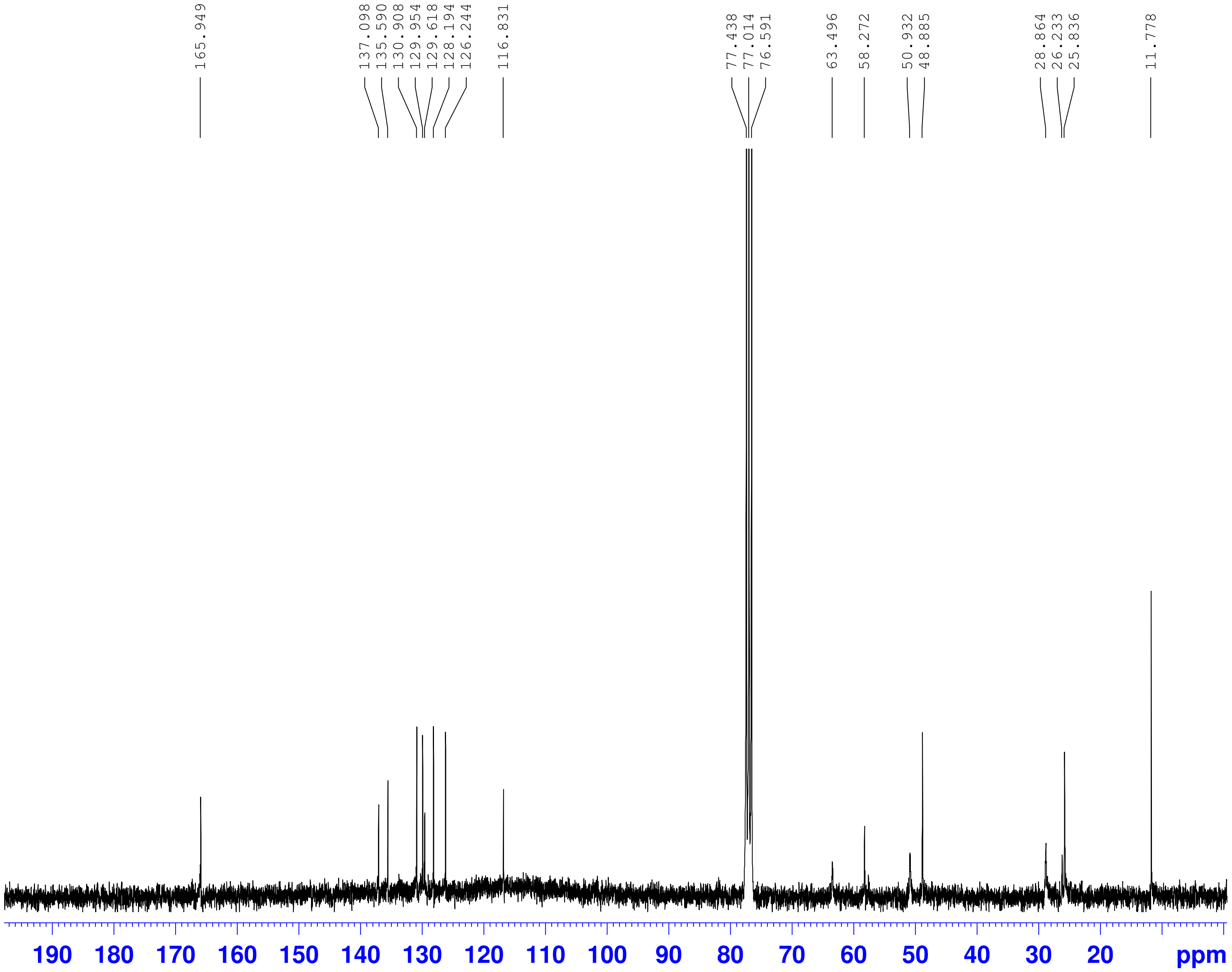

Current Data Parameters	
NAME	Redzicka 6314
EXPNO	1
PROCNO	1

F2 - Acquisition Parameters	
Date_	20201215
Time	22.22
INSTRUM	spect
PROBHD	5 mm BBI 1H/D-
PULPROG	zgpg30
TD	65536
SOLVENT	CDCl3
NS	5120
DS	2
SWH	17985.611 Hz
FIDRES	0.274439 Hz
AQ	1.8219508 sec
RG	13004
DW	27.800 usec
DE	20.00 usec
TE	373.1 K
D1	2.00000000 sec
d11	0.03000000 sec
DELTA	1.89999998 sec
TD0	1

======== CHANNEL f1 ========	
NUC1	13C
P1	11.90 usec
PL1	-6.00 dB
SFO1	75.4803248 MHz

======== CHANNEL f2 ========	
CPDPRG2	waltz16
NUC2	1H
PCPD2	100.00 usec
PL2	2.00 dB
PL12	21.66 dB
PL13	23.00 dB
SFO2	300.1512006 MHz

F2 - Processing parameters	
SI	32768
SF	75.4727782 MHz
WDW	EM
SSB	0
LB	1.00 Hz
GB	0
PC	2.00

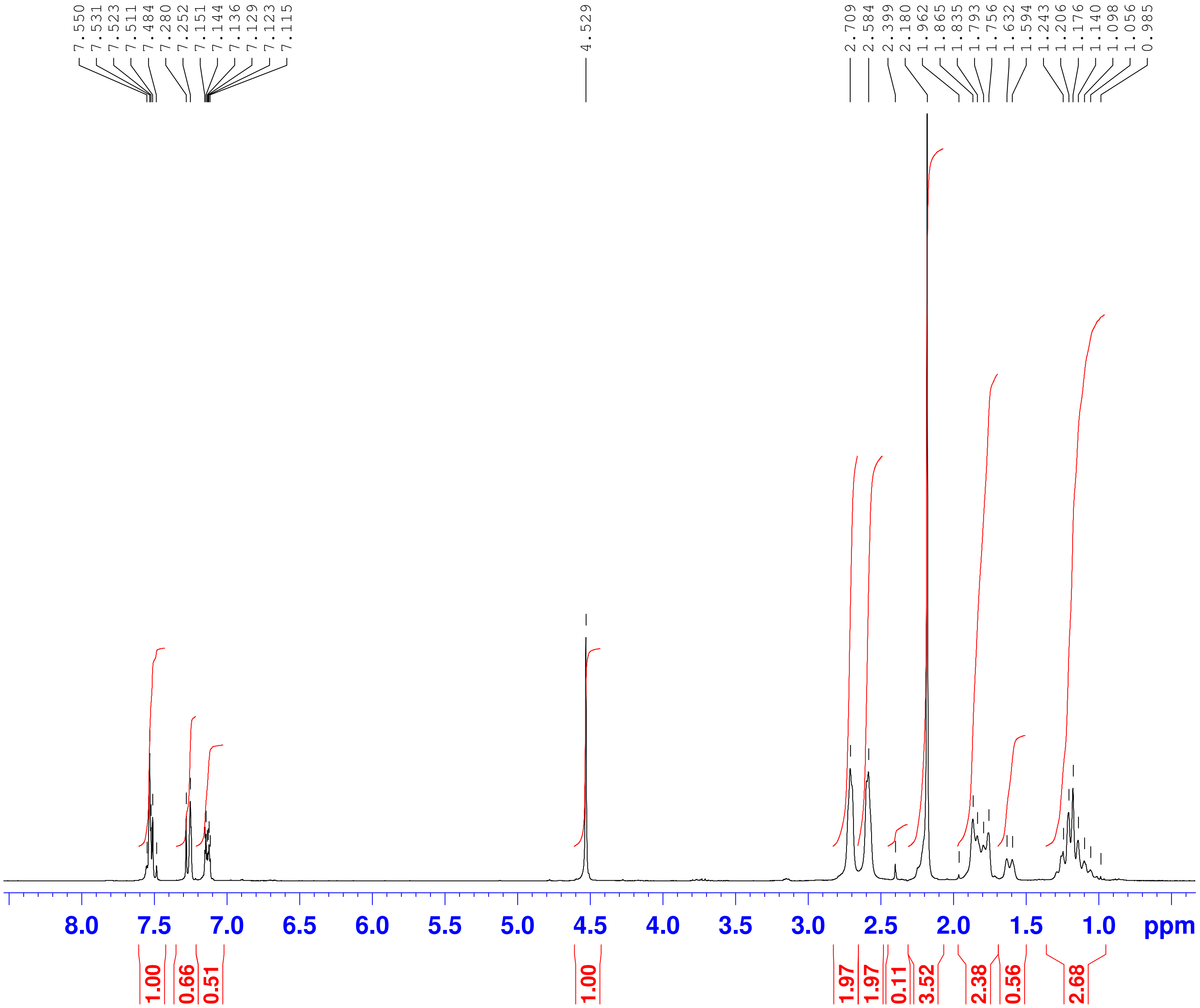

BRUKER

Current Data Parameters
NAME Redzicka 4901
EXPNO 1
PROCNO 1

F2 - Acquisition Parameters
Date_ 20180830
Time 13.11
INSTRUM spect
PROBHD 5 mm BBI 1H/D-
PULPROG zg30
TD 65536
SOLVENT CDCl3
NS 16
DS 0
SWH 6172.839 Hz
FIDRES 0.094190 Hz
AQ 5.3084660 sec
RG 114
DW 81.000 usec
DE 8.00 usec
TE 299.0 K
D1 1.00000000 sec
TD0 1

======== CHANNEL f1 ========
NUC1 1H
P1 11.00 usec
PL1 2.50 dB
SFO1 300.1518535 MHz

F2 - Processing parameters
SI 32768
SF 300.1500000 MHz
WDW EM
SSB 0
LB 0.30 Hz
GB 0
PC 20.00

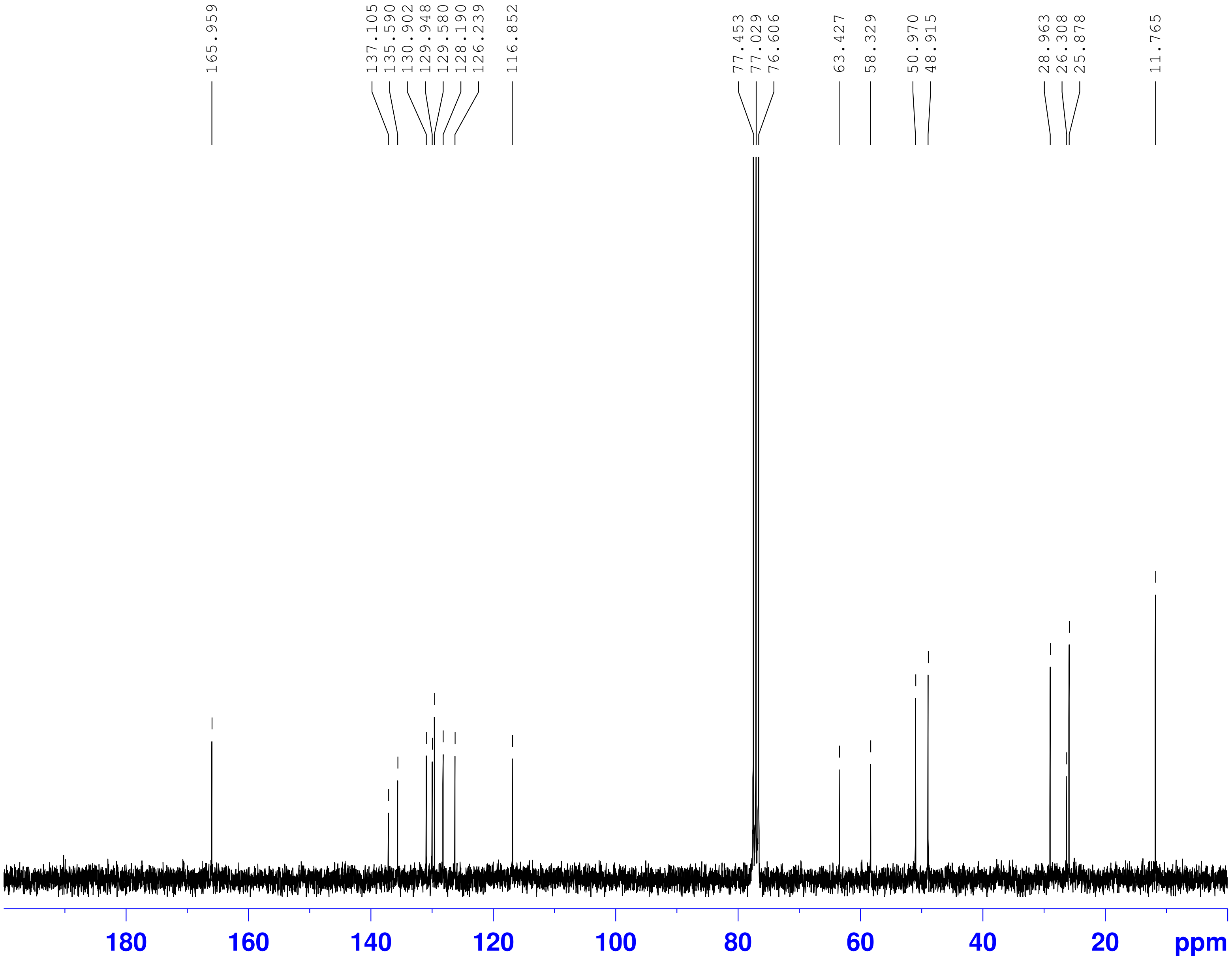

Current Data Parameters
NAME Redzicka 4901
EXPNO 2
PROCNO 1

F2 - Acquisition Parameters
Date_ 20180830
Time 13.45
INSTRUM spect
PROBHD 5 mm BBI 1H/D-
PULPROG zgpg30
TD 65536
SOLVENT CDCl3
NS 512
DS 2
SWH 17985.611 Hz
FIDRES 0.274439 Hz
AQ 1.8219508 sec
RG 18390.4
DW 27.800 usec
DE 20.00 usec
TE 299.2 K
D1 2.00000000 sec
d11 0.03000000 sec
DELTA 1.89999998 sec
TD0 1

======== CHANNEL f1 ========
NUC1 13C
P1 11.00 usec
PL1 -6.00 dB
SFO1 75.4803248 MHz

======== CHANNEL f2 ========
CPDPRG2 waltz16
NUC2 1H
PCPD2 100.00 usec
PL2 2.00 dB
PL12 21.45 dB
PL13 23.00 dB
SFO2 300.1512006 MHz

F2 - Processing parameters
SI 32768
SF 75.4727780 MHz
WDW EM
SSB 0
LB 1.00 Hz
GB 0
PC 2.00
