# Supplementary material for: Design and Synthesis of N-Substituted 3,4-Pyrroledicarboximides as Potential Anti-Inflammatory Agents
Source: Int J Mol Sci. 2021 Jan 30;22(3):1410. doi: 10.3390/ijms22031410 (PMC7866801; doi:10.3390/ijms22031410)
Supplement: Supplementary file 1 [file ijms-22-01410-s001.zip › crystal2h/checkCIF(2).pdf]

checkCIF (full publication check) running

Checking for embedded fcf data in CIF ...
Found embedded fcf data in CIF. Extracting fcf data from uploaded CIF, please wait . .

checkCIF/PLATON (full publication check)

Structure factors have been supplied for datablock(s) I

THIS REPORT IS FOR GUIDANCE ONLY. IF USED AS PART OF A REVIEW PROCEDURE FOR PUBLICATION, IT SHOULD NOT REPLACE THE EXPERTISE OF AN EXPERIENCED CRYSTALLOGRAPHIC REFEREE.

No syntax errors found. CIF dictionary
Please wait while processing .... Interpreting this report

Structure factor report

Datablock: I

Bond precision:	C-C = 0.0041 A		Wavelength=1.54180
Cell:	a=8.9619(3)	b=10.2613(3)	c=14.5659(4)
	alpha=88.746(3)	beta=73.072(2)	gamma=64.597(3)
Temperature:	100 K		

	Calculated	Reported
Volume	1149.30(7)	1149.30(7)
Space group	P -1	P -1
Hall group	-P 1	-P 1
Moiety formula	C25 H31 Cl N4 O2	C25 H31 Cl N4 O2
Sum formula	C25 H31 Cl N4 O2	C25 H31 Cl N4 O2
Mr	454.99	454.99
Dx,g cm-3	1.315	1.315
Z	2	2
Mu (mm-1)	1.707	1.707
F000	484.0	484.0
F000'	486.00	
h,k,lmax	10,12,17	10,12,17
Nref	4198	4065
Tmin,Tmax	0.774,0.815	0.890,0.910
Tmin'	0.774	

Correction method= # Reported T Limits: Tmin=0.890 Tmax=0.910 AbsCorr = ANALYTICAL

Data completeness= 0.968 Theta(max)= 68.060

R(reflections)= 0.0577(3601) wR2(reflections)= 0.1710(4065)

S = 1.008 Npar= 289

The following ALERTS were generated. Each ALERT has the format
        test-name_ALERT_alert-type_alert-level.
Click on the hyperlinks for more details of the test.

Alert level C

PLAT029_ALERT_3_C _diffrn_measured_fraction_theta_full value Low .
0.969 Why?
PLAT340_ALERT_3_C Low Bond Precision on C-C Bonds ....
0.0041 Ang.
PLAT906_ALERT_3_C Large K Value in the Analysis of Variance ......
2.612 Check
PLAT911_ALERT_3_C Missing FCF Refl Between Thmin & STh/L=   0.600
103 Report
PLAT934_ALERT_3_C Number of (Iobs-Icalc)/Sigma(W) > 10 Outliers ..
1 Check

Alert level G

PLAT434_ALERT_2_G Short Inter HL..HL Contact Cl1   ..Cl1
3.29 Ang.
                                            -x,2-y,2-z =   2_577
Check
PLAT802_ALERT_4_G CIF Input Record(s) with more than 80 Characters
1 Info
PLAT883_ALERT_1_G No Info/Value for _atom_sites_solution_primary .
Please Do !
PLAT909_ALERT_3_G Percentage of I>2sig(I) Data at Theta(Max) Still
84% Note
PLAT910_ALERT_3_G Missing # of FCF Reflection(s) Below Theta(Min).
1 Note
PLAT912_ALERT_4_G Missing # of FCF Reflections Above STh/L= 0.600
29 Note
PLAT941_ALERT_3_G Average HKL Measurement Multiplicity ....
1.9 Low
PLAT965_ALERT_2_G The SHELXL WEIGHT Optimisation has not Converged
Please Check
PLAT978_ALERT_2_G Number C-C Bonds with Positive Residual Density.
2 Info

---

   0 **ALERT level A** = Most likely a serious problem - resolve or explain
   0 **ALERT level B** = A potentially serious problem, consider carefully
   5 **ALERT level C** = Check. Ensure it is not caused by an omission or
oversight
   9 **ALERT level G** = General information/check it is not something
unexpected

   1 ALERT type 1 CIF construction/syntax error, inconsistent or missing
data
   3 ALERT type 2 Indicator that the structure model may be wrong or
deficient
   8 ALERT type 3 Indicator that the structure quality may be low
   2 ALERT type 4 Improvement, methodology, query or suggestion
   0 ALERT type 5 Informative message, check

---

checkCIF publication errors

Alert level A

PUBL004_ALERT_1_A The contact author's name and address are missing,
          _publ_contact_author_name and _publ_contact_author_address.
PUBL005_ALERT_1_A _publ_contact_author_email, _publ_contact_author_fax and

```
            _publ_contact_author_phone are all missing.
            At least one of these should be present.
PUBL006_ALERT_1_A  _publ_requested_journal is missing
            e.g. 'Acta Crystallographica Section C'
PUBL008_ALERT_1_A  _publ_section_title is missing. Title of paper.
PUBL009_ALERT_1_A  _publ_author_name is missing. List of author(s) name(s).
PUBL010_ALERT_1_A  _publ_author_address is missing. Author(s) address(es).
PUBL012_ALERT_1_A  _publ_section_abstract is missing.
            Abstract of paper in English.
```

Alert level G

```
PUBL017_ALERT_1_G The _publ_section_references section is missing or
            empty.
```

```
   7 ALERT level A = Data missing that is essential or data in wrong format
   1 ALERT level G = General alerts. Data that may be required is missing
```

Publication of your CIF

You should attempt to resolve as many as possible of the alerts in all categories. Often the minor alerts point to easily fixed oversights, errors and omissions in your CIF or refinement strategy, so attention to these fine details can be worthwhile. In order to resolve some of the more serious problems it may be necessary to carry out additional measurements or structure refinements. However, the nature of your study may justify the reported deviations from journal submission requirements and the more serious of these should be commented upon in the discussion or experimental section of a paper or in the "special_details" fields of the CIF. *checkCIF* was carefully designed to identify outliers and unusual parameters, but every test has its limitations and alerts that are not important in a particular case may appear. Conversely, the absence of alerts does not guarantee there are no aspects of the results needing attention. It is up to the individual to critically assess their own results and, if necessary, seek expert advice.

If level A alerts remain, which you believe to be justified deviations, and you intend to submit this CIF for publication in a journal, you should additionally insert an explanation in your CIF using the Validation Reply Form (VRF) below. This will allow your explanation to be considered as part of the review process.

If you wish to submit your CIF for publication in Acta Crystallographica Section C or E, you should upload your CIF via the web. If you wish to submit your CIF for publication in IUCrData you should upload your CIF via the web. If your CIF is to form part of a submission to another IUCr journal, you will be asked, either during electronic submission or by the Co-editor handling your paper, to upload your CIF via our web site.

PLATON version of 18/09/2020; check.def file version of 20/08/2020

Datablock I - ellipsoid plot

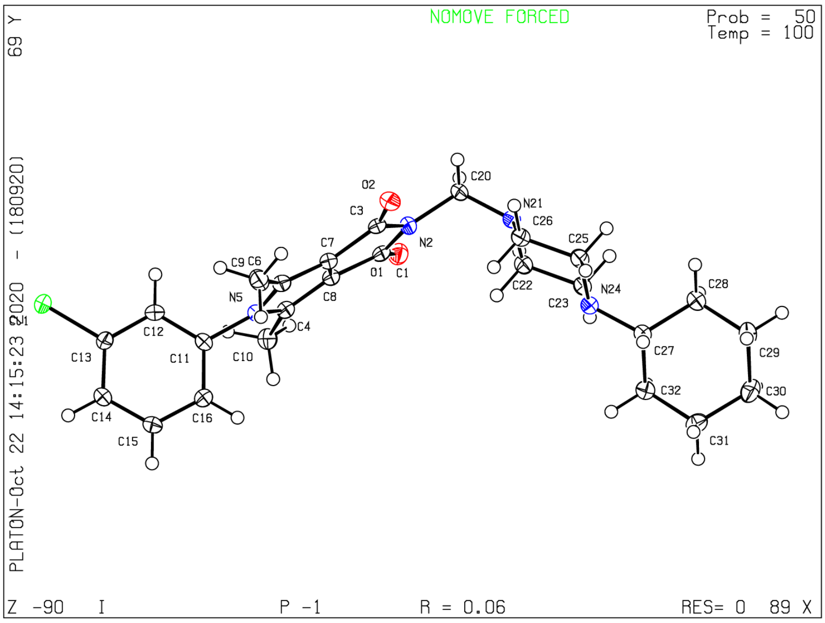

[Download CIF editor (publCIF) from the IUCr]
[Download CIF editor (enCIFer) from the CCDC]
[Test a new CIF entry]
